# Rare genetic variant risks in patients with sepsis-associated acute respiratory distress syndrome

**DOI:** 10.1186/s12931-026-03588-4

**Published:** 2026-02-25

**Authors:** Eva Tosco-Herrera, Luis A. Rubio-Rodríguez, Adrián Muñoz-Barrera, David Jáspez, Eva Suárez-Pajes, Almudena Corrales, Aitana Alonso-González, Miryam Prieto-González, Aurelio Rodríguez-Pérez, Demetrio Carriedo, Jesús Blanco, Alfonso Ambrós, Leonardo Lorente, María M. Martín, Jordi Solé-Violán, Carlos Rodríguez-Gallego, Elena González-Higueras, Elena Espinosa, Arturo Muriel-Bombin, David Domínguez, Marina Soro, Tamara Hernández-Beeftink, José M. Añón, Jesús Villar, Beatriz Guillén-Guio, Itahisa Marcelino-Rodríguez, José M. Lorenzo-Salazar, Rafaela González-Montelongo, Carlos Flores

**Affiliations:** 1 Research Unit, Hospital Universitario Nuestra Señora de Candelaria, Instituto de Investigación Sanitaria de Canarias (IISC), Santa Cruz de Tenerife, Spain; 2 Genomics Division, Instituto Tecnológico y de Energías Renovables (ITER), Santa Cruz de Tenerife, Spain; 3 CIBER de Enfermedades Respiratorias (CIBERES), Instituto de Salud Carlos III, Madrid, Spain; 4 Intensive Care Unit, Complejo Asistencial Universitario de Palencia, Palencia, Spain; 5 Department of Anesthesiology, Hospital Universitario de Gran Canaria Dr. Negrín, Las Palmas de Gran Canaria, Spain; 6 Department of Medical and Surgical Sciences, School of Medicine, University of Las Palmas de Gran Canaria, Las Palmas de Gran Canaria, Spain; 7 Intensive Care Unit, Complejo Hospitalario Universitario de León, León, Spain; 8 Intensive Care Unit, Hospital Universitario Rio Hortega, Valladolid, Spain; 9 Intensive Care Unit, Hospital General de Ciudad Real, Ciudad Real, Spain; 10 Intensive Care Unit, Hospital Universitario de Canarias, Santa Cruz de Tenerife, Spain; 11 Intensive Care Unit, Hospital Universitario Nuestra Señora de Candelaria, Santa Cruz de Tenerife, Spain; 12 Department of Clinical Sciences, University Fernando Pessoa Canarias, Las Palmas de Gran Canaria, Spain; 13 Critical Care Unit, Hospital Universitario de Gran Canaria Dr. Negrín, Las Palmas de Gran Canaria, Spain; 14 Department of Immunology, Hospital Universitario de Gran Canaria Dr. Negrín, Instituto de Investigación Sanitaria de Canarias (IISC), Las Palmas de Gran Canaria, Spain; 15 Intensive Care Unit, Hospital Virgen de La Luz, Cuenca, Spain; 16 Department of Anesthesiology, Hospital Universitario Nuestra Señora de Candelaria, Santa Cruz de Tenerife, Spain; 17 Anesthesiology and Critical Care Department, Hospital IMED Valencia, Valencia, Spain; 18 Division of Public Health and Epidemiology, School of Medical Sciences, University of Leicester, Leicester, UK; 19 University Hospitals of Leicester NHS Trust, Leicester, UK; 20 Intensive Care Unit, Hospital Universitario La Paz, Madrid, IdiPAZ Spain; 21 Research Unit, Hospital Universitario Dr. Negrín, Las Palmas de Gran Canaria, Spain; 22 Li Ka Shing Knowledge Institute, St. Michael’s Hospital, Toronto, Canada; 23 Faculty of Health Sciences, Universidad del Atlántico Norte, Las Palmas de Gran Canaria, Spain; 24 Public Health and Preventive Medicine Area, University of La Laguna, Santa Cruz de Tenerife, Spain; 25 Instituto de Tecnologías Biomédicas (ITB), University of La Laguna, Santa Cruz de Tenerife, Spain

## Abstract

**Background:**

Acute respiratory distress syndrome (ARDS) is a complex, heterogeneous, and deadly condition often resulting from pulmonary lesions due to sepsis, among other causes. There is a lack of targeted therapies to specifically treat the patients. Common genetic factors in the population (frequency > 1%) have been associated with ARDS susceptibility, but systematic genetic screens of the role of rare genetic variants are lacking. We used the network of known molecular interactions to identify ARDS risks from clusters of biologically related genes containing qualifying variants (QVs) with frequency < 1% likely affecting function.

**Methods:**

We conducted whole-exome sequencing in sepsis patients from the GEN-SEP cohort (*n* = 822, of which 272 developed ARDS). A network-based heterogeneity clustering algorithm was used to discover significant gene clusters (*p* < 1 × 10^–5^). Gene-set enrichment analysis and logistic regression models aggregating QVs were used for cross-verification to confirm consistency and deepen understanding of the effect sizes of gene clusters.

**Results:**

We identified 19 significant clusters (*p*_lowest_ = 3.29 × 10^–10^), each containing an average of 102 genes (11.6% mean similarity). QVs in nine gene clusters were associated with sepsis-associated ARDS (*p*_lowest_ = 1 × 10^–5^) but were not associated with 28-day survival. Clusters were enriched in several biological pathways, notably the *Toll-like receptor cascades*.

**Conclusions:**

These results support a marked genetic heterogeneity underlying ARDS susceptibility and the presence of rare risk variants involving multiple biological processes that are associated with sepsis outcomes. Particularly, they underscore the importance of rare variants in genes of the *Toll-like receptor cascades* in the risk for sepsis-associated ARDS.

**Supplementary Information:**

The online version contains supplementary material available at https://doi.org/10.1186/s12931-026-03588-4.

## Introduction

Acute respiratory distress syndrome (ARDS) is a heterogeneous and complex lung condition that develops after pulmonary or systemic insults, including trauma, severe pneumonia, and sepsis [1]. The diagnosis is based on acute onset, bilateral opacities on chest radiography or CT scan (of non-cardiac origin), and a PaO2/FiO2 ratio of less than 300 mm Hg according to the latest clinical guidelines [2–4]. ARDS affects almost 25% of all critically ill adult patients requiring mechanical ventilation. The overall in-hospital mortality rate exceeds 40% [5, 6], while it increased in periods of the coronavirus disease 2019 (COVID-19) pandemic caused by severe acute respiratory syndrome coronavirus 2 (SARS-CoV-2) [7]. ARDS imposes a significant economic burden on adult intensive care unit (ICU) worldwide. However, no specific pharmacotherapy is effective for ARDS treatment [8–11]. Current patient management is based on life-supportive therapies for maintaining gas exchange and reduce inflammation.

ARDS develops from a hyperinflammatory state causing alveolar-capillary membrane dysregulation [1], leading to severe impairment of gas exchange, non-cardiogenic pulmonary edema, resulting in hypoxemia and acute respiratory failure [12]. Host conditions such as age, sex, comorbidities, and exposure to airborne pollutants are known risk factors for ARDS [13–15]. However, these factors and the clinical status poorly predict ARDS risk among patients. It is thus crucial to understand the genetic and molecular mechanisms that govern host responses to severe infections and ARDS development. This knowledge is essential for patient risk stratification and for identifying novel treatments. Host genetic factors have been increasingly recognized as affecting the severity of responses to infections. To a certain point, the genetic influence on infection severity and outcomes could be exemplified in patients with inborn errors of immunity (IEI) [16–18]. Genetic studies in COVID-19 patients show that there are both common [19, 20] and rare variants affecting gene function [21, 22] associated with severe SARS-CoV-2 infections and life-threatening disease.

Genome-wide screens in cohorts of ARDS patients are still scarce in the literature [23]. Genome-wide association studies (GWAS) in ARDS have revealed common genetic risk factors (> 1% frequency) in genes involved in cellular adhesion [24], platelet activation [25], vascular permeability [26], and immune-inflammatory processes [27]. Sequencing-based studies evaluating the spectrum of variant frequencies (< 1%) enriched for deleterious effects have focused on small cohorts of fewer than 100 patients or on familial cases affected by IEI due to genetic defects in type I interferon signaling [28–30]. The latter has also evidenced some degree of genetic overlap between IEI and ARDS [23, 31].

Given that multiple biological processes are involved in ARDS, it becomes necessary to use approaches that allow aggregating genetic information from multiple pathways to capture the genetic heterogeneity. In this study, we aimed to identify rare genetic risk factors for sepsis-associated ARDS using a gene-clustering approach. We leveraged the largest exome sequencing dataset analyzed so far for this condition and the information from biological processes for adapting to patient heterogeneity.

## Materials and methods

### Patients and ethical considerations

The study examined a cohort of patients with sepsis from different foci and etiological agents from the GEN-SEP study. These patients were enrolled between 2002 and 2019 from a network of Spanish ICUs. Except for 323 patients analyzed for the first time in this study, the rest were included in previous GWAS [26, 32]. Sepsis was clinically defined according to the Third International Consensus Definitions for Sepsis [33]. The clinical and demographic data are available in Additional file 1: Supplementary Table 1.

Initially, 995 patients were included in the cohort, but 32 patients were excluded for having incomplete clinical data, and 141 were excluded due to the DNA quantity or integrity requirements. Consequently, 822 patients remained for whom complete whole-exome sequencing (WES) data were considered for the analyses. From those, 272 patients met ARDS criteria [2]. These 272 patients were used as cases and 550 sepsis patients without ARDS during their hospitalization were considered as controls (Additional file 1: Supplementary Table 1).

The study was reviewed by The University Hospital of the Canary Islands Review Board (CHUNSC_2021-40) and the Research Ethics Committees of participating centers, and was conducted in accordance with the Declaration of Helsinki. Written informed consents were obtained from all participants or their representatives.

### Library preparation, whole-exome sequencing, and variant calling

Genomic DNA was purified from peripheral blood samples using a commercial column-based DNA extraction kit (GE Healthcare, Chicago, IL). We measured DNA concentration using the Qubit 3.0 fluorimeter with the Qubit dsDNA High Sensitivity Assay kit (Thermo Fisher Scientific, Waltham, MA). Genomic libraries were prepared using either the DNA Prep with Enrichment kit (Illumina Inc., San Diego, CA) as described elsewhere [34] or the SureSelect XT HS2 DNA Reagent Kit (Agilent Technologies, Santa Clara, CA). We then assessed library sizes on a 4200 TapeStation (Agilent Technologies) and determined their concentration using the Qubit dsDNA HS Assay kit (Thermo Fisher Scientific).

Sequencing of libraries was conducted at the Instituto Tecnológico y de Energías Renovables (ITER, Santa Cruz de Tenerife, Spain). Sequencing was performed in parallel along with 1% of a PhiX control V3 (Illumina Inc.) to an average depth of at least 100X on NextSeq 550, HiSeq 4000, or NovaSeq 6000 Sequencing Systems (Illumina Inc.) using 75 base pairs (bp) or 100 bp paired-end reads as recommended. We preprocessed the sequence reads using bcl2fastq v2.18 for demultiplexing, BWA‐MEM 0.7.15 [35] for read alignment to the GRCh37/hg19 reference, SAMtools v1.3 [36] for alignment sorting, and Picard v2.10.10 [37] for marking of duplicate reads as detailed elsewhere [26]. The GATK HaplotypeCaller v3.8 [38] was used following the Best Practices recommendations for variant calling of single-nucleotide variants (SNVs) and small insertions and deletions (indels < 50 bp) with a padding of 100 bp.

Variant quality controls were applied using BCFtools v1.16 [39] to retain only high-confidence variants in the final dataset. Variants with a PASS, depth of coverage (DP) ≥ 10, genotype quality (GQ) ≥ 20, and missingness rate (FMISS) < 0.05 were included in the study. Detected variants were annotated for population allele frequency (AF) according to gnomAD Exome v2.1 [40], RefSeq-based annotation, functional consequences, and pathogenic potential based on ClinVar and the Gene Damage Index (GDI) [41]. Pathogenicity prediction scores, such as the Combined Annotation-Dependent Depletion (CADD) score v1.6, were annotated with Ensembl Variant Effect Predictor (VEP) v.105 [42] and ANNOVAR v18.04.16 [43]. The in-house pipeline is fully described online [44]. All analyses were carried out using the TeideHPC Supercomputing facility [45] (see Additional file 1:Supplementary Methods).

### Network-based heterogeneity clustering and biological enrichment analyses

We used the Network-based heterogeneity clustering v3 (NHC) to systematically aggregate biologically proximate genes from WES data based on a predefined protein–protein interaction network [46]. We followed recommendations provided by Zhang et al., [46] to select qualifying variants (QVs) for analysis. These QVs had an AF ≤ 0.01 in non-Finnish European populations, a CADD ≥ 10, a GDI ≤ 10, and that were annotated for protein truncation prediction according to VEP (non-synonymous, missense, start/stop gain/loss, frameshift, or splicing variants). Thereafter, we refer to these as QVs-CADD10. We obtained HUGO Gene Nomenclature Committee names for QVs and used them as the input for NHC on a per-sample basis. To control for genetic heterogeneity in the cohort, the first three principal components (PCs) of common variation derived from paired SNP genotyping array data (Additional file 1:Supplementary Methods and Supplementary Fig. 1) were also included in the NHC analysis.

Following recommendations [46], gene clusters differing between cases and controls were considered significant at *p* ≤ 1 × 10^–5^. A similarity matrix based on gene-level overlap was calculated among significant clusters using the Jaccard distance. Gene interaction graphs within significant clusters were represented using Cytoscape v3.10.2 [47] to integrate the known functional interactions between gene products and the count of ARDS cases with QVs. Finally, we assessed biological pathway enrichment of each significant cluster of genes using REACTOME [48]. In this discovery stage, we retained enriched significant pathways (*p* ≤ 1 × 10^–5^) and selected the most significant as the top pathway to be representative for each cluster [46].

### Qualifying variant carrier associations with ARDS

To confirm consistency of associations and deepen understanding of the effect sizes contributed by gene clusters, we then used the QVs to identify carriers among the GEN-SEP patients, provided that at least one QV was identified in any gene from a particular cluster. However, since many of the QVs are expected to be non-pathogenic in this case, a stricter CADD score threshold ≥ 15 was used and thereafter referred to as QVs-CADD15. We restricted the analysis to significant gene clusters having minimal gene-level overlap. For this, we relied on a cutoff similarity < 20% (and choosing the most significant cluster when compared clusters had > 20%) to have a balance in the overlap between clusters while limiting the prioritization of clusters enriched in pathways that were functionally broad. This approach avoids an overly conservative statistical penalty due to gene-level similarity between clusters. Since only 10 of the significant clusters satisfied this condition, we applied a *p* < 5 × 10^–3^ (*p* = 0.05/10) threshold to declare that QVs carriers of a specific cluster were significantly associated with ARDS. For those 10 significant clusters, the association of QVs carriers with sepsis-associated ARDS was tested using binomial logistic regressions adjusted for sex, age, and Acute Physiology And Chronic Health Evaluation II (APACHE II) scores as in previous GWAS [32, 49] using R v4.3.2 [50]. Based on these, we also computed the Nagelkerke’s pseudo R^2^ approximation to estimate the genetic variance explained by the QVs in the different clusters.

A schematic overview of the analysis workflow is presented in Additional file 1: Supplementary Fig. 2.

### Sensitivity analyses

Alternative models testing the association of QVs with ARDS risk were also conducted to assess the impact of adjusting for relevant demographic and clinical variables, by adjusting for the first two main PCs of genetic heterogeneity, by lowering the AF threshold (AF ≤ 0.001), or by relying on lenient CADD scores (QVs-CADD10) as those used in the discovery stage.

Loss-of-function variants in 15 genes of the Toll-like receptor (TLR)3- and TLR7-dependent interferon response (*IFNAR1*, *IFNAR2*, *IRF3*, *IRF7*, *IRF9*, *IKBKG*, *STAT1*, *STAT2*, *TBK1*, *TICAM1*, *TRAF3*, *UNC93B1*, *TYK2*, *TLR3*, and *TLR7*) are known to cause life-threatening COVID-19 [22]. We tested if ARDS risk could be explained by QVs-CADD15 in these genes by modelling two scenarios based on binomial logistic regressions adjusted by sex, age, and APACHE II scores: i) with AF ≤ 0.01; and ii) with AF ≤ 0.001.

To rule out that associations could be due to residual confounding by genetic heterogeneity, we devised a null model by testing the association of rare synonymous variants (AF ≤ 0.01) with ARDS risk for the 10 significant clusters.

Finally, a Cox proportional hazards model was employed with a subset of 665 patients for which follow-up survival data was available (152 deaths and 513 survivors) to test associations between QVs-CADD15 carriers at those 10 gene clusters and 28-day survival using the *survival* v3.7–0 R package. We did this to rule out the possibility that the QVs carrier status was associated with mortality rather than ARDS. Two alternative survival models were used for this: i) model 1 adjusted for sex, age, and APACHE II; and ii) model 2 adjusted for sex, age, APACHE II, and ARDS status of the patient. The proportionality of Hazard Ratios (HR) assumption across covariates was verified using scaled Schoenfeld residuals. For gene clusters that did not fulfill this assumption, the *coxphw* v4.0.3 R package was applied to perform a weighted estimation of average HR and avoid the need for HR proportionality across time.

## Results

### NHC analysis of exomes in ARDS patients compared to sepsis controls

**Fig. 1:**
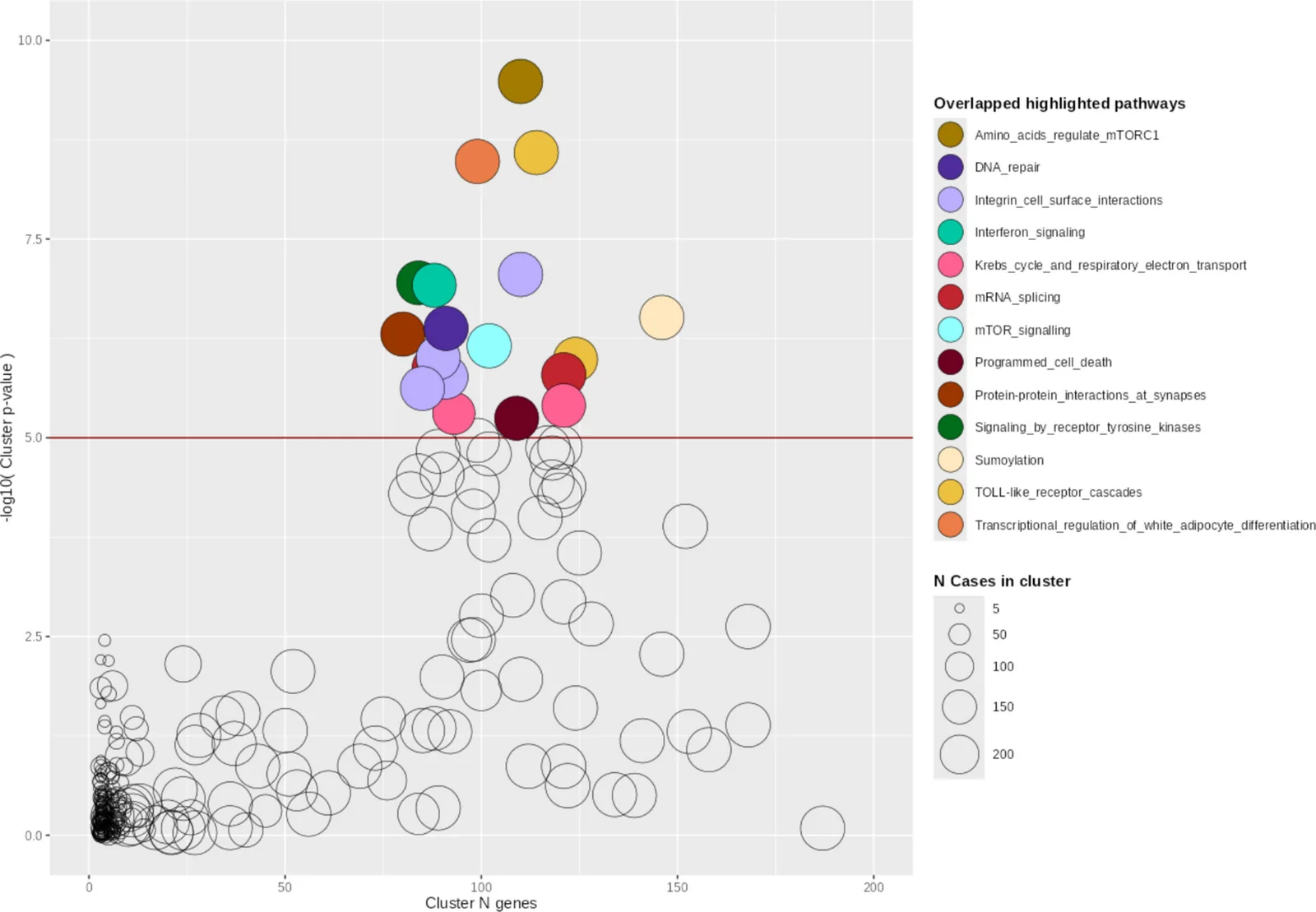
Bubble plot of gene clusters identified by the Network-based Heterogeneity Clustering analysis of ARDS cases compared to sepsis controls. Gene clusters are represented by bubbles, where the size of each is proportional to the number of ARDS cases assigned. Colors denote significant clusters (*p* ≤ 1 × 10^–5^) that share the top significant pathway. The x-axis represents the number of genes included in each cluster, and the y-axis represents the significance (-log_10_[*p*-value])

The NHC analysis revealed 19 gene clusters significantly different between ARDS cases and sepsis controls (*p*-value range = 3.29 × 10^–10^ to 5.68 × 10^–6^) (Table 1, Fig. 1). Significant clusters contained an average of 102 genes and had a mean gene-level similarity of 11.6% with other clusters (standard deviation [SD] = 22.6%) (Additional file 1: Supplementary Fig. 3). None of the genes identified in the previous sequencing-based studies of ARDS cohorts [51–53] were present in the significant gene clusters.

**Table 1:** Gene clusters significantly differing between ARDS cases and sepsis controls

Cluster ID	Genes	Cases included	Cluster *p*-value	Top pathway enriched in the cluster	Top pathway *p*-value
12	110	272	3.29 × 10^–10^	Amino acids regulate mTORC1	2.02 × 10^–12^
170	114	271	2.58 × 10^–9^	Toll-like receptor cascades	8.02 × 10^–21^
169	99	271	3.34 × 10^–9^	Transcriptional regulation of white adipocyte differentiation	6.02 × 10^–35^
186	110	272	8.81 × 10^–8^	Integrin cell surface interactions	2.17 × 10^–15^
92*	84	266	1.13 × 10^–7^	Signaling by receptor tyrosine kinases	8.62 × 10^–18^
153	88	268	1.21 × 10^–7^	Interferon signaling	9.36 × 10^–27^
196*	146	272	3.07 × 10^–7^	Sumoylation	7.12 × 10^–15^
97	91	270	4.21 × 10^–7^	DNA repair	6.50 × 10^–23^
19	80	270	4.97 × 10^–7^	Protein–protein interactions at synapses	5.48 × 10^–12^
182	102	271	6.97 × 10^–7^	mTOR signaling	1.59 × 10^–14^
165*	89	269	9.83 × 10^–7^	Integrin cell surface interactions	8.06 × 10^–13^
177*	124	271	1.03 × 10^–6^	Toll-like receptor cascades	1.42 × 10^–18^
46	88	269	1.31 × 10^–6^	mRNA splicing	9.94 × 10^–25^
204*	121	271	1.61 × 10^–6^	mRNA splicing	3.51 × 10^–32^
156*	91	268	1.72 × 10^–6^	Integrin cell surface interactions	6.30 × 10^–20^
24*	85	270	2.39 × 10^–6^	Integrin cell surface interactions	4.97 × 10^–16^
181	121	263	3.93 × 10^–6^	Krebs cycle and the respiratory electron transport	7.65 × 10^–69^
126*	93	246	4.92 × 10^–6^	Krebs cycle and the respiratory electron transport	5.81 × 10^–53^
171*	109	268	5.68 × 10^–6^	Programmed cell death	4.82 × 10^–16^

^*^Clusters not depicted in supplementary figures because they show > 20% gene-level similarity with any of the represented clusters

The most frequent pathways among the clusters (Supplementary Table 2) were *Integrin cell surface interactions* (clusters 24, 156, 165, and 186) (Supplementary Fig. 4), *TLR cascades* (clusters 170 and 177) (Supplementary Fig. 5), and the *Krebs cycle and the respiratory electron transport* (clusters 126 and 181) (Supplementary Fig. 6). Less frequently enriched pathways were *Transcriptional regulation of white adipocyte differentiation* (cluster 169) (Supplementary Fig. 7), *Interferon signaling* (cluster 153) (Supplementary Fig. 8), and *mTOR signaling* (cluster 182) (Supplementary Fig. 9). Additional information can be found in the Additional file 1: Supplementary Figs. 10–13.

We identified 208 genes (or “nodes” in the networks) exhibiting QVs in ≥ 10 cases (average of 15.6 [SD = 6.79] cases) in the 19 gene clusters (Additional file 1: Supplementary Table 3). One of those genes was *FLT1* that was linked to cluster 186 (enriched in *Integrin cell surface interactions*) (Additional file 1: Supplementary Table 4), which we identified in the cohort as the main association signal based on a previous GWAS [26]. This gene list included 25 (12.02%) that are considered causes of IEIs according to the 2024 classification update [54]. The top 10 genes accumulating QV carriers among ARDS cases were: *CFTR* (57 cases), *MPDZ* (56 cases), *SRRM2* (38 cases), *BRCA2* (33 cases), *NOTCH2* (32 cases), *PLCG2* (32 cases), *CGN* (32 cases), *PLXNA4* (32 cases), *NRP2* (31 cases), and *TJP2* (31 cases). Most of these genes were part of more than one significant cluster (*CFTR* in 13/19, *NOTCH2* and *CGN* in 4/19, *SRRM2, PLXNA4, BRCA2* and *NRP2* in 2/19). *MPDZ*, *PLCG2*, and *TJP2* were each part of one significant cluster (Additional file 1: Supplementary Table 5).

### Qualifying variant carriers and sepsis-associated ARDS risk

As a cross-verification of results, we then tested the association between carrier status for the QVs-CADD15 in the 10 gene clusters having the lowest pairwise similarity with ARDS risk using regression models. Carrier status in nine of the gene clusters was associated with increased risk of sepsis-associated ARDS (Table 2), reflecting the broad systemic response involved in ARDS development. These results were robust to model adjustments for the first two main PCs, and for relevant demographic and clinical variables (Supplementary Table 6). The models based on QVs-CADD10 or on QVs-CADD15 with AF < 0.001 provided consistent results, although showing larger effect sizes or being non-significant, reflecting the limited sample size (Supplementary Table 7). Null models with the synonymous variants for the 10 gene clusters at the same AF threshold showed no association with ARDS (Supplementary Table 8).

**Table 2:** Association of qualifying variant carriers (CADD ≥ 15) with sepsis-associated ARDS risk in the 10 significant gene clusters with the lowest pairwise similarities

Cluster ID	Top enriched pathway	Genes	Odds Ratio (95% CI)	*p*-value
12	Amino acids regulate mTORC1	110	6.57 (2.75—15.68)	2.0 × 10^–5^
170	Toll-like receptor cascades	114	3.72 (2.05—6.71)	1.0 × 10^–5^
169	Regulation of white adipocyte differentiation	99	3.05 (1.61—5.80)	6.3 × 10^–4^
186	Integrin cell surface interactions	110	6.10 (2.15—17.29)	6.6 × 10^–4^
153	Interferon signaling	88	2.61 (1.48—4.61)	9.2 × 10^–4^
97	DNA repair	91	2.78 (1.46—5.29)	1.8 × 10^–3^
19	Protein–protein interactions at synapses	80	3.88 (1.86—8.07)	2.8 × 10^–4^
182	mTOR signaling	102	2.63 (1.30—5.34)	7.2 × 10^–3^
46	mRNA splicing	88	3.42 (1.65—7.09)	9.2 × 10^–4^
181	Krebs cycle and respiratory electron transport	121	3.04 (1.75—5.28)	7.0 × 10^–5^

*CI* confidence interval

The most significant association was found for carriers of QVs in genes from cluster 170, where *TLR cascades* was the top-most enriched pathway (Odds Ratio [OR] = 3.72; 95% Confidence Interval [CI] = 2.05–6.71; *p* = 1 × 10^–5^). However, the largest effect size was found for carriers of QVs in genes from cluster 12, in which *Amino acids regulate mTORC1* was the top enriched pathway (OR = 6.57; 95% CI = 2.75–15.68; *p* = 2 × 10^–5^). A similar large effect was also detected for carriers of QVs in genes from cluster 186, where *Integrin cell surface interactions* was the most enriched pathway (OR = 6.10; 95% CI = 2.15–17.29; *p* = 6.6 × 10^–4^). Despite that, all clusters provided consistent genetic variance estimates of the QVs in the range of 7.8%−10.9%. We warn that such estimates should be taken with caution given the potential of model overfitting. Associations were not significant for models that only considered QV carriers in the TLR3- and TLR7-dependent interferon response genes underlying life-threatening COVID-19 at AF ≤ 0.01 (OR = 0.81; 95% CI = 0.54–1.22; *p* = 0.33) or at AF ≤ 0.001 (OR = 0.79; 95% CI = 0.47–1.35; *p* = 0.39).

Finally, to rule out that QVs carrier status in these gene clusters was merely reflecting higher mortality, associations between QVs carriers and 28-day survival were tested using two alternative models. Models for all but one gene cluster (cluster 182) passed the proportionality of hazards test and none of the models were significant (Table 3). This evidenced that associations of carrier status with ARDS risk were not confounded by mortality.

**Table 3:** Results of the 28-day survival analyses for the 10 significant gene clusters with the lowest pairwise similarities (QVs with CADD ≥ 15)

Cluster ID	Top enriched pathway	Genes	Model 1: sex, age, APACHE II	Model 2: sex, age, APACHE II, ARDS status
			Hazard Ratio (95% CI); *p*-value	Hazard Ratio (95% CI); *p*-value
12	Amino acids regulate mTORC1	110	1.14 (0.64—2.01); *p* = 0.645	0.98 (0.55—1.76); *p* = 0.964
170	Toll-like receptor cascades	114	1.26 (0.76—2.10); *p* = 0.359	1.14 (0.68—1.91); *p* = 0.611
169	Regulation of white adipocyte differentiation	99	0.60 (0.37—0.97); *p* = 0.038	0.54 (0.33—0.88); *p* = 0.013
186	Integrin cell surface interactions	110	1.30 (0.61—2.79); *p* = 0.487	1.14 (0.53—2.47); *p* = 0.723
153	Interferon signaling	88	0.95 (0.59—1.50); *p* = 0.825	0.89 (0.56—1.42); *p* = 0.635
97	DNA repair	91	1.07 (0.60—1.91); *p* = 0.793	0.97 (0.54—1.73); *p* = 0.924
19	Protein–protein interactions at synapses	80	3.80 (1.40—10.26); *p* = 0.008	3.44 (1.26—9.35); *p* = 0.015
182^#^	mTOR signaling	102	0.97 (0.54—1.71); *p* = 0.918	0.90 (0.51—1.60); *p* = 0.729
46	mRNA splicing	88	1.29 (0.65—2.53); *p* = 0.458	1.17 (0.59—2.32); *p* = 0.643
181	Krebs cycle and respiratory electron transport	121	1.23 (0.74—2.04); *p* = 0.420	1.10 (0.66—1.85); *p* = 0.703

*APACHE II* Acute Physiology and Chronic Health Disease Classification System II. *CI* confidence interval. Analysis performed in 665 of the patients with available 28-day mortality data. ^#^Used the weighted Cox regression model, not assuming the proportionality of hazards

## Discussion

Studies performed over the last 30 years have revealed that a fraction of severe infectious disease by a particular microorganism can be etiologically explained by human genetic and immunological determinants in a growing proportion of patients [55]. Although most of this evidence is based on familial analyses or isolated cases, cohort studies also support this idea, and the COVID-19 pandemic has accelerated the acceptance of this view by a wider audience [17]. This is consistent with the idea that there is a strong genetic influence on the risk of fatal outcomes from infections, as shown by family, twin, and adoption studies [56, 57]. Here, we show that genetic variation affecting biologically proximal gene clusters is associated ARDS risk among sepsis patients. This provides novel, actionable evidence for specific therapies targeting host dysregulation. We identified QVs with predicted impact in gene function in a cohort of unrelated sepsis patients. We used a network-based approach to identify variant enrichment in high-confidence, biologically connected gene clusters. The most salient genetic association was observed for variants in gene clusters enriched in the *TLR cascades*. We also show that there is no overlap between the genetic risk for ARDS and patient survival, likely indicating that sepsis severity and mortality may be governed by distinct genetic factors, as proposed for COVID-19 [58, 59]. To our knowledge, this is the first study reporting that rare genetic variation is associated with sepsis-associated ARDS.

Analyses accounting for aggregated variants of genes and pathways, as opposed to traditional GWAS frameworks with variant- or gene-level analyses, have shown clear power improvements. Despite the existing network data are incomplete limiting our ability to understand biological systems, these enable capturing the genetic architecture of complex traits, particularly affected by genetic heterogeneity and incomplete penetrance, and for providing biological explanations of disease risk and novel therapeutic targets [46, 60]. Similar concepts underlie genetic screenings in Mendelian diseases when prioritizing the genetic etiology from WES data based on protein interaction networks combined with phenotype information [61]. Our study demonstrates that these approaches are powerful for assessing the heterogeneous genetic risks of sepsis-associated ARDS. We have identified that aggregated gene variant effects of *TLR cascades* are strongly associated. While the way the QVs are selected influenced the effect size estimates, previous studies have found significant discrepancies of effect size estimates derived from models using predicted *vs.* biochemically ascertained loss-of-function variants [62]. Thus, we warn that the aggregated estimated effect sizes and the associated genetic variance explained by the models in this study should be interpreted with caution and require validation in independent studies.

Common genetic variation in stress and immune response-related genes, particularly those involved in pathogen recognition and signaling [17], influence individual responses to infection and organ dysfunction [63]. TLR-mediated activation is intrinsically related to immune response to pathogens. Experimental animal models of sepsis support that *TLR cascades* are determinants of survival [64]. Myd88 or Tlr4 knock-outs, as well as neutralization of TLR-4, protects mice from lethal Gram-negative sepsis [65], suggesting that excessive inflammation mediated by *TLR cascades* contributes to sepsis aggravation. This is in line with the prevailing view that sepsis and sepsis-associated organ dysfunction are due to a dysregulated immune response dependent on different factors, including innate immune activation [66]. Variants from several of the genes participating in the *TLR cascades* have been associated with differential cytokine responses to pathogen associated molecular patterns in humans [67]. Patients with inherited deficiencies in MyD88 and IRAK-4, which are crucial for canonical TLR signaling, are particularly prone to specific invasive bacterial infections. Remarkably, clinical and laboratory signs of inflammation develop slowly in these patients, even during invasive bacterial disease [68]. Common variants in *TLR1* have been linked to increased risk of ARDS, organ dysfunction, and TLR1-mediated production of inflammatory cytokines in healthy volunteers. Those variants have also been associated with sustained pro-inflammatory responses in sepsis patients [67, 69]. One of the main etiological factors revealed so far of life-threatening viral infections is the type-I interferon response genes [22, 70]. There is evidence that common and rare loss-of-function variants of genes involved in TLR-3, TLR-7, and IRF7-dependent type I interferon immunity are key components associated with severe COVID-19 [19, 71], and patients with TLR3 deficiency are particularly susceptible to herpes simplex encephalitis and prone to critical influenza pneumonia [55]. In COVID-19, rare variant aggregated effects have shown very strong (OR > 20) effect sizes in independent cohorts [22, 62]. Despite models focusing on TLR3- and TLR7-dependent interferon response genes underlying life-threatening COVID-19 did not explain ARDS risk in our study, our results support a highly heterogeneous genetic component associated with rare variants affecting genes of the *TLR cascades* and with strong aggregated effect sizes in ARDS among non-viral sepsis patients given that most infections with pathogens identified in the cohort (> 90%) were due to bacteria.

Our results also identified associations between other gene clusters and pathways that seem to play a key role in ARDS risk. However, their pathogenic links are less frequently explored in the literature and are further blurred by the pervasive genetic pleiotropy underlying complex diseases [72], the important genetic heterogeneity of ARDS, and the profound systemic nature of the underlying condition [3]. The gene cluster enriched in *Integrin cell surface interactions* is significant at pathophysiological level in the detrimental fibroproliferative response occurring in ARDS [73]. This view is supported by the shared genetic component, particularly of genes in integrin and cadherin signaling pathways, between idiopathic pulmonary fibrosis and severe COVID-19 [58]. Carriers of rare variants affecting *CFTR* gene function, who do not develop cystic fibrosis, have a higher risk of severe COVID-19 symptoms and death [74]. *CFTR* heterozygous carriers are at higher risk of respiratory infections [75, 76], and a common intronic risk variant in *CFTR* is associated with respiratory failure due to infections [77]. *CFTR* was found in 13 out of the 19 significant gene clusters revealed in our study. Interestingly, it has been recognized as a possible therapeutic target in ARDS due to severe pneumonia [78] based on findings in experimental animal models [79]. Increasing evidence shows that cell–cell signaling mechanisms play an important role during inflammation [80]. On the other hand, the acute and energetic early host defense associated with ARDS is known to lead to metabolic failure, immune cell dysfunction [81], and immunosuppression [66], where mitochondrial dysfunction is central [82]. The reasons are diverse and related to the key role of mitochondria in supporting energy-dependent processes through cellular respiration, linked to the *Krebs cycle and respiratory electron transport*, and their metabolic influence on immune response [83]. These and the other significant gene clusters revealed, which are enriched in intertwined biological processes, suggest the possibility that multiple and biologically related genetic factors are acting in combination contributing to ARDS risk.

We acknowledge several limitations in the study. First, we support the biological relevance of our findings with the published literature, but we lack data from similar sequencing studies in independent sepsis-associated ARDS patients that can validate our findings. Most published sequencing studies to date have focused on patients with COVID-19 or on kindreds with affected family members. Second, there is a limitation in how our findings generalize to other populations given that the study mainly involved individuals of European genetic ancestry. Further steps to overcome these limitations will need data from whole-exome or whole-genome sequencing studies in cohorts of patients of diverse genetic ancestries with clinically curated ARDS cases to avoid phenotypic misclassification that can dilute effect sizes as have been observed in other diseases [84]. Third, given the large heterogeneity of ARDS, we relied on a network-based approximation to identify multiple genes that are biologically connected and that could be collectively associated with the disease risk. Thus, while our analyses were unrestricted to candidate genes or inheritance models and offers an unbiased genome-wide assessment, we cannot draw conclusions at variant- or gene-level data. Connected to this, the underlying gene networks of NHC were based on incomplete protein interaction data, which could have affected our inferences. Lastly, our analyses relied on QVs that were kept in the study based on their frequency and potential protein truncation prediction. We do not provide biochemical evidence of QVs effects in gene function, which could have improved the prioritization of perturbed gene clusters and refined the most salient pathways associated with ARDS.

## Conclusions

Our findings highlight the complexity of the genetic factors involved in sepsis-associated ARDS risk. These factors affect diverse biological processes, including the *TLR cascades*, that may reconcile with its complex pathophysiology. They also support an underlying landscape of genetic heterogeneity in the spectrum of rare variants influencing disease susceptibility. It is likely that some of these genetic factors could be actionable, offering a possibility to identify therapeutic targets for further research to mitigate the risk of severe outcomes among sepsis patients.

## Supplementary Information


Additional file 1: Includes supplementary methods and references describing workflows for WES and SNP array data, supplementary tables 1-6 and figures 1-13. Supplementary Table 1 Demographics and clinical characteristics of the patients from the GEN-SEP cohort included in this study; Supplementary Table 2 Enriched significant pathways in the significant gene clusters; Supplementary Table 3 Genes exhibiting qualifying variants in at least 10 ARDS cases in the study; Supplementary Table 4 Qualifying variants found in *FLT1* in the GEN-SEP cohort patients; Supplementary Table 5 Qualifying variants in the ten most frequent genes observed in the ARDS cases. The number of carriers in ARDS cases and in sepsis controls, along with standardized identifiers, are indicated; Supplementary Table 6 Odds ratios resulting from sensitivity analysis using logistic regressions of patient-recorded variables for the ARDS risk-associated gene clusters with minimal similarity. Results from the stricter model, adjusted for sex, age, and APACHE II scores, excluding and including first 2 principal components, and from models that did not adjust for any covariate are included for reference; Supplementary Table 7 Results from sensibility analyses of QVs with AF≤ 0.01 and AF ≤ 0.001 in gene clusters with minimal similarity. The models were adjusted for sex, age and APACHE II scores; Supplementary Table 8 Results of the null model based on logistic regression analysis of rare synonymous variants in gene clusters with minimal similarity. The models were adjusted for sex, age and APACHE II scores. Supplementary Fig. 1 Plot of the first two principal components of genetic variation of GEN-SEP patients and of data from the 1000 Genomes Project from African, American, East Asian, European, and South Asian. GEN-SEP cases and controls are indicated with differentiated symbols. The percentages represent the explained variability of each axis; Supplementary Fig. 2 Schematic overview of the sequencing workflow, filtering for qualifying variant identification, and the network-based heterogeneity clustering analysis; Supplementary Fig. 3 Similarity matrix, as the inverse of the Jaccard distance, reflecting the percentage of gene-level overlap between significant gene clusters; Supplementary Fig. 4 Network representation of Cluster 186, the most significant among the significant clusters with the *Integrin cell surface interactions* as the top-most significant associated pathway; Supplementary Fig. 5 Network representation of Cluster 170, the most significant among the significant clusters with the *Toll-like receptor cascades *as the top-most significant associated pathway; Supplementary Fig. 6 Network representation of Cluster 181, the most significant among the significant clusters with the *Krebs cycle and respiratory electron transport* as the top-most significant associated pathway; Supplementary Fig. 7 Network representation of Cluster 169, the only significant cluster with *Transcriptional regulation of white adipocyte differentiation* as the top-most significant associated pathway; Supplementary Fig. 8 Network representation of Cluster 153, the most significant among the significant clusters with the *Interferon signaling* as the top-most significant associated biological pathway; Supplementary Fig. 9 Network representation of Cluster 182, the only significant cluster with *mTOR signaling* as the top-most significant associated pathway; Supplementary Fig. 10 Network representation of Cluster 12, the only significant cluster with *Amino acids regulate mTORC1* as the top-most significant associated pathway; Supplementary Fig. 11 Network representation of Cluster 19, the only significant cluster with *Protein-protein interactions at synapses* as the top-most significant associated pathway; Supplementary Fig. 12 Network representation of Cluster 46, the most significant among the significant clusters with the *mRNA splicing* as the top significant associated pathway; Supplementary Fig. 13 Network representation of Cluster 97, the most significant among the significant clusters with the *DNA repair* as the top-most significant associated pathway.


## Data Availability

Raw genotype or phenotype data cannot be made publicly available due to restrictions imposed by the ethics approval. Variant calls from whole-exome sequencing have been deposited in EGA (EGAS50000001119). Details for accessing the data can be found at https:/github.com/genomicsITER/HGinfections.
