# Supplementary material for: Rare genetic variant risks in patients with sepsis-associated acute respiratory distress syndrome
Source: Respir Res. 2026 Feb 25;27:154. doi: 10.1186/s12931-026-03588-4 (PMC13040698; doi:10.1186/s12931-026-03588-4)

**Supplementary material**

**Rare genetic variant risks in patients with sepsis-associated acute respiratory distress syndrome**

Eva Tosco-Herrera^1^, Luis A. Rubio-Rodríguez^2^, Adrián Muñoz-Barrera^2^, David Jáspez^2^, Eva Suárez-Pajes^1^, Almudena Corrales^1,3^, Aitana Alonso-González^1^, Miryam Prieto-González^4^, Aurelio Rodríguez-Pérez^5,6^, Demetrio Carriedo^7^, Jesús Blanco^3,8^, Alfonso Ambrós^9^, Leonardo Lorente^10^, María M. Martín^11^, Jordi Solé-Violán^3,12,13^, Carlos Rodríguez-Gallego^3,6,12,14^, Elena González-Higueras^15^, Elena Espinosa^16^, Arturo Muriel-Bombin^8^, David Domínguez^16^, Marina Soro^17^, Tamara Hernández-Beeftink^18,19^, José M. Añón^3,20^, Jesús Villar^3,21,22,23^, Beatriz Guillén-Guio^3,18,19^, Itahisa Marcelino-Rodríguez^24,25^, José M. Lorenzo-Salazar^2^, Rafaela González-Montelongo^2^, Carlos Flores^1,2,3,12,25^

*1 Research Unit, Hospital Universitario Nuestra Señora de Candelaria, Instituto de Investigación Sanitaria de Canarias (IISC), Santa Cruz de Tenerife, Spain.*

*2 Genomics Division, Instituto Tecnológico y de Energías Renovables (ITER), Santa Cruz de Tenerife, Spain.*

*3 CIBER de Enfermedades Respiratorias (CIBERES), Instituto de Salud Carlos III, Madrid, Spain.*

*4 Intensive Care Unit, Complejo Asistencial Universitario de Palencia, Palencia, Spain.*

*5 Department of Anesthesiology, Hospital Universitario de Gran Canaria Dr. Negrín, Las Palmas de Gran Canaria, Spain.*

*6 Department of Medical and Surgical Sciences, School of Medicine, University of Las Palmas de Gran Canaria, Las Palmas de Gran Canaria, Spain.*

*7 Intensive Care Unit, Complejo Hospitalario Universitario de León, León, Spain.*

*8 Intensive Care Unit, Hospital Universitario Rio Hortega, Valladolid, Spain.*

*9 Intensive Care Unit, Hospital General de Ciudad Real, Ciudad Real, Spain.*

*10 Intensive Care Unit, Hospital Universitario de Canarias, Santa Cruz de Tenerife, Spain.*

*11 Intensive Care Unit, Hospital Universitario Nuestra Señora de Candelaria, Santa Cruz de Tenerife, Spain.*

*12 Department of Clinical Sciences, University Fernando Pessoa Canarias, Las Palmas de Gran Canaria, Spain.*

*13 Critical Care Unit, Hospital Universitario de Gran Canaria Dr. Negrín, Las Palmas de Gran Canaria, Spain.*

*14 Department of Immunology, Hospital Universitario de Gran Canaria Dr. Negrín, Las Palmas de Gran Canaria, Spain.*

*15 Intensive Care Unit, Hospital Virgen de la Luz, Cuenca, Spain.*

*16 Department of Anesthesiology, Hospital Universitario Nuestra Señora de Candelaria, Santa Cruz de Tenerife, Spain.*

*17 Anesthesiology and Critical Care Department, Hospital IMED Valencia, Valencia, Spain.*

*18 Department of Population Health Sciences, University of Leicester, Leicester, United Kingdom.*

*19 NIHR Leicester Biomedical Research Centre, Leicester, United Kingdom.*

*20 Intensive Care Unit, Hospital Universitario La Paz, IdiPAZ, Madrid, Spain.*

*21 Research Unit, Hospital Universitario Dr. Negrín, Las Palmas de Gran Canaria, Spain.*

*22 Li Ka Shing Knowledge Institute, St. Michael’s Hospital, Toronto, Canada.*

*23 Faculty of Health Sciences, Universidad del Atlántico Norte, Las Palmas de Gran Canaria, Spain.*

*24 Public Health and Preventive Medicine Area, University of La Laguna, Santa Cruz de Tenerife, Spain.*

*25 Instituto de Tecnologías Biomédicas (ITB), University of La Laguna, Santa Cruz de Tenerife, Spain.*

Correspondence: Dr. Carlos Flores, Email: [cflores.genomica@gmail.com](mailto:cflores.genomica@gmail.com)

Supplementary Methods 4

Summary of the whole-exome sequencing data of the GEN-SEP cohort 4

SNP array data from the GEN-SEP cohort 4

Supplementary references 4

Supplementary Tables 6

Supplementary Table 1 6

Supplementary Table 2 8

Supplementary Table 3 23

Supplementary Table 4 30

Supplementary Table 5 31

Supplementary Table 6 48

Supplementary Table 7 51

Supplementary Table 8 52

SUPPLEMENTARY FIGURES 53

Supplementary Figure 1 53

Supplementary Figure 2 54

Supplementary Figure 3 55

Supplementary Figure 4 56

Supplementary Figure 5 57

Supplementary Figure 6 58

Supplementary Figure 7 59

Supplementary Figure 8 60

Supplementary Figure 9 61

Supplementary Figure 10 62

Supplementary Figure 11 63

Supplementary Figure 12 64

Supplementary Figure 13 65

# Supplementary Methods

## **Summary of the whole-exome sequencing data of the GEN-SEP cohort**

Patient samples were sequenced to a mean target coverage of 105X. An average of 123 million reads per patient were captured, where 85% of the on-target regions were covered at 30X depth, and an average of 122.7 million reads (99.6%) were aligned to the reference. In total, an average of 73,705 SNVs and 13,138 small indels per sample were called with an average variant depth of 70.3X. Based on these, the estimated average transition-to-transversion ratio was 2.35, close to the expected range for whole-exome data [1], considering that the addition of a 100 bp padding extends the captured regions into intronic sequences, thereby increasing both the total sequence coverage and total number of called variants.

## **SNP array data from the GEN-SEP cohort**

SNP genotyping of samples was completed with the Axiom® Genome-Wide Human CEU 1 Array (Thermo Fisher Scientific, Waltham, MA) at the National Genotyping Centre (CeGen) as described elsewhere [2,3]. Quality controls were performed using PLINK v1.90 and R v4.3.2 [4]. Samples with missing clinical information, genotype call rates (CR) <95%, sex mismatches between genetic inferred sex and the clinical data, samples with high degree of kinship (PIHAT>0.2), and heterozygosity outliers were excluded. Variants with minor allele frequency <1%, genotype completion rate <95%, or strongly deviating from the Hardy Weinberg equilibrium expectations (*p*<1.0x10^-6^) were excluded from the analyses. For principal components (PCs) analysis, we removed the variants located in regions known to exhibit long-range linkage disequilibrium (LD) and also pruned the variants in high LD using the function “indep-pairwise” of PLINK v1.9, setting a r^2^ of 0.15 to keep approximately 100,000 independent variants. The first three PCs were derived with PLINK and used for downstream analyses.

# Supplementary references

1. Trubetskoy V, Rodriguez A, Dave U, Campbell N, Crawford EL, Cook EH, et al. Consensus Genotyper for Exome Sequencing (CGES): improving the quality of exome variant genotypes. Bioinformatics [Internet]. 2015 [cited 2022 Mar 28];31:187–93. Available from: http://doi.org/10.1093/bioinformatics/btu591

2. Guillen-Guio B, Lorenzo-Salazar JM, Ma S-F, Hou P-C, Hernandez-Beeftink T, Corrales A, et al. Sepsis-associated acute respiratory distress syndrome in individuals of European ancestry: a genome-wide association study. Lancet Respir Med [Internet]. 2020 [cited 2020 Nov 27];8:258–66. Available from: http://doi.org/10.1016/S2213-2600(19)30368-6

3. Hernandez-Beeftink T, Guillen-Guio B, Lorenzo-Salazar JM, Corrales A, Suarez-Pajes E, Feng R, et al. A genome-wide association study of survival in patients with sepsis. Crit Care [Internet]. 2022 [cited 2024 Feb 22];26:341. Available from: http://doi.org/10.1186/s13054-022-04208-5

4. R Studio Team. RStudio: Integrated Development for R. RStudio, PBC, Boston, MA URL http://www.rstudio.com/. 2020.

# Supplementary Tables

| **Supplementary Table 1** Demographics and clinical characteristics of the patients from the GEN-SEP cohort included in this study. | | | | |
| --- | --- | --- | --- | --- |
| Variable | All (n=822) | ARDS cases (n=272) | Sepsis controls (n=550) | p-value^#^ |
| Sex, males (%) | 523 (63.63) | 187 (68.75) | 336 (61.09) | 0.032 |
| Age, mean (SD) | 63.18 (15.05) | 62.17 (13.91) | 63.68 (15.58) | 0.074 |
| BMI, mean (SD) | 27.43 (6.06) | 28.65 (6.77) | 26.79 (5.55) | 5.69x10^-3^ |
| SOFA, median (IQR) | 8 (6-11) | 9 (7-12) | 8 (6-10) | 4.52x10^-7^ |
| APACHE II, median (IQR) | 19 (15-24) | 21 (16-27) | 18 (14-23) | 9.88x10^-7^ |
| Days from diagnosis, median (IQR) | 20 (11-37) | 24 (13-49.5) | 18.5 (10-31.8) | 2.52x10^-3^ |
| ICU mortality, count (%) | 199 (24.21) | 99 (36.4) | 100 (18.18) | 1.76x10^-9^ |
| 28-day mortality, count (%) | 155 (18.86) | 71 (26.10) | 84 (15.27) | 7.79x10^-5^ |
| Source of infection, count (%) |  |  |  | 5.32x10^-9^ |
| Extrapulmonary | 486 (59.12) | 126 (46.32) | 360 (65.45) |  |
| Pulmonary | 237 (28.83) | 113 (41.54) | 124 (22.55) |  |
| Pathogen identified, count (%) |  |  |  | 0.043 |
| Gram(+) | 146 (17.76) | 51 (18.75) | 95 (17.27) |  |
| Gram(-) | 198 (24.09) | 63 (23.16) | 135 (24.54) |  |
| Gram(+) and Gram(-) | 78 (9.49) | 19 (6.99) | 59 (10.73) |  |
| Fungi | 13 (1.58) | 3 (1.10) | 10 (1.82) |  |
| Viral | 19 (2.31) | 12 (4.41) | 7 (1.27) |  |
| Polymicrobial | 61 (7.42) | 19 (6.98) | 42 (7.64) |  |
| Unidentified | 307 (37.35) | 105 (38.60) | 202 (36.73) |  |
| Patients with comorbidities*, count (%) | 382 (46.47) | 120 (44.12) | 262 (47.64) | 0.26 |
| APACHE-II: Acute Physiology and Chronic Health Disease Classification System II; BMI: Body mass index; ICU: Intensive care unit; IQR: Interquartile range; SD: Standard deviation; SOFA: Sequential Organ Failure Assessment Score; ^#^Used a chi-squared test for comparisons, except for age, BMI, SOFA, APACHE II, and days from diagnosis for which we used the Mann-Whitney U-test. *Comorbidities include: age ≥ 80 years, pregnancy, alcoholism and smoking habits, or personal history of respiratory, cardiovascular, metabolic, endocrine, oncological, hematological, autoimmune, connective tissue, renal, hepatic, neurological or infectious conditions. | | | | |

| **Supplementary Table 2**. Enriched significant pathways (p<1e-05) in the significant gene clusters. | | | | | | |
| --- | --- | --- | --- | --- | --- | --- |
| **Cluster ID** | **N Genes** | **N Pathways** | **N Cases** | **Cluster p-value** | **Top pathway (p-value)** | **Significant pathways (p-value)** |
| **Cluster_12** | 110 | 11 | 272 | 3.29e-10 | Amino acids regulate mTORC1 (2.02e-12) | Amino acids regulate mTORC1 (2.02e-12) Insulin receptor recycling (2.59e-11) Transferrin endocytosis and recycling (1.25e-10) Signaling by insulin receptor (2.62e-09) ROS and RNS production in phagocytes (1.39e-08) Autophagy (4.06e-08) Iron uptake and transport (4.35e-08) G alpha Q signalling events (4.93e-07) Cellular responses to external stimuli (3.32e-06) |
| **Cluster_170** | 114 | 29 | 271 | 2.58e-09 | Toll-like receptor cascades (8.02e-21) | Toll-like receptor cascades (8.02e-21) Nucleotide binding domain leucine rich repeat containing receptor NLR signaling pathways (1.65e-19) Innate immune system (9.79e-18) Toll-like receptor 4 cascade (2.42e-17) TNF signaling (1.84e-13) Diseases of immune system (4.90e-13) Death receptor signalling (8.21e-13) Cytokine signaling in immune system (6.16e-12) Interleukin-1 family signaling (9.72e-12) MYD88 independent TLR4 cascade (1.19e-11) TNFR1 induced NFKappaB signaling pathway (1.39e-10) Interleukin-1 signaling (3.95e-10) NOD1 2 signaling pathway (7.29e-10) Toll-like receptor TLR1 TLR2 cascade (3.29e-09) Signaling by interleukins (4.40e-09) Inflammasomes (6.37e-09) Regulation of TNFR1 signaling (9.56e-09) Toll-like receptor 9 cascade (3.91e-08) Programmed cell death (6.74e-07) Purinergic signaling in leishmaniasis infection (7.41e-07) Caspase activation via extrinsic apoptotic signalling pathway (1.26e-06) Caspase activation via death receptors in the presence of ligand (2.082e-06) RIP mediated NFkB activation via ZBP1 (2.92e-06) RIPK1 mediated regulated necrosis (7.31e-06) ZBP1 DAI mediated induction of type I IFNs (9.67e-06) |
| **Cluster_169** | 99 | 54 | 271 | 3.34e-09 | Transcriptional regulation of white adipocyte differentiation (6.02e-35) | Transcriptional regulation of white adipocyte differentiation (6.02e-35) Regulation of lipid metabolism by PPARalpha (1.23e-32) Sumoylation (1.03e-16) Nuclear pore complex NPC disassembly (3.04e-15) Regulation of HSF1 mediated heat shock response (4.50e-15) Regulation of glucokinase by glucokinase regulatory protein (3.41e-14) Export of viral ribonucleoproteins from nucleus (4.73e-14) Nuclear import of REV protein (6.49e-14) RNA polymerase II transcription (7.32e-14) Sumoylation of sumoylation proteins (8.84e-14) Cellular response to heat stress (1.01e-13) Transport of the SLBP dependant mature mRNA (1.19e-13) Interactions of VPR with host cellular proteins (1.60e-13) Interactions of REV with host cellular proteins (1.60e-13) Sumoylation of ubiquitinylation proteins (2.83e-13) Nuclear envelope breakdown (2.84e-13) NS1 mediated effects on host pathways (4.88e-13) Transport of mature mRNAs derived from intronless transcripts (8.21e-13) Viral messenger RNA synthesis (1.06e-12) Sumoylation of DNA replication proteins (1.72e-12) Sumoylation of RNA binding proteins (2.19e-12) Metabolism of lipids (8.49e-12) Sumoylation of chromatin organization proteins (9.79e-12) snRNP assembly (1.025e-11) tRNA processing in the nucleus (2.77e-11) Transport of mature transcript to cytoplasm (7.71e-11) Cell cycle mitotic (1.71e-10) Glycolysis (2.65e-10) Postmitotic nuclear pore complex NPC reformation (4.35e-10) Sumoylation of DNA damage response and repair proteins (5.69e-10) Antiviral mechanism by IFN stimulated genes (1.17e-09) Glucose metabolism (4.38e-09) Nuclear receptor transcription pathway (5.75e-09) HIV life cycle (9.36e-09) SLC transporter disorders (1.02e-08) Cell cycle (1.14e-08) Host interactions of HIV factors (1.89e-08) Transcriptional regulation by small RNAs (2.49e-08) HCMV early events (3.59e-08) tRNA processing (3.79e-08) Mitotic prophase (5.58e-08) HCMV late events (6.29e-08) Activation of the pre replicative complex (7.18e-08) Developmental biology (1.20e-07) Nuclear envelope NE reassembly (1.85e-07) HCMV infection (2.60e-07) Gene silencing by RNA (4.62e-07) Disorders of transmembrane transporters (7.21e-07) Sumoylation of intracellular receptors (1.07e-06) Mitotic G1 phase and G1 s transition (1.11e-06) Influenza infection (1.88e-06) Cellular responses to external stimuli (1.93e-06) HIV infection (2.71e-06) Cell cycle checkpoints (6.00e-06) |
| **Cluster_186** | 110 | 9 | 272 | 8.81e-08 | Integrin cell surface interactions (2.17e-15) | Integrin cell surface interactions (2.17e-15) Extracellular matrix organization (1.46e-08) Signaling by receptor tyrosine kinases (2.66e-06) Cellular response to heat stress (9.05e-06) |
| **Cluster_92** | 84 | 16 | 266 | 1.13e-07 | Signaling by receptor tyrosine kinases (8.622e-18) | Signaling by receptor tyrosine kinases (8.62e-18) Endosomal sorting complex required for transport (5.93e-12) Integrin cell surface interactions (2.00e-10) Clathrin mediated endocytosis (4.15e-10) Membrane trafficking (1.34e-08) Vesicle mediated transport (3.61e-08) Cargo recognition for clathrin mediated endocytosis (7.21e-07) Signaling by VEGF (7.92e-07) Listeria monocytogenes entry into host cells (8.55e-07) Negative regulation of MET activity (1.13e-06) Budding and maturation of HIV virion (6.19e-06) Extracellular matrix organization (6.77e-06) |
| **Cluster_153** | 88 | 52 | 268 | 1.21e-07 | Interferon signaling (9.36e-27) | Interferon signaling (9.36e-27) NS1 mediated effects on host pathways (1.52e-25) Interactions of REV with host cellular proteins (1.85e-24) Antiviral mechanism by IFN stimulated genes (1.19e-23) Nuclear import of REV protein (3.25e-23) Transport of the SLBP dependant mature mRNA (7.29e-23) Interactions of VPR with host cellular proteins (1.08e-22) Sumoylation of ubiquitinylation proteins (2.29e-22) Regulation of HSF1 mediated heat shock response (4.52e-22) snRNP assembly (6.32e-22) Regulation of glucokinase by glucokinase regulatory protein (8.49e-22) Transport of mature mRNAs derived from intronless transcripts (9.51e-22) Export of viral ribonucleoproteins from nucleus (1.28e-21) Sumoylation of DNA replication proteins (2.57e-21) Sumoylation of sumoylation proteins (2.82e-21) Nuclear pore complex NPC disassembly (4.13e-21) Sumoylation (1.23e-20) Cellular response to heat stress (2.05e-20) Cytokine signaling in immune system (2.93e-20) Sumoylation of chromatin organization proteins (5.19e-20) Viral messenger RNA synthesis (6.55e-20) Sumoylation of RNA binding proteins (1.66e-19) Transport of mature transcript to cytoplasm (8.27e-19) Nuclear envelope breakdown (9.15e-19) tRNA processing in the nucleus (4.29e-18) Sumoylation of DNA damage response and repair proteins (6.67e-18) Postmitotic nuclear pore complex NPC reformation (2.13e-17) Host interactions of HIV factors (6.28e-17) Glycolysis (7.91e-17) HIV life cycle (8.96e-16) Influenza infection (1.43e-15) Glucose metabolism (3.05e-15) SLC transporter disorders (9.17e-15) Transcriptional regulation by small RNAs (2.96e-14) tRNA processing (5.16e-14) HCMV early events (7.22e-14) HCMV late events (1.01e-13) HIV infection (1.42e-13) Nuclear envelope NE reassembly (1.54e-13) HCMV infection (9.55e-13) Gene silencing by RNA (1.40e-12) Mitotic prophase (2.41e-12) Processing of capped intron containing pre mRNA (4.89e-11) Disorders of transmembrane transporters (5.67e-11) Cellular responses to external stimuli (4.35e-10) Post translational protein modification (2.56e-09) Mitotic metaphase and anaphase (5.02e-08) M phase (5.12e-08) Metabolism of carbohydrates (1.22e-07) Cell cycle mitotic (1.25e-06) Regulation of IFNa signaling (5.52e-06) |
| **Cluster_196** | 146 | 52 | 272 | 3.07e-07 | Sumoylation (7.12e-15) | Sumoylation (7.12e-15) Transport of the SLBP dependant mature mRNA (2.97e-14) Nuclear pore complex NPC disassembly (2.97e-14) Regulation of glucokinase by glucokinase regulatory protein (2.14e-13) Regulation of HSF1 mediated heat shock response (2.22e-13) Transport of mature mRNAs derived from intronless transcripts (2.58e-13) Export of viral ribonucleoproteins from nucleus (3.01e-13) Nuclear import of REV protein (4.21e-13) Sumoylation of sumoylation proteins (5.84e-13) Death receptor signalling (5.96e-13) Interactions of VPR with host cellular proteins (1.09e-12) Interactions of REV with host cellular proteins (1.09e-12) RIPK1 mediated regulated necrosis (1.88e-12) Sumoylation of ubiquitinylation proteins (1.99e-12) Nuclear envelope breakdown (3.48e-12) NS1 mediated effects on host pathways (3.54e-12) Transport of mature transcript to cytoplasm (5.33e-12) Cellular response to heat stress (5.53e-12) Viral messenger RNA synthesis (8.00e-12) Sumoylation of DNA replication proteins (1.34e-11) Sumoylation of RNA binding proteins (1.72e-11) snRNP assembly (8.77e-11) Sumoylation of chromatin organization proteins (1.42e-10) tRNA processing in the nucleus (2.50e-10) Programmed cell death (4.89e-10) Regulation by c-FLIP (1.09e-09) Glycolysis (2.69e-09) TNF signaling(2.88e-09) HIV life cycle (3.09e-09) Sumoylation of DNA damage response and repair proteins (6.01e-09) Cytokine signaling in immune system (6.02e-09) Caspase activation via death receptors in the presence of ligand (1.26e-08) Antiviral mechanism by IFN stimulated genes (1.28e-08) Caspase activation via extrinsic apoptotic signalling pathway (1.59e-08) Postmitotic nuclear pore complex NPC reformation (2.16e-08) Host interactions of HIV factors (3.74e-08) Glucose metabolism (5.10e-08) HCMV early events (7.62e-08) SLC transporter disorders (1.23e-07) Transcriptional regulation by small RNAs (3.13e-07) Trail signaling (3.30e-07) Nuclear receptor transcription pathway (3.96e-07) tRNA processing (4.87e-07) HCMV infection (6.78e-07) HCMV late events (8.25e-07) TNFR1 induced NFKappaB signaling pathway (1.13e-06) Mitotic prophase (1.15e-06) HIV infection (2.19e-06) RNA polymerase II transcription (2.57e-06) Gene silencing by RNA (6.55e-06) |
| **Cluster_97** | 91 | 28 | 270 | 4.21e-07 | DNA repair (6.50e-23) | DNA repair (6.50e-23) Transcription of the HIV genome (1.20e-22) Transcriptional regulation by TP53 (1.72e-20) RNA polymerase II transcription (2.80e-19) HIV life cycle (8.95e-17) HIV transcription initiation (5.24e-16) TP53 regulates transcription of DNA repair genes (3.36e-14) Chromatin modifying enzymes (3.39e-14) HIV infection (2.96e-13) Formation of RNA pol II elongation complex (4.55e-13) Nucleotide excision repair (1.75e-12) Regulation of TP53 activity through phosphorylation (3.25e-12) Global genome nucleotide excision repair GG-NER (2.30e-11) RUNX1 interacts with co-factors whose precise effect on RUNX1 targets is not known (7.52e-11) Homologous DNA pairing and strand exchange (2.46e-10) Regulation of TP53 activity (3.66e-10) HIV transcription elongation (5.81e-10) Resolution of D-loop structures through synthesis dependent strand annealing (5.51e-09) HDR through homologous recombination (2.15e-08) Resolution of D-loop structures (4.19e-08) RMTs methylate histone arginines (1.07e-07) Homology directed repair (1.57e-07) Formation of incision complex in GG-NER (2.59e-07) DNA double strand break repair (1.32e-06) HDR through single strand annealing (2.29e-06) HATs acetylate histones (2.79e-06) |
| **Cluster_19** | 80 | 9 | 270 | 4.97e-07 | Protein protein interactions at synapses (5.48e-12) | Protein protein interactions at synapses (5.48e-12)  Neurexins and neuroligins (2.89e-11)  Neuronal system (6.27e-11)  Neurotransmitter receptors and postsynaptic signal transmission (2.07e-08)  YAP1 and WWTR1 TAZ stimulated gene expression (1.19e-07)  Activation of NMDA receptors and postsynaptic events (1.42e-07)  RUNX3 regulates YAP1 mediated transcription (3.56e-07)  Transmission across chemical synapses (7.66e-07)  Long term potentiation (1.36e-06) |
| **Cluster_182** | 102 | 13 | 271 | 6.97e-07 | mTOR signalling (1.59e-14) | mTOR signalling(1.59e-14) mTORC1 mediated signalling (8.33e-14) Eukaryotic translation initiation (2.26e-12) Autophagy (1.01e-09) Transcriptional regulation by TP53 (1.19e-08) Activation of the mRNA upon binding of the CAP binding complex and EIFs and subsequent binding to 43S (2.63e-08) Energy dependent regulation of mTOR by IKB1 AMPK (3.54e-08) DNA double strand break repair (5.27e-07) Homologous DNA pairing and strand exchange (6.03e-07) Homology directed repair (6.75e-07) DNA repair (1.09e-06) Translation (1.49e-06) |
| **Cluster_165** | 89 | 9 | 269 | 9.83e-07 | Integrin cell surface interactions (8.06e-13) | Integrin cell surface interactions (8.06e-13) Extracellular matrix organization (2.381e-09) Signaling by NOTCH1 (4.43e-08) Signaling by NOTCH1 PEST domain mutants in cancer (1.01e-07) NOTCH-HLH transcription pathway (2.84e-07) Cellular response to heat stress (9.28e-07) Regulation of HSF1 mediated heat shock response (2.18e-06) |
| **Cluster_177** | 124 | 41 | 271 | 1.03e-06 | Toll-like receptor cascades (1.42e-18) | Toll-like receptor cascades (1.42e-18) Toll-like receptor 4 cascade (1.65e-16) Cellular response to heat stress (2.93e-13) Sumoylation (3.98e-11) MYD88 independent TLR4 cascade (4.57e-11) Regulation of hsf1 mediated heat shock response (8.02e-11) Autophagy (1.93e-09) Toll-like receptor 9 cascade (8.16e-09) TNF signaling (9.57e-09) TNFR1 induced nfkappab signaling pathway (1.01e-08) Cytokine signaling in immune system (1.61e-08) Regulation of glucokinase by glucokinase regulatory protein (1.72e-08) Export of viral ribonucleoproteins from nucleus (2.22e-08) Nuclear import of REV protein (2.85e-08) Sumoylation of sumoylation proteins (3.63e-08) Transport of the SLBP dependant mature mRNA (4.59e-08) Nuclear pore complex NPC disassembly (4.59e-08) Diseases of immune system (5.08e-08) Interactions of VPRr with host cellular proteins (5.79e-08) Interactions of REV with host cellular proteins (5.79e-08) Sumoylation of ubiquitinylation proteins (9.03e-08) Caspase activation via death receptors in the presence of ligand (1.08e-07) NS1 mediated effects on host pathways (1.38e-07) Toll-like receptor TLR1 TLR2 cascade (1.44e-07) Transport of mature mrnas derived from intronless transcripts (2.07e-07) Viral messenger RNA synthesis (2.52e-07) Sumoylation of DNA replication proteins (3.68e-07) Sumoylation of RNA binding proteins (4.43e-07) Death receptor signalling (9.94e-07) IKK complex recruitment mediated by RIP1 (1.09e-06) Sumoylation of chromatin organization proteins (1.10e-06) Nuclear envelope breakdown (1.24e-06) snRNP assembly (1.46e-06) Cellular responses to external stimuli (1.82e-06) Caspase activation via extrinsic apoptotic signalling pathway (2.46e-06) tRNA processing in the nucleus (3.15e-06) Postmitotic nuclear pore complex NPC reformation (3.16e-06) TRAF6 mediated induction of TAK1 complex within TLR4 complex (3.74e-06) Activation of IRF3 IRF7 mediated by TBK1 IKK epsilon (5.24e-06) Mitochondrial protein import (7.28e-06) |
| **Cluster_46** | 88 | 6 | 269 | 1.31e-06 | mRNA splicing (9.94e-25) | mRNA splicing (9.94e-25) Processing of capped intron containing pre-mRNA (5.76e-22) Metabolism of RNA (6.40e-11) Signaling by NOTCH3 (5.33e-07) |
| **Cluster_204** | 121 | 9 | 271 | 1.61e-06 | mRNA splicing (3.51e-32) | mRNA splicing (3.51e-32) Processing of capped intron containing pre mRNA (1.31e-28) Metabolism of RNA (1.11e-19) Signaling by NOTCH1 (8.19e-08) signaling by NOTCH1 PEST domain mutants in cancer (1.23e-07) signaling by NOTCH3 (4.98e-07) NOTCH-HLH transcription pathway (3.32e-06) |
| **Cluster_156** | 91 | 11 | 268 | 1.72e-06 | Integrin cell surface interactions (6.30e-20) | Integrin cell surface interactions (6.30e-20) Extracellular matrix organization (1.81e-14) Hemostasis (2.22e-06) Dissolution of fibrin clot (6.83e-06) Cell surface interactions at the vascular wall (7.84e-06) |
| **Cluster_24** | 85 | 13 | 270 | 2.39e-06 | Integrin cell surface interactions (4.97e-16) | Integrin cell surface interactions (4.97e-16) Signaling by receptor tyrosine kinases (2.04e-14) Signaling by VEGF (3.31e-13) Extracellular matrix organization (9.90e-10) Pentose phosphate pathway (1.84e-07) |
| **Cluster_181** | 121 | 15 | 263 | 3.93e-06 | Krebs cycle and respiratory electron transport (7.65e-69) | Krebs cycle and respiratory electron transport (7.65e-69) Respiratory electron transport (4.24e-59) Respiratory electron transport ATP synthesis by chemiosmotic coupling and heat production by uncoupling proteins (1.90e-55) Complex I biogenesis (5.78e-45) Citric acid cycle TCA cycle (2.53e-16) Pyruvate metabolism and citric acid TCA cycle (3.24e-13) Mitochondrial protein import (5.74e-06) Glyoxylate metabolism and glycine degradation (6.57e-06) |
| **Cluster_126** | 93 | 15 | 246 | 4.92e-06 | Krebs cycle and respiratory electron transport (5.81e-53) | Krebs cycle and respiratory electron transport (5.81e-53) Respiratory electron transport (1.14e-47) Respiratory electron transport ATP synthesis by chemiosmotic coupling and heat production by uncoupling proteins (1.13e-44) Mitochondrial translation (4.31e-36) Complex I biogenesis (3.49e-33) Translation (9.63e-23) Citric acid cycle TCA cycle (9.05e-13) Pyruvate metabolism and citric acid TCA cycle (4.08e-09) |
| **Cluster_171** | 109 | 22 | 268 | 5.68e-06 | Programmed cell death (4.82e-16) | Programmed cell death (4.82e-16) Autophagy (4.26e-15) Intrinsic pathway for apoptosis (4.06e-14) mTOR signalling (1.18e-09) mTORC1 mediated signalling (1.16e-08) Energy dependent regulation of mTOR by IKB1 AMPK (6.45e-08) BH3 only proteins associate with and inactivate anti apoptotic BCL2 members (7.47e-08) Transcriptional regulation by TP53 (3.26e-07) Amino acids regulate mTORC1 (4.82e-07) Caspase activation via extrinsic apoptotic signalling pathway (8.81e-07) Constitutive signaling by AKT1 E17K in cancer (8.81e-07) Caspase activation via death receptors in the presence of ligand (1.52e-06) TP53 regulates transcription of cell death genes (1.57e-06) Intra Golgi traffic (1.57e-06) RIPK1 mediated regulated necrosis (5.35e-06) Nucleotide binding domain leucine rich repeat containing receptor NLR signaling pathways (8.98e-06) Regulation by c-FLIP (9.37e-06) |

| **Supplementary Table 3.** Genes exhibiting qualifying variants in at least 10 ARDS cases in the study. | | | |
| --- | --- | --- | --- |
| **Gene name** | **NCBI gene ID** | **HGNC ID** | **Case count** |
| ***CFTR*** | 1080 | 1884 | 57 |
| ***MPDZ*** | 8777 | 7208 | 56 |
| ***SRRM2*** | 23524 | 16639 | 38 |
| ***BRCA2*** | 675 | 1101 | 33 |
| ***CGN*** | 57530 | 17429 | 32 |
| ***NOTCH2*** | 4853 | 7882 | 32 |
| ***PLCG2*** | 5336 | 9066 | 32 |
| ***PLXNA4*** | 91584 | 9102 | 32 |
| ***NRP2*** | 8828 | 8005 | 31 |
| ***TJP2*** | 9414 | 11828 | 31 |
| ***PTPN13*** | 5783 | 9646 | 30 |
| ***RELN*** | 5649 | 9957 | 28 |
| ***ATR*** | 545 | 882 | 27 |
| ***RANBP2*** | 5903 | 9848 | 27 |
| ***SPEN*** | 23013 | 17575 | 26 |
| ***WRN*** | 7486 | 12791 | 25 |
| ***EPS8*** | 2059 | 3420 | 24 |
| ***FAN1*** | 22909 | 29170 | 24 |
| ***GRIN2A*** | 2903 | 4585 | 23 |
| ***NUP133*** | 55746 | 18016 | 23 |
| ***TLR1*** | 7096 | 11847 | 23 |
| ***ACE*** | 1636 | 2707 | 22 |
| ***ADCY9*** | 115 | 240 | 22 |
| ***MED13*** | 9969 | 22474 | 22 |
| ***NOTCH3*** | 4854 | 7883 | 22 |
| ***PLG*** | 5340 | 9071 | 22 |
| ***SHANK2*** | 22941 | 14295 | 22 |
| ***ERBB2IP*** | 55914 | 15842 | 21 |
| ***IDH3A*** | 3419 | 5384 | 21 |
| ***SEC16A*** | 9919 | 29006 | 21 |
| ***TRAP1*** | 10131 | 16264 | 21 |
| ***DOCK1*** | 1793 | 2987 | 20 |
| ***ITGA4*** | 3676 | 6140 | 20 |
| ***KIAA0226*** | 9711 | 28991 | 20 |
| ***RAD54B*** | 25788 | 17228 | 20 |
| ***SDHA*** | 6389 | 10680 | 20 |
| ***TPR*** | 7175 | 12017 | 20 |
| ***DSG2*** | 1829 | 3049 | 19 |
| ***FOXM1*** | 2305 | 3818 | 19 |
| ***MRPS9*** | 64965 | 14501 | 19 |
| ***PTPN23*** | 25930 | 14406 | 19 |
| ***UVRAG*** | 7405 | 12640 | 19 |
| ***ZBP1*** | 81030 | 16176 | 19 |
| ***APC*** | 324 | 583 | 18 |
| ***CNTN6*** | 27255 | 2176 | 18 |
| ***OGDHL*** | 55753 | 25590 | 18 |
| ***SORBS1*** | 10580 | 14565 | 18 |
| ***TCIRG1*** | 10312 | 11647 | 18 |
| ***TOP3A*** | 7156 | 11992 | 18 |
| ***TP53BP1*** | 7158 | 11999 | 18 |
| ***WDHD1*** | 11169 | 23170 | 18 |
| ***ARID1A*** | 8289 | 11110 | 17 |
| ***LRP1*** | 4035 | 6692 | 17 |
| ***MLLT4*** | 4301 | 7137 | 17 |
| ***MRPL28*** | 10573 | 14484 | 17 |
| ***MYH9*** | 4627 | 7579 | 17 |
| ***NBEA*** | 26960 | 7648 | 17 |
| ***NCOA3*** | 8202 | 7670 | 17 |
| ***NOD2*** | 64127 | 5331 | 17 |
| ***PLEKHA7*** | 144100 | 27049 | 17 |
| ***RB1CC1*** | 9821 | 15574 | 17 |
| ***RRP8*** | 23378 | 29030 | 17 |
| ***SIRT3*** | 23410 | 14931 | 17 |
| ***TYK2*** | 7297 | 12440 | 17 |
| ***AFF1*** | 4299 | 7135 | 16 |
| ***APAF1*** | 317 | 576 | 16 |
| ***CD96*** | 10225 | 16892 | 16 |
| ***FANCA*** | 2175 | 3582 | 16 |
| ***FASN*** | 2194 | 3594 | 16 |
| ***FN1*** | 2335 | 3778 | 16 |
| ***GMNN*** | 51053 | 17493 | 16 |
| ***IGF1R*** | 3480 | 5465 | 16 |
| ***MRPL19*** | 9801 | 14052 | 16 |
| ***RAPGEF1*** | 2889 | 4568 | 16 |
| ***VLDLR*** | 7436 | 12698 | 16 |
| ***CASP5*** | 838 | 1506 | 15 |
| ***DEPDC5*** | 9681 | 18423 | 15 |
| ***ITGB7*** | 3695 | 6162 | 15 |
| ***MFN1*** | 55669 | 18262 | 15 |
| ***MRPL46*** | 26589 | 1192 | 15 |
| ***NDUFS1*** | 4719 | 7707 | 15 |
| ***NRBF2*** | 29982 | 19692 | 15 |
| ***NSMAF*** | 8439 | 8017 | 15 |
| ***NUP62*** | 23636 | 8066 | 15 |
| ***NUP98*** | 4928 | 8068 | 15 |
| ***PVRL2*** | 5819 | 9707 | 15 |
| ***TLR5*** | 7100 | 11851 | 15 |
| ***TNFRSF13B*** | 23495 | 18153 | 15 |
| ***ABL1*** | 25 | 76 | 14 |
| ***CFHR5*** | 81494 | 24668 | 14 |
| ***FCHO2*** | 115548 | 25180 | 14 |
| ***IFNGR2*** | 3460 | 5440 | 14 |
| ***IKBKE*** | 9641 | 14552 | 14 |
| ***INO80C*** | 125476 | 26994 | 14 |
| ***ITGB2*** | 3689 | 6155 | 14 |
| ***MCM5*** | 4174 | 6948 | 14 |
| ***MRPL12*** | 6182 | 10378 | 14 |
| ***NDUFV3*** | 4731 | 7719 | 14 |
| ***NEDD4*** | 4734 | 7727 | 14 |
| ***NRXN3*** | 9369 | 8010 | 14 |
| ***NUP188*** | 23511 | 17859 | 14 |
| ***NUP214*** | 8021 | 8064 | 14 |
| ***PALB2*** | 79728 | 26144 | 14 |
| ***PARK2*** | 5071 | 8607 | 14 |
| ***PRPF18*** | 8559 | 17351 | 14 |
| ***PTCD3*** | 55037 | 24717 | 14 |
| ***RAD51C*** | 5889 | 9820 | 14 |
| ***SLC9A3R2*** | 9351 | 11076 | 14 |
| ***SNRNP200*** | 23020 | 30859 | 14 |
| ***SQSTM1*** | 8878 | 11280 | 14 |
| ***ZEB1*** | 6935 | 11642 | 14 |
| ***CDC40*** | 51362 | 17350 | 13 |
| ***CTNND1*** | 1500 | 2515 | 13 |
| ***ERCC4*** | 2072 | 3436 | 13 |
| ***ERCC5*** | 2073 | 3437 | 13 |
| ***FOXO3*** | 2309 | 3821 | 13 |
| ***ITGA9*** | 3680 | 6145 | 13 |
| ***MAML1*** | 9794 | 13632 | 13 |
| ***MED12L*** | 116931 | 16050 | 13 |
| ***NFRKB*** | 4798 | 7802 | 13 |
| ***NLRP1*** | 22861 | 14374 | 13 |
| ***RTN4*** | 57142 | 14085 | 13 |
| ***SLC26A6*** | 65010 | 14472 | 13 |
| ***TAF1L*** | 138474 | 18056 | 13 |
| ***TANK*** | 10010 | 11562 | 13 |
| ***THOC5*** | 8563 | 19074 | 13 |
| ***TTI2*** | 80185 | 26262 | 13 |
| ***UQCRC2*** | 7385 | 12586 | 13 |
| ***BRD9*** | 65980 | 25818 | 12 |
| ***CARD9*** | 64170 | 16391 | 12 |
| ***COG8*** | 84342 | 18623 | 12 |
| ***DAP3*** | 7818 | 2673 | 12 |
| ***DHX38*** | 9785 | 17211 | 12 |
| ***EIF3A*** | 8661 | 3271 | 12 |
| ***EPOR*** | 2057 | 3416 | 12 |
| ***ERCC3*** | 2071 | 3435 | 12 |
| ***ICAM1*** | 3383 | 5344 | 12 |
| ***KIDINS220*** | 57498 | 29508 | 12 |
| ***KPNA2*** | 3838 | 6395 | 12 |
| ***LATS1*** | 9113 | 6514 | 12 |
| ***NUP205*** | 23165 | 18658 | 12 |
| ***PSTPIP1*** | 9051 | 9580 | 12 |
| ***SMG7*** | 9887 | 16792 | 12 |
| ***SMG8*** | 55181 | 25551 | 12 |
| ***TAF2*** | 6873 | 11536 | 12 |
| ***TBX5*** | 6910 | 11604 | 12 |
| ***TKT*** | 7086 | 11834 | 12 |
| ***TTI1*** | 9675 | 29029 | 12 |
| ***BCAR3*** | 8412 | 973 | 11 |
| ***BLM*** | 641 | 1058 | 11 |
| ***CCT8*** | 10694 | 1623 | 11 |
| ***EIF4G1*** | 1981 | 3296 | 11 |
| ***EPB41L1*** | 2036 | 3378 | 11 |
| ***EPO*** | 2056 | 3415 | 11 |
| ***FLT1*** | 2321 | 3763 | 11 |
| ***HDAC4*** | 9759 | 14063 | 11 |
| ***HIP1R*** | 9026 | 18415 | 11 |
| ***IRAK3*** | 11213 | 17020 | 11 |
| ***KDR*** | 3791 | 6307 | 11 |
| ***MCM7*** | 4176 | 6950 | 11 |
| ***MED13L*** | 23389 | 22962 | 11 |
| ***MIEF1*** | 54471 | 25979 | 11 |
| ***NRXN2*** | 9379 | 8009 | 11 |
| ***OXSM*** | 54995 | 26063 | 11 |
| ***PFKL*** | 5211 | 8876 | 11 |
| ***PIK3R4*** | 30849 | 8982 | 11 |
| ***SDHD*** | 6392 | 10683 | 11 |
| ***SELP*** | 6403 | 10721 | 11 |
| ***SYF2*** | 25949 | 19824 | 11 |
| ***AIM2*** | 9447 | 357 | 10 |
| ***ARID2*** | 196528 | 18037 | 10 |
| ***CARD11*** | 84433 | 16393 | 10 |
| ***CDT1*** | 81620 | 24576 | 10 |
| ***CLOCK*** | 9575 | 2082 | 10 |
| ***COG4*** | 25839 | 18620 | 10 |
| ***CRB1*** | 23418 | 2343 | 10 |
| ***DLG1*** | 1739 | 2900 | 10 |
| ***ELMO3*** | 79767 | 17289 | 10 |
| ***EPAS1*** | 2034 | 3374 | 10 |
| ***FANCC*** | 2176 | 3584 | 10 |
| ***FBLN1*** | 2192 | 3600 | 10 |
| ***GAPDH*** | 2597 | 4141 | 10 |
| ***HIF1A*** | 3091 | 4910 | 10 |
| ***IL12RB2*** | 3595 | 5972 | 10 |
| ***ITGA5*** | 3678 | 6141 | 10 |
| ***MET*** | 4233 | 7029 | 10 |
| ***MKNK1*** | 8569 | 7110 | 10 |
| ***MRPL52*** | 122704 | 16655 | 10 |
| ***NCAM1*** | 4684 | 7656 | 10 |
| ***PELP1*** | 27043 | 30134 | 10 |
| ***PLCB2*** | 5330 | 9055 | 10 |
| ***PLXNC1*** | 10154 | 9106 | 10 |
| ***RNF31*** | 55072 | 16031 | 10 |
| ***SEC24A*** | 10802 | 10703 | 10 |
| ***SH2D2A*** | 9047 | 10821 | 10 |
| ***SMARCA2*** | 6595 | 11098 | 10 |
| ***SMG1*** | 23049 | 30045 | 10 |
| ***SNCAIP*** | 9627 | 11139 | 10 |
| ***TAF1*** | 6872 | 11535 | 10 |
| ***TAX1BP1*** | 8887 | 11575 | 10 |
| ***TEAD2*** | 8463 | 11715 | 10 |
| ***TGM2*** | 7052 | 11778 | 10 |
| ***TLN1*** | 7094 | 11845 | 10 |
| ***TLR4*** | 7099 | 11850 | 10 |
| ***USP28*** | 57646 | 12625 | 10 |
| ***VPS45*** | 11311 | 14579 | 10 |
| ***WDR59*** | 79726 | 25706 | 10 |
| ***YBX3*** | 8531 | 2428 | 10 |

| **Supplementary Table 4**. Qualifying variants found in *FLT1* in the GEN-SEP cohort patients. | | | | | | |  |
| --- | --- | --- | --- | --- | --- | --- | --- |
| **Coordinate** | **rsID** | **Gene** | **Cases**  **(N carriers)** | **Controls**  **(N carriers)** | **HGVSc** | **HGVSp** | |
| chr13:28885760:G:A | rs140861115 | ***FLT1*** | 2 | 1 | ENST00000282397.4:c.3602C>T | ENSP00000282397.4:p.Pro1201Leu | |
| chr13:28964035:T:C | rs142425372 | ***FLT1*** | 1 | 1 | ENST00000282397.4:c.1867A>G | ENSP00000282397.4:p.Ile623Val | |
| chr13:28971113:T:C | rs148479824 | ***FLT1*** | 5 | 5 | ENST00000282397.4:c.1644A>G | ENSP00000282397.4:p.Ile548Met | |
| chr13:28979966:C:T | rs61763181 | ***FLT1*** | 1 | 0 | ENST00000282397.4:c.1502G>A | ENSP00000282397.4:p.Arg501Lys | |
| chr13:29001303:C:T | rs201407326 | ***FLT1*** | 1 | 0 | ENST00000282397.4:c.1429G>A | ENSP00000282397.4:p.Glu477Lys | |
| chr13:29004277:C:T | rs370686484 | ***FLT1*** | 1 | 0 | ENST00000282397.4:c.1016G>A | ENSP00000282397.4:p.Arg339Gln | |
| chr13:28886135:G:T | rs145693340 | ***FLT1*** | 0 | 1 | ENST00000282397.4:c.3487C>A | ENSP00000282397.4:p.Gln1163Lys | |
| chr13:28893614:T:C | rs895427992 | ***FLT1*** | 0 | 1 | ENST00000282397.4:c.3232A>G | ENSP00000282397.4:p.Thr1078Ala | |
| chr13:29004232:C:T | rs763071731 | ***FLT1*** | 0 | 1 | ENST00000282397.4:c.1061G>A | ENSP00000282397.4:p.Arg354Gln | |
| chr13:29004233:G:A | rs778277302 | ***FLT1*** | 0 | 1 | ENST00000282397.4:c.1060C>T | ENSP00000282397.4:p.Arg354Trp | |
|  | | | | | | | |

| **Supplementary Table 5**. Qualifying variants in the ten most frequent genes observed in the ARDS cases. The number of carriers in ARDS cases and in sepsis controls, along with standardized identifiers, are indicated. | | | | | | |
| --- | --- | --- | --- | --- | --- | --- |
| **Coordinate** | **rsID** | **Gene** | **Cases**  **(N carriers)** | **Controls**  **(N carriers)** | **HGVSc** | **HGVSp** |
| chr7:117232223:C:T | rs1800100 | ***CFTR*** | 11 | 28 | ENST00000003084.6:c.2002C>T | ENSP00000003084.6:p.Arg668Cys |
| chr7:117188797:A:G | rs201434579 | ***CFTR*** | 10 | 24 | ENST00000003084.6:c.1312A>G | ENSP00000003084.6:p.Thr438Ala |
| chr7:117230454:G:C | rs1800098 | ***CFTR*** | 10 | 24 | ENST00000003084.6:c.1727G>C | ENSP00000003084.6:p.Gly576Ala |
| chr7:117188750:C:T | rs201880593 | ***CFTR*** | 10 | 23 | ENST00000003084.6:c.1265C>T | ENSP00000003084.6:p.Ser422Phe |
| chr7:117250575:G:C | rs1800111 | ***CFTR*** | 3 | 6 | ENST00000003084.6:c.2991G>C | ENSP00000003084.6:p.Leu997Phe |
| chr7:117267812:T:G | rs34911792 | ***CFTR*** | 5 | 5 | ENST00000003084.6:c.3705T>G | ENSP00000003084.6:p.Ser1235Arg |
| chr7:117243828:T:C | rs1800110 | ***CFTR*** | 2 | 5 | ENST00000003084.6:c.2900T>C | ENSP00000003084.6:p.Leu967Ser |
| chr7:117171122:T:C | rs35516286 | ***CFTR*** | 4 | 4 | ENST00000003084.6:c.443T>C | ENSP00000003084.6:p.Ile148Thr |
| chr7:117174349:G:A | rs1800079 | ***CFTR*** | 2 | 4 | ENST00000003084.6:c.509G>A | ENSP00000003084.6:p.Arg170His |
| chr7:117188812:G:T | rs147422190 | ***CFTR*** | 0 | 4 | ENST00000003084.6:c.1327G>T | ENSP00000003084.6:p.Asp443Tyr |
| chr7:117199648:T:G | rs74571530 | ***CFTR*** | 4 | 3 | ENST00000003084.6:c.1523T>G | ENSP00000003084.6:p.Phe508Cys |
| chr7:117230411:G:A | rs1800097 | ***CFTR*** | 1 | 3 | ENST00000003084.6:c.1684G>A | ENSP00000003084.6:p.Val562Ile |
| chr7:117180336:C:G | rs1800086 | ***CFTR*** | 3 | 2 | ENST00000003084.6:c.1052C>G | ENSP00000003084.6:p.Thr351Ser |
| chr7:117234999:G:T | rs201386642 | ***CFTR*** | 3 | 2 | ENST00000003084.6:c.2506G>T | ENSP00000003084.6:p.Asp836Tyr |
| chr7:117180242:T:G | rs144476686 | ***CFTR*** | 0 | 2 | ENST00000003084.6:c.958T>G | ENSP00000003084.6:p.Leu320Val |
| chr7:117250625:A:G | rs149279509 | ***CFTR*** | 0 | 2 | ENST00000003084.6:c.3041A>G | ENSP00000003084.6:p.Tyr1014Cys |
| chr7:117292931:C:G | rs80034486 | ***CFTR*** | 0 | 2 | ENST00000003084.6:c.3909C>G | ENSP00000003084.6:p.Asn1303Lys |
| chr7:117149143:C:T | rs115545701 | ***CFTR*** | 1 | 1 | ENST00000003084.6:c.220C>T | ENSP00000003084.6:p.Arg74Trp |
| chr7:117199641:A:G | rs1800091 | ***CFTR*** | 1 | 1 | ENST00000003084.6:c.1516A>G | ENSP00000003084.6:p.Ile506Val |
| chr7:117227809:C:A | rs387906368 | ***CFTR*** | 1 | 1 | ENST00000003084.6:c.1601C>A | ENSP00000003084.6:p.Ala534Glu |
| chr7:117267592:G:T | rs1800120 | ***CFTR*** | 1 | 1 | ENST00000003084.6:c.3485G>T | ENSP00000003084.6:p.Arg1162Leu |
| chr7:117282582:G:A | rs11971167 | ***CFTR*** | 1 | 1 | ENST00000003084.6:c.3808G>A | ENSP00000003084.6:p.Asp1270Asn |
| chr7:117120162:C:T | rs193922501 | ***CFTR*** | 0 | 1 | ENST00000003084.6:c.14C>T | ENSP00000003084.6:p.Pro5Leu |
| chr7:117144344:C:T | rs1800073 | ***CFTR*** | 0 | 1 | ENST00000003084.6:c.91C>T | ENSP00000003084.6:p.Arg31Cys |
| chr7:117144378:C:T | rs143456784 | ***CFTR*** | 0 | 1 | ENST00000003084.6:c.125C>T | ENSP00000003084.6:p.Ser42Phe |
| chr7:117174348:C:T | rs578029902 | ***CFTR*** | 0 | 1 | ENST00000003084.6:c.508C>T | ENSP00000003084.6:p.Arg170Cys |
| chr7:117175339:T:G | rs121908752 | ***CFTR*** | 0 | 1 | ENST00000003084.6:c.617T>G | ENSP00000003084.6:p.Leu206Trp |
| chr7:117180284:C:T | rs121909011 | ***CFTR*** | 0 | 1 | ENST00000003084.6:c.1000C>T | ENSP00000003084.6:p.Arg334Trp |
| chr7:117199575:C:T | rs397508210 | ***CFTR*** | 0 | 1 | ENST00000003084.6:c.1450C>T | ENSP00000003084.6:p.His484Tyr |
| chr7:117227874:A:G | rs75789129 | ***CFTR*** | 0 | 1 | ENST00000003084.6:c.1666A>G | ENSP00000003084.6:p.Ile556Val |
| chr7:117230411:G:C | rs1800097 | ***CFTR*** | 0 | 1 | ENST00000003084.6:c.1684G>C | ENSP00000003084.6:p.Val562Leu |
| chr7:117232394:G:A | rs199791061 | ***CFTR*** | 0 | 1 | ENST00000003084.6:c.2173G>A | ENSP00000003084.6:p.Glu725Lys |
| chr7:117243741:T:G | rs193922511 | ***CFTR*** | 0 | 1 | ENST00000003084.6:c.2813T>G | ENSP00000003084.6:p.Val938Gly |
| chr7:117251771:C:A | rs121908761 | ***CFTR*** | 0 | 1 | ENST00000003084.6:c.3276C>A | ENSP00000003084.6:p.Tyr1092Ter |
| chr7:117251797:T:A | rs36210737 | ***CFTR*** | 0 | 1 | ENST00000003084.6:c.3302T>A | ENSP00000003084.6:p.Met1101Lys |
| chr7:117251857:C:A | rs1266565149 | ***CFTR*** | 0 | 1 | ENST00000003084.6:c.3362C>A | ENSP00000003084.6:p.Thr1121Lys |
| chr7:117254708:A:G | rs397508553 | ***CFTR*** | 0 | 1 | ENST00000003084.6:c.3409A>G | ENSP00000003084.6:p.Met1137Val |
| chr7:117254753:G:C | rs75541969 | ***CFTR*** | 0 | 1 | ENST00000003084.6:c.3454G>C | ENSP00000003084.6:p.Asp1152His |
| chr7:117171029:G:A | rs78655421 | ***CFTR*** | 1 | 0 | ENST00000003084.6:c.350G>A | ENSP00000003084.6:p.Arg117His |
| chr7:117175323:G:A | rs138338446 | ***CFTR*** | 1 | 0 | ENST00000003084.6:c.601G>A | ENSP00000003084.6:p.Val201Met |
| chr7:117176630:A:G | rs191456345 | ***CFTR*** | 1 | 0 | ENST00000003084.6:c.772A>G | ENSP00000003084.6:p.Arg258Gly |
| chr7:117180327:T:A | rs142920240 | ***CFTR*** | 1 | 0 | ENST00000003084.6:c.1043T>A | ENSP00000003084.6:p.Met348Lys |
| chr7:117227824:T:C | rs144745159 | ***CFTR*** | 1 | 0 | ENST00000003084.6:c.1616T>C | ENSP00000003084.6:p.Ile539Thr |
| chr7:117227832:G:T | rs113993959 | ***CFTR*** | 1 | 0 | ENST00000003084.6:c.1624G>T | ENSP00000003084.6:p.Gly542Ter |
| chr7:117232266:CA:C | rs121908746 | ***CFTR*** | 1 | 0 | ENST00000003084.6:c.2052del | ENSP00000003084.6:p.Lys684AsnfsTer38 |
| chr7:117243615:C:T | rs752617117 | ***CFTR*** | 1 | 0 | ENST00000003084.6:c.2687C>T | ENSP00000003084.6:p.Thr896Ile |
| chr7:117243663:C:T | rs121909034 | ***CFTR*** | 1 | 0 | ENST00000003084.6:c.2735C>T | ENSP00000003084.6:p.Ser912Leu |
| chr7:117250664:T:C | rs1800112 | ***CFTR*** | 1 | 0 | ENST00000003084.6:c.3080T>C | ENSP00000003084.6:p.Ile1027Thr |
| chr9:13188803:C:T | rs192227753 | ***MPDZ*** | 6 | 19 | ENST00000319217.7:c.2344G>A | ENSP00000320006.7:p.Gly782Arg |
| chr9:13219603:C:G | rs34911705 | ***MPDZ*** | 9 | 18 | ENST00000319217.7:c.1041G>C | ENSP00000320006.7:p.Leu347Phe |
| chr9:13190162:T:A | rs4740548 | ***MPDZ*** | 10 | 15 | ENST00000319217.7:c.2105A>T | ENSP00000320006.7:p.Glu702Val |
| chr9:13190163:C:T | rs4741289 | ***MPDZ*** | 10 | 15 | ENST00000319217.7:c.2104G>A | ENSP00000320006.7:p.Glu702Lys |
| chr9:13188953:A:T | rs200475640 | ***MPDZ*** | 4 | 5 | ENST00000319217.7:c.2194T>A | ENSP00000320006.7:p.Ser732Thr |
| chr9:13224441:C:A | rs61753782 | ***MPDZ*** | 4 | 4 | ENST00000319217.7:c.325G>T | ENSP00000320006.7:p.Gly109Cys |
| chr9:13136783:T:C | rs200891478 | ***MPDZ*** | 1 | 3 | ENST00000319217.7:c.4220A>G | ENSP00000320006.7:p.Tyr1407Cys |
| chr9:13158062:C:T | rs41265286 | ***MPDZ*** | 1 | 3 | ENST00000319217.7:c.3407G>A | ENSP00000320006.7:p.Ser1136Asn |
| chr9:13223592:G:C | rs181479224 | ***MPDZ*** | 1 | 3 | ENST00000319217.7:c.511C>G | ENSP00000320006.7:p.Gln171Glu |
| chr9:13150558:A:C | rs188840960 | ***MPDZ*** | 0 | 3 | ENST00000319217.7:c.3582T>G | ENSP00000320006.7:p.Ser1194Arg |
| chr9:13123295:T:C | rs16930134 | ***MPDZ*** | 2 | 2 | ENST00000319217.7:c.4810A>G | ENSP00000320006.7:p.Thr1604Ala |
| chr9:13186355:T:C | rs150038177 | ***MPDZ*** | 0 | 2 | ENST00000319217.7:c.2395A>G | ENSP00000320006.7:p.Lys799Glu |
| chr9:13192165:T:C | rs1220556933 | ***MPDZ*** | 0 | 2 | ENST00000319217.7:c.1933A>G | ENSP00000320006.7:p.Ser645Gly |
| chr9:13192285:T:C | rs200101388 | ***MPDZ*** | 0 | 2 | ENST00000319217.7:c.1813A>G | ENSP00000320006.7:p.Ile605Val |
| chr9:13206004:C:T | rs1250100532 | ***MPDZ*** | 0 | 2 | ENST00000319217.7:c.1385G>A | ENSP00000320006.7:p.Arg462Lys |
| chr9:13222321:T:G | rs61753787 | ***MPDZ*** | 0 | 2 | ENST00000319217.7:c.658A>C | ENSP00000320006.7:p.Ile220Leu |
| chr9:13107092:C:T | rs150393677 | ***MPDZ*** | 2 | 1 | ENST00000319217.7:c.6085G>A | ENSP00000320006.7:p.Gly2029Arg |
| chr9:13190292:C:T | rs77838108 | ***MPDZ*** | 2 | 1 | ENST00000319217.7:c.1975G>A | ENSP00000320006.7:p.Val659Ile |
| chr9:13107001:C:T | rs193280665 | ***MPDZ*** | 1 | 1 | ENST00000319217.7:c.6176G>A | ENSP00000320006.7:p.Arg2059Gln |
| chr9:13140070:C:T | rs199509495 | ***MPDZ*** | 1 | 1 | ENST00000319217.7:c.3919G>A | ENSP00000320006.7:p.Glu1307Lys |
| chr9:13110645:A:G | rs545160337 | ***MPDZ*** | 0 | 1 | ENST00000319217.7:c.5819T>C | ENSP00000320006.7:p.Ile1940Thr |
| chr9:13119512:C:T | rs757171783 | ***MPDZ*** | 0 | 1 | ENST00000319217.7:c.5368G>A | ENSP00000320006.7:p.Ala1790Thr |
| chr9:13121870:G:A | rs767824457 | ***MPDZ*** | 0 | 1 | ENST00000319217.7:c.5099C>T | ENSP00000320006.7:p.Thr1700Met |
| chr9:13122130:C:T | rs779435226 | ***MPDZ*** | 0 | 1 | ENST00000319217.7:c.4993G>A | ENSP00000320006.7:p.Ala1665Thr |
| chr9:13126747:T:C | rs749945198 | ***MPDZ*** | 0 | 1 | ENST00000319217.7:c.4489A>G | ENSP00000320006.7:p.Ile1497Val |
| chr9:13136111:C:G | rs778981657 | ***MPDZ*** | 0 | 1 | ENST00000319217.7:c.4363G>C | ENSP00000320006.7:p.Glu1455Gln |
| chr9:13140096:G:A | rs76115127 | ***MPDZ*** | 0 | 1 | ENST00000319217.7:c.3893C>T | ENSP00000320006.7:p.Pro1298Leu |
| chr9:13158039:T:C | rs201227200 | ***MPDZ*** | 0 | 1 | ENST00000319217.7:c.3430A>G | ENSP00000320006.7:p.Ser1144Gly |
| chr9:13183541:T:G | rs1378395732 | ***MPDZ*** | 0 | 1 | ENST00000319217.7:c.2525A>C | ENSP00000320006.7:p.Glu842Ala |
| chr9:13188937:C:T | rs1398326293 | ***MPDZ*** | 0 | 1 | ENST00000319217.7:c.2210G>A | ENSP00000320006.7:p.Gly737Asp |
| chr9:13192278:A:G | rs200777934 | ***MPDZ*** | 0 | 1 | ENST00000319217.7:c.1820T>C | ENSP00000320006.7:p.Leu607Ser |
| chr9:13206038:T:C | rs201448499 | ***MPDZ*** | 0 | 1 | ENST00000319217.7:c.1351A>G | ENSP00000320006.7:p.Thr451Ala |
| chr9:13222276:G:A | rs887434053 | ***MPDZ*** | 0 | 1 | ENST00000319217.7:c.703C>T | ENSP00000320006.7:p.Arg235Cys |
| chr9:13223709:C:T | rs201101621 | ***MPDZ*** | 0 | 1 | ENST00000319217.7:c.394G>A | ENSP00000320006.7:p.Gly132Ser |
| chr9:13224399:C:T | rs367848962 | ***MPDZ*** | 0 | 1 | ENST00000319217.7:c.367G>A | ENSP00000320006.7:p.Asp123Asn |
| chr9:13224410:C:G | rs201953011 | ***MPDZ*** | 0 | 1 | ENST00000319217.7:c.356G>C | ENSP00000320006.7:p.Cys119Ser |
| chr9:13247738:C:T | rs1160966629 | ***MPDZ*** | 0 | 1 | ENST00000319217.7:c.79G>A | ENSP00000320006.7:p.Val27Ile |
| chr9:13250302:T:C | rs200455569 | ***MPDZ*** | 0 | 1 | ENST00000319217.7:c.13A>G | ENSP00000320006.7:p.Ile5Val |
| chr9:13176360:T:A | rs759673011 | ***MPDZ*** | 3 | 0 | ENST00000319217.7:c.2706A>T | ENSP00000320006.7:p.Glu902Asp |
| chr9:13162735:G:C | rs143298483 | ***MPDZ*** | 2 | 0 | ENST00000319217.7:c.3314C>G | ENSP00000320006.7:p.Ser1105Cys |
| chr9:13186292:G:C | rs73647114 | ***MPDZ*** | 2 | 0 | ENST00000319217.7:c.2458C>G | ENSP00000320006.7:p.Leu820Val |
| chr9:13123241:T:C | rs747548775 | ***MPDZ*** | 1 | 0 | ENST00000319217.7:c.4864A>G | ENSP00000320006.7:p.Ile1622Val |
| chr9:13140027:G:A | rs752383203 | ***MPDZ*** | 1 | 0 | ENST00000319217.7:c.3962C>T | ENSP00000320006.7:p.Ser1321Leu |
| chr9:13168470:A:G | rs147599150 | ***MPDZ*** | 1 | 0 | ENST00000319217.7:c.3149T>C | ENSP00000320006.7:p.Ile1050Thr |
| chr9:13168518:C:T | rs376187489 | ***MPDZ*** | 1 | 0 | ENST00000319217.7:c.3101G>A | ENSP00000320006.7:p.Arg1034Gln |
| chr9:13176374:T:C | rs201585415 | ***MPDZ*** | 1 | 0 | ENST00000319217.7:c.2692A>G | ENSP00000320006.7:p.Met898Val |
| chr9:13188785:G:T | rs111794040 | ***MPDZ*** | 1 | 0 | ENST00000319217.7:c.2362C>A | ENSP00000320006.7:p.Pro788Thr |
| chr9:13193228:C:A | rs199726781 | ***MPDZ*** | 1 | 0 | ENST00000319217.7:c.1741G>T | ENSP00000320006.7:p.Val581Phe |
| chr9:13193230:G:C | rs149265684 | ***MPDZ*** | 1 | 0 | ENST00000319217.7:c.1739C>G | ENSP00000320006.7:p.Ser580Cys |
| chr9:13216807:T:A | rs765733279 | ***MPDZ*** | 1 | 0 | ENST00000319217.7:c.1256A>T | ENSP00000320006.7:p.Asp419Val |
| chr9:13217248:C:T | rs199812658 | ***MPDZ*** | 1 | 0 | ENST00000319217.7:c.1132G>A | ENSP00000320006.7:p.Val378Ile |
| chr16:2816519:G:A | rs138447860 | ***SRRM2*** | 6 | 8 | ENST00000301740.8:c.5990G>A | ENSP00000301740.8:p.Arg1997His |
| chr16:2818066:A:G | rs144257955 | ***SRRM2*** | 1 | 7 | ENST00000301740.8:c.7537A>G | ENSP00000301740.8:p.Thr2513Ala |
| chr16:2814950:C:T | rs140421601 | ***SRRM2*** | 3 | 6 | ENST00000301740.8:c.4421C>T | ENSP00000301740.8:p.Ser1474Phe |
| chr16:2817788:C:T | rs150813518 | ***SRRM2*** | 2 | 6 | ENST00000301740.8:c.7259C>T | ENSP00000301740.8:p.Thr2420Ile |
| chr16:2814911:G:T | rs149555495 | ***SRRM2*** | 2 | 5 | ENST00000301740.8:c.4382G>T | ENSP00000301740.8:p.Gly1461Val |
| chr16:2816663:G:A | rs201531430 | ***SRRM2*** | 1 | 5 | ENST00000301740.8:c.6134G>A | ENSP00000301740.8:p.Arg2045His |
| chr16:2813636:G:C | rs72768765 | ***SRRM2*** | 4 | 3 | ENST00000301740.8:c.3107G>C | ENSP00000301740.8:p.Cys1036Ser |
| chr16:2813952:C:G | rs72768766 | ***SRRM2*** | 3 | 3 | ENST00000301740.8:c.3423C>G | ENSP00000301740.8:p.Asp1141Glu |
| chr16:2820436:C:T | rs202157859 | ***SRRM2*** | 1 | 3 | ENST00000301740.8:c.8105C>T | ENSP00000301740.8:p.Ser2702Leu |
| chr16:2817451:G:T | rs145694005 | ***SRRM2*** | 0 | 3 | ENST00000301740.8:c.6922G>T | ENSP00000301740.8:p.Ala2308Ser |
| chr16:2810454:A:G | rs144062317 | ***SRRM2*** | 1 | 2 | ENST00000301740.8:c.986A>G | ENSP00000301740.8:p.Lys329Arg |
| chr16:2820460:A:G | rs149708987 | ***SRRM2*** | 1 | 2 | ENST00000301740.8:c.8129A>G | ENSP00000301740.8:p.Gln2710Arg |
| chr16:2812949:A:C | rs147312180 | ***SRRM2*** | 0 | 2 | ENST00000301740.8:c.2420A>C | ENSP00000301740.8:p.Lys807Thr |
| chr16:2814056:C:G | rs147634161 | ***SRRM2*** | 0 | 2 | ENST00000301740.8:c.3527C>G | ENSP00000301740.8:p.Ala1176Gly |
| chr16:2820684:C:T | rs138495768 | ***SRRM2*** | 2 | 1 | ENST00000301740.8:c.8215C>T | ENSP00000301740.8:p.Pro2739Ser |
| chr16:2810469:C:T | rs150235008 | ***SRRM2*** | 1 | 1 | ENST00000301740.8:c.1001C>T | ENSP00000301740.8:p.Pro334Leu |
| chr16:2811736:C:T | rs778457933 | ***SRRM2*** | 1 | 1 | ENST00000301740.8:c.1207C>T | ENSP00000301740.8:p.Arg403Cys |
| chr16:2812424:G:A | rs766602295 | ***SRRM2*** | 1 | 1 | ENST00000301740.8:c.1895G>A | ENSP00000301740.8:p.Arg632Gln |
| chr16:2819051:C:T | rs111694508 | ***SRRM2*** | 1 | 1 | ENST00000301740.8:c.7787C>T | ENSP00000301740.8:p.Pro2596Leu |
| chr16:2807885:G:A | rs202150099 | ***SRRM2*** | 0 | 1 | ENST00000301740.8:c.454G>A | ENSP00000301740.8:p.Asp152Asn |
| chr16:2807922:C:T | rs141420369 | ***SRRM2*** | 0 | 1 | ENST00000301740.8:c.491C>T | ENSP00000301740.8:p.Ala164Val |
| chr16:2808478:C:T | rs199835386 | ***SRRM2*** | 0 | 1 | ENST00000301740.8:c.523C>T | ENSP00000301740.8:p.Arg175Trp |
| chr16:2809646:G:A | rs200535115 | ***SRRM2*** | 0 | 1 | ENST00000301740.8:c.817G>A | ENSP00000301740.8:p.Asp273Asn |
| chr16:2811712:T:C | rs140983799 | ***SRRM2*** | 0 | 1 | ENST00000301740.8:c.1183T>C | ENSP00000301740.8:p.Ser395Pro |
| chr16:2812567:C:T | rs777371075 | ***SRRM2*** | 0 | 1 | ENST00000301740.8:c.2038C>T | ENSP00000301740.8:p.Arg680Cys |
| chr16:2812843:C:T | rs142703288 | ***SRRM2*** | 0 | 1 | ENST00000301740.8:c.2314C>T | ENSP00000301740.8:p.Arg772Cys |
| chr16:2812972:A:G | rs149861293 | ***SRRM2*** | 0 | 1 | ENST00000301740.8:c.2443A>G | ENSP00000301740.8:p.Ser815Gly |
| chr16:2813177:C:G | rs17136053 | ***SRRM2*** | 0 | 1 | ENST00000301740.8:c.2648C>G | ENSP00000301740.8:p.Ser883Cys |
| chr16:2813392:C:T | rs1354278384 | ***SRRM2*** | 0 | 1 | ENST00000301740.8:c.2863C>T | ENSP00000301740.8:p.Pro955Ser |
| chr16:2813431:A:G | rs749102950 | ***SRRM2*** | 0 | 1 | ENST00000301740.8:c.2902A>G | ENSP00000301740.8:p.Ser968Gly |
| chr16:2813480:C:T | rs371454353 | ***SRRM2*** | 0 | 1 | ENST00000301740.8:c.2951C>T | ENSP00000301740.8:p.Pro984Leu |
| chr16:2813821:C:T | rs773735921 | ***SRRM2*** | 0 | 1 | ENST00000301740.8:c.3292C>T | ENSP00000301740.8:p.Arg1098Trp |
| chr16:2814724:A:T | rs540198317 | ***SRRM2*** | 0 | 1 | ENST00000301740.8:c.4195A>T | ENSP00000301740.8:p.Asn1399Tyr |
| chr16:2815142:G:A | rs140105305 | ***SRRM2*** | 0 | 1 | ENST00000301740.8:c.4613G>A | ENSP00000301740.8:p.Arg1538His |
| chr16:2815784:G:C | rs114848780 | ***SRRM2*** | 0 | 1 | ENST00000301740.8:c.5255G>C | ENSP00000301740.8:p.Arg1752Pro |
| chr16:2816056:C:T | rs549736257 | ***SRRM2*** | 0 | 1 | ENST00000301740.8:c.5527C>T | ENSP00000301740.8:p.Arg1843Trp |
| chr16:2816548:C:T | rs555127784 | ***SRRM2*** | 0 | 1 | ENST00000301740.8:c.6019C>T | ENSP00000301740.8:p.Arg2007Cys |
| chr16:2816627:G:A | rs117133016 | ***SRRM2*** | 0 | 1 | ENST00000301740.8:c.6098G>A | ENSP00000301740.8:p.Arg2033Gln |
| chr16:2816657:G:A | rs139028253 | ***SRRM2*** | 0 | 1 | ENST00000301740.8:c.6128G>A | ENSP00000301740.8:p.Arg2043Gln |
| chr16:2816905:C:T | rs201534509 | ***SRRM2*** | 0 | 1 | ENST00000301740.8:c.6376C>T | ENSP00000301740.8:p.Pro2126Ser |
| chr16:2817356:C:T | rs779240145 | ***SRRM2*** | 0 | 1 | ENST00000301740.8:c.6827C>T | ENSP00000301740.8:p.Ala2276Val |
| chr16:2817667:C:G | rs149458303 | ***SRRM2*** | 0 | 1 | ENST00000301740.8:c.7138C>G | ENSP00000301740.8:p.Leu2380Val |
| chr16:2817833:C:T | rs912779566 | ***SRRM2*** | 0 | 1 | ENST00000301740.8:c.7304C>T | ENSP00000301740.8:p.Pro2435Leu |
| chr16:2817850:C:T | rs61747730 | ***SRRM2*** | 0 | 1 | ENST00000301740.8:c.7321C>T | ENSP00000301740.8:p.Pro2441Ser |
| chr16:2817953:T:C | rs763775653 | ***SRRM2*** | 0 | 1 | ENST00000301740.8:c.7424T>C | ENSP00000301740.8:p.Leu2475Pro |
| chr16:2818060:G:A | rs367790179 | ***SRRM2*** | 0 | 1 | ENST00000301740.8:c.7531G>A | ENSP00000301740.8:p.Ala2511Thr |
| chr16:2818133:C:T | rs772258149 | ***SRRM2*** | 0 | 1 | ENST00000301740.8:c.7604C>T | ENSP00000301740.8:p.Ser2535Leu |
| chr16:2818160:C:T | rs199625043 | ***SRRM2*** | 0 | 1 | ENST00000301740.8:c.7631C>T | ENSP00000301740.8:p.Ser2544Phe |
| chr16:2820709:G:A | rs1205496525 | ***SRRM2*** | 0 | 1 | ENST00000301740.8:c.8240G>A | ENSP00000301740.8:p.Arg2747His |
| chr16:2812573:C:T | rs201743839 | ***SRRM2*** | 1 | 0 | ENST00000301740.8:c.2044C>T | ENSP00000301740.8:p.Arg682Cys |
| chr16:2812786:C:G | rs186552728 | ***SRRM2*** | 1 | 0 | ENST00000301740.8:c.2257C>G | ENSP00000301740.8:p.Arg753Gly |
| chr16:2812946:C:G | rs756755730 | ***SRRM2*** | 1 | 0 | ENST00000301740.8:c.2417C>G | ENSP00000301740.8:p.Ala806Gly |
| chr16:2812975:C:T | rs369213768 | ***SRRM2*** | 1 | 0 | ENST00000301740.8:c.2446C>T | ENSP00000301740.8:p.Arg816Cys |
| chr16:2813779:C:T | rs142025685 | ***SRRM2*** | 1 | 0 | ENST00000301740.8:c.3250C>T | ENSP00000301740.8:p.Pro1084Ser |
| chr16:2814572:C:T | rs549292154 | ***SRRM2*** | 1 | 0 | ENST00000301740.8:c.4043C>T | ENSP00000301740.8:p.Ser1348Phe |
| chr16:2816327:G:A | rs377178914 | ***SRRM2*** | 1 | 0 | ENST00000301740.8:c.5798G>A | ENSP00000301740.8:p.Arg1933His |
| chr16:2816789:G:A | rs200846532 | ***SRRM2*** | 1 | 0 | ENST00000301740.8:c.6260G>A | ENSP00000301740.8:p.Arg2087His |
| chr16:2816815:A:G | rs1338785684 | ***SRRM2*** | 1 | 0 | ENST00000301740.8:c.6286A>G | ENSP00000301740.8:p.Thr2096Ala |
| chr16:2817115:C:T | rs781619820 | ***SRRM2*** | 1 | 0 | ENST00000301740.8:c.6586C>T | ENSP00000301740.8:p.Pro2196Ser |
| chr16:2817974:C:T | rs567024718 | ***SRRM2*** | 1 | 0 | ENST00000301740.8:c.7445C>T | ENSP00000301740.8:p.Thr2482Met |
| chr13:32914592:C:T | rs1799954 | ***BRCA2*** | 11 | 14 | ENST00000380152.3:c.6100C>T | ENSP00000369497.3:p.Arg2034Cys |
| chr13:32972626:A:T | rs11571833 | ***BRCA2*** | 6 | 7 | ENST00000380152.3:c.9976A>T | ENSP00000369497.3:p.Lys3326Ter |
| chr13:32953550:G:A | rs11571769 | ***BRCA2*** | 0 | 6 | ENST00000380152.3:c.8851G>A | ENSP00000369497.3:p.Ala2951Thr |
| chr13:32906766:C:T | rs41293475 | ***BRCA2*** | 0 | 4 | ENST00000380152.3:c.1151C>T | ENSP00000369497.3:p.Ser384Phe |
| chr13:32945108:T:C | rs11571746 | ***BRCA2*** | 1 | 3 | ENST00000380152.3:c.8503T>C | ENSP00000369497.3:p.Ser2835Pro |
| chr13:32893369:G:C | rs28897701 | ***BRCA2*** | 0 | 3 | ENST00000380152.3:c.223G>C | ENSP00000369497.3:p.Ala75Pro |
| chr13:32972695:A:G | rs80358387 | ***BRCA2*** | 0 | 3 | ENST00000380152.3:c.10045A>G | ENSP00000369497.3:p.Thr3349Ala |
| chr13:32914815:G:A | rs35029074 | ***BRCA2*** | 2 | 2 | ENST00000380152.3:c.6323G>A | ENSP00000369497.3:p.Arg2108His |
| chr13:32914839:A:G | rs55953736 | ***BRCA2*** | 0 | 2 | ENST00000380152.3:c.6347A>G | ENSP00000369497.3:p.His2116Arg |
| chr13:32937333:A:G | rs28897745 | ***BRCA2*** | 0 | 2 | ENST00000380152.3:c.7994A>G | ENSP00000369497.3:p.Asp2665Gly |
| chr13:32911295:G:A | rs28897716 | ***BRCA2*** | 1 | 1 | ENST00000380152.3:c.2803G>A | ENSP00000369497.3:p.Asp935Asn |
| chr13:32911754:C:T | rs80358572 | ***BRCA2*** | 1 | 1 | ENST00000380152.3:c.3262C>T | ENSP00000369497.3:p.Pro1088Ser |
| chr13:32912750:G:T | rs28897727 | ***BRCA2*** | 1 | 1 | ENST00000380152.3:c.4258G>T | ENSP00000369497.3:p.Asp1420Tyr |
| chr13:32905128:G:A | rs549269828 | ***BRCA2*** | 0 | 1 | ENST00000380152.3:c.754G>A | ENSP00000369497.3:p.Asp252Asn |
| chr13:32906655:A:G | rs55800493 | ***BRCA2*** | 0 | 1 | ENST00000380152.3:c.1040A>G | ENSP00000369497.3:p.Gln347Arg |
| chr13:32906738:C:T | rs80358408 | ***BRCA2*** | 0 | 1 | ENST00000380152.3:c.1123C>T | ENSP00000369497.3:p.Pro375Ser |
| chr13:32910875:T:G | rs878853563 | ***BRCA2*** | 0 | 1 | ENST00000380152.3:c.2383T>G | ENSP00000369497.3:p.Ser795Ala |
| chr13:32911278:T:C | rs2227943 | ***BRCA2*** | 0 | 1 | ENST00000380152.3:c.2786T>C | ENSP00000369497.3:p.Leu929Ser |
| chr13:32911418:T:A | rs144862123 | ***BRCA2*** | 0 | 1 | ENST00000380152.3:c.2926T>A | ENSP00000369497.3:p.Ser976Thr |
| chr13:32911419:C:T | rs11571656 | ***BRCA2*** | 0 | 1 | ENST00000380152.3:c.2927C>T | ENSP00000369497.3:p.Ser976Phe |
| chr13:32911452:A:T | rs2227944 | ***BRCA2*** | 0 | 1 | ENST00000380152.3:c.2960A>T | ENSP00000369497.3:p.Asn987Ile |
| chr13:32913420:T:C | rs28897731 | ***BRCA2*** | 0 | 1 | ENST00000380152.3:c.4928T>C | ENSP00000369497.3:p.Val1643Ala |
| chr13:32914196:G:A | rs4987048 | ***BRCA2*** | 0 | 1 | ENST00000380152.3:c.5704G>A | ENSP00000369497.3:p.Asp1902Asn |
| chr13:32914515:A:AG | rs80359554 | ***BRCA2*** | 0 | 1 | ENST00000380152.3:c.6024dup | ENSP00000369497.3:p.Gln2009AlafsTer9 |
| chr13:32914849:C:G | rs1064794110 | ***BRCA2*** | 0 | 1 | ENST00000380152.3:c.6357C>G | ENSP00000369497.3:p.Asn2119Lys |
| chr13:32930673:C:T | rs28897744 | ***BRCA2*** | 0 | 1 | ENST00000380152.3:c.7544C>T | ENSP00000369497.3:p.Thr2515Ile |
| chr13:32932020:C:T | rs56335340 | ***BRCA2*** | 0 | 1 | ENST00000380152.3:c.7759C>T | ENSP00000369497.3:p.Leu2587Phe |
| chr13:32937375:A:G | rs80359041 | ***BRCA2*** | 0 | 1 | ENST00000380152.3:c.8036A>G | ENSP00000369497.3:p.Asp2679Gly |
| chr13:32937488:G:T | rs28897747 | ***BRCA2*** | 0 | 1 | ENST00000380152.3:c.8149G>T | ENSP00000369497.3:p.Ala2717Ser |
| chr13:32945123:A:G | rs80359103 | ***BRCA2*** | 0 | 1 | ENST00000380152.3:c.8518A>G | ENSP00000369497.3:p.Ile2840Val |
| chr13:32953529:A:T | rs4987047 | ***BRCA2*** | 0 | 1 | ENST00000380152.3:c.8830A>T | ENSP00000369497.3:p.Ile2944Phe |
| chr13:32971119:A:G | rs80359228 | ***BRCA2*** | 0 | 1 | ENST00000380152.3:c.9586A>G | ENSP00000369497.3:p.Lys3196Glu |
| chr13:32971146:G:C | rs528504546 | ***BRCA2*** | 0 | 1 | ENST00000380152.3:c.9613G>C | ENSP00000369497.3:p.Ala3205Pro |
| chr13:32971147:C:T | rs546682485 | ***BRCA2*** | 0 | 1 | ENST00000380152.3:c.9614C>T | ENSP00000369497.3:p.Ala3205Val |
| chr13:32953549:G:T | rs28897754 | ***BRCA2*** | 2 | 0 | ENST00000380152.3:c.8850G>T | ENSP00000369497.3:p.Lys2950Asn |
| chr13:32906892:A:C | rs1421854019 | ***BRCA2*** | 1 | 0 | ENST00000380152.3:c.1277A>C | ENSP00000369497.3:p.Lys426Thr |
| chr13:32911737:A:G | rs80358569 | ***BRCA2*** | 1 | 0 | ENST00000380152.3:c.3245A>G | ENSP00000369497.3:p.Lys1082Arg |
| chr13:32912007:C:T | rs80358600 | ***BRCA2*** | 1 | 0 | ENST00000380152.3:c.3515C>T | ENSP00000369497.3:p.Ser1172Leu |
| chr13:32914132:T:G | rs11571657 | ***BRCA2*** | 1 | 0 | ENST00000380152.3:c.5640T>G | ENSP00000369497.3:p.Asn1880Lys |
| chr13:32945130:G:T | rs80359105 | ***BRCA2*** | 1 | 0 | ENST00000380152.3:c.8525G>T | ENSP00000369497.3:p.Arg2842Leu |
| chr13:32953617:G:A | rs80359143 | ***BRCA2*** | 1 | 0 | ENST00000380152.3:c.8918G>A | ENSP00000369497.3:p.Arg2973His |
| chr13:32953971:C:T | rs28897755 | ***BRCA2*** | 1 | 0 | ENST00000380152.3:c.9038C>T | ENSP00000369497.3:p.Thr3013Ile |
| chr13:32954037:A:C | rs80359165 | ***BRCA2*** | 1 | 0 | ENST00000380152.3:c.9104A>C | ENSP00000369497.3:p.Tyr3035Ser |
| chr1:151502589:G:A | rs41272459 | ***CGN*** | 13 | 19 | ENST00000271636.7:c.2311G>A | ENSP00000271636.7:p.Glu771Lys |
| chr1:151505035:G:A | rs115631509 | ***CGN*** | 3 | 5 | ENST00000271636.7:c.2729G>A | ENSP00000271636.7:p.Arg910Gln |
| chr1:151496718:C:T | rs16833336 | ***CGN*** | 4 | 3 | ENST00000271636.7:c.1285C>T | ENSP00000271636.7:p.Arg429Cys |
| chr1:151491026:C:T | rs144766457 | ***CGN*** | 1 | 3 | ENST00000271636.7:c.31C>T | ENSP00000271636.7:p.Arg11Trp |
| chr1:151491836:C:T | rs140720174 | ***CGN*** | 1 | 3 | ENST00000271636.7:c.841C>T | ENSP00000271636.7:p.Arg281Trp |
| chr1:151501912:G:C | rs199971698 | ***CGN*** | 0 | 3 | ENST00000271636.7:c.1983G>C | ENSP00000271636.7:p.Glu661Asp |
| chr1:151504956:C:T | rs201154454 | ***CGN*** | 0 | 2 | ENST00000271636.7:c.2650C>T | ENSP00000271636.7:p.Arg884Trp |
| chr1:151508133:T:A | rs766782113 | ***CGN*** | 0 | 2 | ENST00000271636.7:c.3052T>A | ENSP00000271636.7:p.Cys1018Ser |
| chr1:151502487:C:T | rs140407178 | ***CGN*** | 1 | 1 | ENST00000271636.7:c.2209C>T | ENSP00000271636.7:p.Arg737Trp |
| chr1:151503185:G:A | rs559028450 | ***CGN*** | 1 | 1 | ENST00000271636.7:c.2534G>A | ENSP00000271636.7:p.Arg845His |
| chr1:151491591:G:A | rs375611149 | ***CGN*** | 0 | 1 | ENST00000271636.7:c.596G>A | ENSP00000271636.7:p.Arg199His |
| chr1:151492646:A:G | rs200681495 | ***CGN*** | 0 | 1 | ENST00000271636.7:c.878A>G | ENSP00000271636.7:p.Lys293Arg |
| chr1:151496719:G:A | rs761721175 | ***CGN*** | 0 | 1 | ENST00000271636.7:c.1286G>A | ENSP00000271636.7:p.Arg429His |
| chr1:151501842:A:C | rs761802775 | ***CGN*** | 0 | 1 | ENST00000271636.7:c.1913A>C | ENSP00000271636.7:p.Gln638Pro |
| chr1:151501908:G:A | rs116788396 | ***CGN*** | 0 | 1 | ENST00000271636.7:c.1979G>A | ENSP00000271636.7:p.Arg660Gln |
| chr1:151501929:C:T | rs567100227 | ***CGN*** | 0 | 1 | ENST00000271636.7:c.2000C>T | ENSP00000271636.7:p.Ala667Val |
| chr1:151502527:G:C | rs144180892 | ***CGN*** | 0 | 1 | ENST00000271636.7:c.2249G>C | ENSP00000271636.7:p.Arg750Pro |
| chr1:151502568:C:T | rs148329413 | ***CGN*** | 0 | 1 | ENST00000271636.7:c.2290C>T | ENSP00000271636.7:p.Arg764Trp |
| chr1:151504890:G:A | rs142577828 | ***CGN*** | 0 | 1 | ENST00000271636.7:c.2584G>A | ENSP00000271636.7:p.Gly862Arg |
| chr1:151508308:G:C | rs779972131 | ***CGN*** | 0 | 1 | ENST00000271636.7:c.3131G>C | ENSP00000271636.7:p.Ser1044Thr |
| chr1:151496009:A:C | rs145912252 | ***CGN*** | 7 | 0 | ENST00000271636.7:c.1240A>C | ENSP00000271636.7:p.Lys414Gln |
| chr1:151491500:A:C | rs761066980 | ***CGN*** | 1 | 0 | ENST00000271636.7:c.505A>C | ENSP00000271636.7:p.Thr169Pro |
| chr1:151491620:C:T | rs763549292 | ***CGN*** | 1 | 0 | ENST00000271636.7:c.625C>T | ENSP00000271636.7:p.Arg209Cys |
| chr1:151499464:C:T | rs548839520 | ***CGN*** | 1 | 0 | ENST00000271636.7:c.1777C>T | ENSP00000271636.7:p.Arg593Ter |
| chr1:151499510:A:G | rs16833349 | ***CGN*** | 1 | 0 | ENST00000271636.7:c.1823A>G | ENSP00000271636.7:p.Glu608Gly |
| chr1:151502484:C:T | rs760131604 | ***CGN*** | 1 | 0 | ENST00000271636.7:c.2206C>T | ENSP00000271636.7:p.Arg736Cys |
| chr1:120468201:A:T | rs41313282 | ***NOTCH2*** | 6 | 15 | ENST00000256646.2:c.4238T>A | ENSP00000256646.2:p.Leu1413His |
| chr1:120458122:A:T | rs35586704 | ***NOTCH2*** | 7 | 9 | ENST00000256646.2:c.7223T>A | ENSP00000256646.2:p.Leu2408His |
| chr1:120548095:C:A | rs143195893 | ***NOTCH2*** | 0 | 7 | ENST00000256646.2:c.272G>T | ENSP00000256646.2:p.Arg91Leu |
| chr1:120458270:G:C | rs75831573 | ***NOTCH2*** | 3 | 6 | ENST00000256646.2:c.7075C>G | ENSP00000256646.2:p.Pro2359Ala |
| chr1:120469147:T:C | rs61752484 | ***NOTCH2*** | 6 | 5 | ENST00000256646.2:c.3980A>G | ENSP00000256646.2:p.Asp1327Gly |
| chr1:120459122:C:T | rs150516342 | ***NOTCH2*** | 0 | 4 | ENST00000256646.2:c.6223G>A | ENSP00000256646.2:p.Val2075Met |
| chr1:120496243:G:C | rs782306821 | ***NOTCH2*** | 0 | 3 | ENST00000256646.2:c.2288C>G | ENSP00000256646.2:p.Ser763Trp |
| chr1:120484350:A:C | rs782079844 | ***NOTCH2*** | 0 | 2 | ENST00000256646.2:c.2780T>G | ENSP00000256646.2:p.Met927Arg |
| chr1:120464916:C:T | rs587737953 | ***NOTCH2*** | 3 | 1 | ENST00000256646.2:c.5156G>A | ENSP00000256646.2:p.Arg1719Gln |
| chr1:120478125:A:C | rs147223770 | ***NOTCH2*** | 1 | 1 | ENST00000256646.2:c.3625T>G | ENSP00000256646.2:p.Phe1209Val |
| chr1:120458570:C:T | rs1054703109 | ***NOTCH2*** | 0 | 1 | ENST00000256646.2:c.6775G>A | ENSP00000256646.2:p.Val2259Met |
| chr1:120459251:G:T | rs143236410 | ***NOTCH2*** | 0 | 1 | ENST00000256646.2:c.6094C>A | ENSP00000256646.2:p.His2032Asn |
| chr1:120465339:T:G | rs148354054 | ***NOTCH2*** | 0 | 1 | ENST00000256646.2:c.4922A>C | ENSP00000256646.2:p.Lys1641Thr |
| chr1:120465364:C:T | rs116321057 | ***NOTCH2*** | 0 | 1 | ENST00000256646.2:c.4897G>A | ENSP00000256646.2:p.Val1633Ile |
| chr1:120479928:A:C | - | ***NOTCH2*** | 0 | 1 | ENST00000256646.2:c.3499T>G | ENSP00000256646.2:p.Phe1167Val |
| chr1:120480048:T:G | - | ***NOTCH2*** | 0 | 1 | ENST00000256646.2:c.3379A>C | ENSP00000256646.2:p.Ile1127Leu |
| chr1:120491644:G:A | rs35656321 | ***NOTCH2*** | 0 | 1 | ENST00000256646.2:c.2585C>T | ENSP00000256646.2:p.Ala862Val |
| chr1:120506257:C:T | rs782493871 | ***NOTCH2*** | 0 | 1 | ENST00000256646.2:c.1855G>A | ENSP00000256646.2:p.Asp619Asn |
| chr1:120539937:G:A | rs150730704 | ***NOTCH2*** | 0 | 1 | ENST00000256646.2:c.434C>T | ENSP00000256646.2:p.Thr145Met |
| chr1:120548173:C:T | rs377214276 | ***NOTCH2*** | 0 | 1 | ENST00000256646.2:c.194G>A | ENSP00000256646.2:p.Arg65Gln |
| chr1:120464355:G:A | rs909117249 | ***NOTCH2*** | 1 | 0 | ENST00000256646.2:c.5291C>T | ENSP00000256646.2:p.Pro1764Leu |
| chr1:120471613:C:T | rs201968231 | ***NOTCH2*** | 1 | 0 | ENST00000256646.2:c.3878G>A | ENSP00000256646.2:p.Arg1293His |
| chr1:120471712:C:T | rs75423398 | ***NOTCH2*** | 1 | 0 | ENST00000256646.2:c.3779G>A | ENSP00000256646.2:p.Arg1260His |
| chr1:120479948:T:C | rs142876168 | ***NOTCH2*** | 1 | 0 | ENST00000256646.2:c.3479A>G | ENSP00000256646.2:p.His1160Arg |
| chr1:120548029:C:T | rs140965343 | ***NOTCH2*** | 1 | 0 | ENST00000256646.2:c.338G>A | ENSP00000256646.2:p.Arg113Gln |
| chr1:120548101:G:A | rs138537504 | ***NOTCH2*** | 1 | 0 | ENST00000256646.2:c.266C>T | ENSP00000256646.2:p.Thr89Met |
| chr16:81942175:A:G | rs75472618 | ***PLCG2*** | 18 | 47 | ENST00000359376.3:c.1712A>G | ENSP00000352336.3:p.Asn571Ser |
| chr16:81939089:T:C | rs187956469 | ***PLCG2*** | 4 | 11 | ENST00000359376.3:c.1444T>C | ENSP00000352336.3:p.Tyr482His |
| chr16:81929485:T:C | rs138637229 | ***PLCG2*** | 4 | 9 | ENST00000359376.3:c.1146T>C | ENSP00000352336.3:p.Phe382Phe |
| chr16:81946278:A:G | rs150833842 | ***PLCG2*** | 4 | 9 | ENST00000359376.3:c.2011A>G | ENSP00000352336.3:p.Ile671Val |
| chr16:81942028:C:G | rs72824905 | ***PLCG2*** | 2 | 7 | ENST00000359376.3:c.1565C>G | ENSP00000352336.3:p.Pro522Arg |
| chr16:81971435:G:C | rs114262189 | ***PLCG2*** | 1 | 4 | ENST00000359376.3:c.3125G>C | ENSP00000352336.3:p.Ser1042Thr |
| chr16:81925132:C:T | rs199636472 | ***PLCG2*** | 1 | 3 | ENST00000359376.3:c.923C>T | ENSP00000352336.3:p.Ala308Val |
| chr16:81957106:A:G | rs142825971 | ***PLCG2*** | 0 | 3 | ENST00000359376.3:c.2324A>G | ENSP00000352336.3:p.Lys775Arg |
| chr16:81968131:A:G | rs139462941 | ***PLCG2*** | 1 | 1 | ENST00000359376.3:c.2837A>G | ENSP00000352336.3:p.Asn946Ser |
| chr16:81990459:C:G | rs200800716 | ***PLCG2*** | 1 | 1 | ENST00000359376.3:c.3730C>G | ENSP00000352336.3:p.Leu1244Val |
| chr16:81819704:C:A | rs147349332 | ***PLCG2*** | 0 | 1 | ENST00000359376.3:c.110C>A | ENSP00000352336.3:p.Thr37Asn |
| chr16:81888178:C:T | rs535715020 | ***PLCG2*** | 0 | 1 | ENST00000359376.3:c.323C>T | ENSP00000352336.3:p.Thr108Met |
| chr16:81925134:G:A | rs768664050 | ***PLCG2*** | 0 | 1 | ENST00000359376.3:c.925G>A | ENSP00000352336.3:p.Val309Met |
| chr16:81934281:G:A | rs201490178 | ***PLCG2*** | 0 | 1 | ENST00000359376.3:c.1258G>A | ENSP00000352336.3:p.Ala420Thr |
| chr16:81939094:G:A | rs201746780 | ***PLCG2*** | 0 | 1 | ENST00000359376.3:c.1449G>A | ENSP00000352336.3:p.Met483Ile |
| chr16:81953195:G:A | rs187454354 | ***PLCG2*** | 0 | 1 | ENST00000359376.3:c.2161G>A | ENSP00000352336.3:p.Glu721Lys |
| chr16:81957094:A:G | rs201803492 | ***PLCG2*** | 0 | 1 | ENST00000359376.3:c.2312A>G | ENSP00000352336.3:p.Gln771Arg |
| chr16:81957175:A:G | rs117077093 | ***PLCG2*** | 0 | 1 | ENST00000359376.3:c.2393A>G | ENSP00000352336.3:p.Asn798Ser |
| chr16:81819671:C:T | rs189301790 | ***PLCG2*** | 1 | 0 | ENST00000359376.3:c.77C>T | ENSP00000352336.3:p.Thr26Met |
| chr7:131815315:G:A | rs150228337 | ***PLXNA4*** | 8 | 13 | ENST00000321063.4:c.5608C>T | ENSP00000323194.4:p.His1870Tyr |
| chr7:132193371:G:A | rs113830939 | ***PLXNA4*** | 4 | 4 | ENST00000321063.4:c.82C>T | ENSP00000323194.4:p.Arg28Trp |
| chr7:131925916:C:T | rs112682233 | ***PLXNA4*** | 2 | 3 | ENST00000321063.4:c.1513G>A | ENSP00000323194.4:p.Val505Ile |
| chr7:131982880:G:T | rs200301458 | ***PLXNA4*** | 2 | 3 | ENST00000321063.4:c.1473C>A | ENSP00000323194.4:p.His491Gln |
| chr7:132192810:C:T | rs142904267 | ***PLXNA4*** | 1 | 3 | ENST00000321063.4:c.643G>A | ENSP00000323194.4:p.Ala215Thr |
| chr7:132193334:A:G | rs141077562 | ***PLXNA4*** | 0 | 3 | ENST00000321063.4:c.119T>C | ENSP00000323194.4:p.Phe40Ser |
| chr7:132174152:C:T | rs142997259 | ***PLXNA4*** | 2 | 2 | ENST00000321063.4:c.1270G>A | ENSP00000323194.4:p.Val424Ile |
| chr7:131870089:C:T | rs117458710 | ***PLXNA4*** | 1 | 2 | ENST00000321063.4:c.3127G>A | ENSP00000323194.4:p.Val1043Met |
| chr7:131895706:G:A | rs201526825 | ***PLXNA4*** | 1 | 2 | ENST00000321063.4:c.2294C>T | ENSP00000323194.4:p.Thr765Ile |
| chr7:131832686:C:T | rs201134213 | ***PLXNA4*** | 1 | 1 | ENST00000321063.4:c.4837G>A | ENSP00000323194.4:p.Val1613Ile |
| chr7:131853172:C:T | rs757554246 | ***PLXNA4*** | 0 | 1 | ENST00000321063.4:c.4177G>A | ENSP00000323194.4:p.Val1393Met |
| chr7:131853327:G:A | rs374466534 | ***PLXNA4*** | 0 | 1 | ENST00000321063.4:c.4022C>T | ENSP00000323194.4:p.Pro1341Leu |
| chr7:131864567:A:C | rs1182362410 | ***PLXNA4*** | 0 | 1 | ENST00000321063.4:c.3753T>G | ENSP00000323194.4:p.Ile1251Met |
| chr7:131865404:C:T | rs770408803 | ***PLXNA4*** | 0 | 1 | ENST00000321063.4:c.3580G>A | ENSP00000323194.4:p.Val1194Met |
| chr7:131865470:C:T | rs749537432 | ***PLXNA4*** | 0 | 1 | ENST00000321063.4:c.3514G>A | ENSP00000323194.4:p.Val1172Met |
| chr7:131866259:C:T | rs544769667 | ***PLXNA4*** | 0 | 1 | ENST00000321063.4:c.3373G>A | ENSP00000323194.4:p.Val1125Ile |
| chr7:131870086:G:A | rs773666064 | ***PLXNA4*** | 0 | 1 | ENST00000321063.4:c.3130C>T | ENSP00000323194.4:p.Arg1044Trp |
| chr7:131870104:C:G | rs200939541 | ***PLXNA4*** | 0 | 1 | ENST00000321063.4:c.3112G>C | ENSP00000323194.4:p.Glu1038Gln |
| chr7:131883374:G:A | rs772814018 | ***PLXNA4*** | 0 | 1 | ENST00000321063.4:c.2608C>T | ENSP00000323194.4:p.Arg870Trp |
| chr7:131888127:C:T | rs374819408 | ***PLXNA4*** | 0 | 1 | ENST00000321063.4:c.2350G>A | ENSP00000323194.4:p.Val784Met |
| chr7:131895860:C:T | rs1308095027 | ***PLXNA4*** | 0 | 1 | ENST00000321063.4:c.2140G>A | ENSP00000323194.4:p.Val714Met |
| chr7:131908372:G:A | rs199651527 | ***PLXNA4*** | 0 | 1 | ENST00000321063.4:c.2011C>T | ENSP00000323194.4:p.Arg671Cys |
| chr7:131908378:G:A | rs757302168 | ***PLXNA4*** | 0 | 1 | ENST00000321063.4:c.2005C>T | ENSP00000323194.4:p.Pro669Ser |
| chr7:131910930:C:T | rs771852886 | ***PLXNA4*** | 0 | 1 | ENST00000321063.4:c.1972G>A | ENSP00000323194.4:p.Val658Ile |
| chr7:132174125:T:C | rs139356539 | ***PLXNA4*** | 0 | 1 | ENST00000321063.4:c.1297A>G | ENSP00000323194.4:p.Thr433Ala |
| chr7:132192824:G:A | rs754366028 | ***PLXNA4*** | 0 | 1 | ENST00000321063.4:c.629C>T | ENSP00000323194.4:p.Ala210Val |
| chr7:132193185:C:T | rs746205942 | ***PLXNA4*** | 0 | 1 | ENST00000321063.4:c.268G>A | ENSP00000323194.4:p.Glu90Lys |
| chr7:131865473:G:A | rs192855926 | ***PLXNA4*** | 3 | 0 | ENST00000321063.4:c.3511C>T | ENSP00000323194.4:p.Pro1171Ser |
| chr7:131844259:G:A | rs777611144 | ***PLXNA4*** | 1 | 0 | ENST00000321063.4:c.4633C>T | ENSP00000323194.4:p.Arg1545Trp |
| chr7:131848913:G:C | rs905061285 | ***PLXNA4*** | 1 | 0 | ENST00000321063.4:c.4488C>G | ENSP00000323194.4:p.Asp1496Glu |
| chr7:131910920:G:A | rs756961353 | ***PLXNA4*** | 1 | 0 | ENST00000321063.4:c.1982C>T | ENSP00000323194.4:p.Ser661Leu |
| chr7:131912297:A:C | rs759656711 | ***PLXNA4*** | 1 | 0 | ENST00000321063.4:c.1795T>G | ENSP00000323194.4:p.Ser599Ala |
| chr7:131913220:C:T | rs371929100 | ***PLXNA4*** | 1 | 0 | ENST00000321063.4:c.1613G>A | ENSP00000323194.4:p.Arg538Gln |
| chr7:131982882:G:T | rs764488585 | ***PLXNA4*** | 1 | 0 | ENST00000321063.4:c.1471C>A | ENSP00000323194.4:p.His491Asn |
| chr7:132174113:C:T | rs531099216 | ***PLXNA4*** | 1 | 0 | ENST00000321063.4:c.1309G>A | ENSP00000323194.4:p.Ala437Thr |
| chr7:132192639:T:C | rs1344143685 | ***PLXNA4*** | 1 | 0 | ENST00000321063.4:c.814A>G | ENSP00000323194.4:p.Lys272Glu |
| chr2:206641239:TCGCA:T | - | ***NRP2*** | 28 | 45 | ENST00000272849.3:c.2712_2715del | ENSP00000272849.3:p.His905AlafsTer34 |
| chr2:206641245:T:TA | rs200483574 | ***NRP2*** | 28 | 45 | ENST00000272849.3:c.2716_2717insA | ENSP00000272849.3:p.Cys906Ter |
| chr2:206592624:C:T | rs114144673 | ***NRP2*** | 1 | 3 | ENST00000272849.3:c.1000C>T | ENSP00000272849.3:p.Arg334Cys |
| chr2:206659543:G:A | rs201258364 | ***NRP2*** | 0 | 3 | ENST00000357785.5:c.2542G>A | ENSP00000350432.5:p.Asp848Asn |
| chr2:206608085:G:A | rs762753258 | ***NRP2*** | 1 | 2 | ENST00000272849.3:c.1450G>A | ENSP00000272849.3:p.Gly484Ser |
| chr2:206590654:C:T | rs79750907 | ***NRP2*** | 0 | 1 | ENST00000272849.3:c.838C>T | ENSP00000272849.3:p.Pro280Ser |
| chr2:206592643:C:T | rs150064130 | ***NRP2*** | 0 | 1 | ENST00000272849.3:c.1019C>T | ENSP00000272849.3:p.Thr340Met |
| chr2:206614482:C:T | rs1240190640 | ***NRP2*** | 0 | 1 | ENST00000272849.3:c.1820C>T | ENSP00000272849.3:p.Thr607Ile |
| chr2:206614493:G:A | rs114848039 | ***NRP2*** | 0 | 1 | ENST00000272849.3:c.1831G>A | ENSP00000272849.3:p.Glu611Lys |
| chr2:206628646:G:A | rs552306916 | ***NRP2*** | 0 | 1 | ENST00000272849.3:c.2293G>A | ENSP00000272849.3:p.Asp765Asn |
| chr2:206640979:T:G | rs147520159 | ***NRP2*** | 0 | 1 | ENST00000272849.3:c.2450T>G | ENSP00000272849.3:p.Leu817Arg |
| chr2:206588569:C:T | rs138033888 | ***NRP2*** | 1 | 0 | ENST00000272849.3:c.725C>T | ENSP00000272849.3:p.Thr242Met |
| chr2:206590778:A:T | rs151124318 | ***NRP2*** | 1 | 0 | ENST00000272849.3:c.962A>T | ENSP00000272849.3:p.Asn321Ile |
| chr9:71844124:G:A | rs41277901 | ***TJP2*** | 9 | 21 | ENST00000265384.7:c.1478G>A | ENSP00000265384.7:p.Arg493Lys |
| chr9:71831325:C:T | rs138241615 | ***TJP2*** | 4 | 4 | ENST00000265384.7:c.185C>T | ENSP00000265384.7:p.Thr62Met |
| chr9:71851040:C:G | rs149911553 | ***TJP2*** | 0 | 3 | ENST00000265384.7:c.1877C>G | ENSP00000265384.7:p.Thr626Ser |
| chr9:71852004:T:C | rs35797487 | ***TJP2*** | 4 | 2 | ENST00000265384.7:c.2131T>C | ENSP00000265384.7:p.Ser711Pro |
| chr9:71840944:G:C | rs78681604 | ***TJP2*** | 0 | 2 | ENST00000265384.7:c.1063G>C | ENSP00000265384.7:p.Gly355Arg |
| chr9:71840972:C:T | rs77321498 | ***TJP2*** | 0 | 2 | ENST00000265384.7:c.1091C>T | ENSP00000265384.7:p.Thr364Met |
| chr9:71853685:G:A | rs145368713 | ***TJP2*** | 6 | 1 | ENST00000265384.7:c.2335G>A | ENSP00000265384.7:p.Val779Met |
| chr9:71863112:G:A | rs368776552 | ***TJP2*** | 3 | 1 | ENST00000265384.7:c.2852G>A | ENSP00000265384.7:p.Arg951His |
| chr9:71852010:A:G | rs116545275 | ***TJP2*** | 1 | 1 | ENST00000265384.7:c.2137A>G | ENSP00000265384.7:p.Ser713Gly |
| chr9:71866274:T:G | rs752545256 | ***TJP2*** | 1 | 1 | ENST00000377245.4:c.3315T>G | ENSP00000366453.4:p.Asn1105Lys |
| chr9:71820123:A:G | rs151117327 | ***TJP2*** | 0 | 1 | ENST00000535702.1:c.46A>G | ENSP00000442090.1:p.Lys16Glu |
| chr9:71827482:C:G | rs145628692 | ***TJP2*** | 0 | 1 | ENST00000265384.7:c.79C>G | ENSP00000265384.7:p.Leu27Val |
| chr9:71831336:A:G | rs754335612 | ***TJP2*** | 0 | 1 | ENST00000265384.7:c.196A>G | ENSP00000265384.7:p.Ile66Val |
| chr9:71863084:G:A | rs766312893 | ***TJP2*** | 0 | 1 | ENST00000265384.7:c.2824G>A | ENSP00000265384.7:p.Glu942Lys |
| chr9:71866149:G:T | rs199892018 | ***TJP2*** | 0 | 1 | ENST00000377245.4:c.3190G>T | ENSP00000366453.4:p.Val1064Leu |
| chr9:71866186:A:G | rs372741297 | ***TJP2*** | 0 | 1 | ENST00000377245.4:c.3227A>G | ENSP00000366453.4:p.Lys1076Arg |
| chr9:71836347:G:A | rs376434139 | ***TJP2*** | 1 | 0 | ENST00000265384.7:c.887G>A | ENSP00000265384.7:p.Ser296Asn |
| chr9:71840232:G:A | rs559824029 | ***TJP2*** | 1 | 0 | ENST00000265384.7:c.965G>A | ENSP00000265384.7:p.Arg322Gln |
| chr9:71840285:A:G | rs763206171 | ***TJP2*** | 1 | 0 | ENST00000265384.7:c.1018A>G | ENSP00000265384.7:p.Lys340Glu |
| chr9:71864319:G:A | rs150494393 | ***TJP2*** | 1 | 0 | ENST00000377245.4:c.2909G>A | ENSP00000366453.4:p.Arg970Gln |

| **Supplementary Table 6**. Odds ratios (95% Confidence Interval; *p*-value) resulting from sensitivity analysis using logistic regressions of patient-recorded variables for the ARDS risk-associated gene clusters with minimal similarity. Results from models adjusted for sex, age, and APACHE II scores, excluding and including first two principal components, and from models that did not adjust for any covariate are included for reference. | | | | | | | | | | | |  |
| --- | --- | --- | --- | --- | --- | --- | --- | --- | --- | --- | --- | --- |
| **Cluster ID** | **Top enriched pathway** | **Sex + Age + APACHE II** | **Sex + Age + APACHE II + PC1 + PC2** | | **Unadjusted** | **Sex** | | | **Age** | **BMI** | |  |
| 12 | Amino acids regulate mTORC1 | 6.57 (2.75-15.68;  *p*=2.0x10^-5^) | 6.76 (2.66-17.15; *p*=6x10^-5^) | | 6.05 (2.59-14.10; *p*=3.2x10^-5^) | 6.03 (2.58-14.10; *p*=3.3x10^-5^) | | | 6.09 (2.60-14.20; *p*=3.0x10^-5^) | 4.06 (1.55-10.60; *p*=4.27x10^-3^) | |  |
| 170 | Toll-like receptor cascades | 3.72 (2.05-6.71; *p*=1.0x10^-5^) | 3.85 (2.09-7.09; *p*=1x10^-5^) | | 3.95 (2.21-7.05; *p*=3.6x10^-6^) | 4.02 (2.25-7.20; *p*=2.7x10^-6^) | | | 3.93 (2.20-7.04; *p*=3.8x10^-6^) | 5.41 (2.23-13.20; *p*=1.9x10^-4^) | |  |
| 169 | Regulation of white adipocyte differentiation | 3.05 (1.61-5.80; *p*=6.3x10^-4^) | 2.83 (1.52-5.28; *p*=1.02x10^-3^) | | 2.95 (1.60-5.44; *p*=5.0x10^-4^) | 2.99 (1.62-5.51; *p*=4.4x10^-4^) | | | 3.00 (1.63-5.53;  *p*=4.2x10^-4^) | 2.75 (1.25-6.05; *p*=0.012) | |  |
| 186 | Integrin cell surface interactions | 6.10 (2.15-17.29; *p*=6.6x10^-4^) | 4.22 (1.63-10.9; *p*=2.8x10^-3^) | | 5.11 (2.01-13.00; *p*=6.13x10^-4^) | 5.15 (2.02-13.10; *p*=5.8x10^-4^) | | | 5.13 (2.02-13.00;  *p*=5.9x10^-4^) | 6.57 (1.53-28.20; *p*=0.011) | |  |
| 153 | Interferon signaling | 2.61 (1.48-4.61; *p*=9.2x10^-4^) | 2.98 (1.64-5.42; *p*=3.3x10^-4^) | | 2.92 (1.68-5.10; *p*=1.53x10^-4^) | 2.88 (1.65-5.03; *p*=1.9x10^-4^) | | | 2.93 (1.68-5.11; *p*=1.4x10^-4^) | 2.08 (1.03-4.18, *p*=0.041) | |  |
| 97 | DNA repair | 2.78 (1.46-5.29; *p*=1.8x10^-3^) | 2.57 (1.31-5.05; *p*=6.07x10^-3^) | | 3.01 (1.60-5.66; *p*=6.51x10^-4^) | 3.05 (1.62-5.74; *p*=5.7x10^-4^) | | | 3.03 (1.61-5.70; *p*=6.1x10^-4^) | 2.64 (1.13-6.19; *p*=0.025) | |  |
| 19 | Protein-protein interactions at synapses | 3.88 (1.86-8.07; *p*=2.8x10^-4^) | 3.84 (1.78-8.26; *p*=5.7x10^-4^) | | 2.96 (1.53-5.72; *p*=1.2x10^-3^) | 3.14 (1.62-6.08; *p*=7.1x10^-4^) | | | 2.96 (1.53-5.72; *p*=1.2x10^-3^) | 2.76 (1.12-6.82; *p*=0.027) | |  |
| 182 | mTOR signaling | 2.63 (1.30-5.34; *p*=7.2x10^-3^) | 2.17 (1.06-4.43;  *p*=0.032) | | 2.74 (1.37-5.47; *p*=4.4x10^-3^) | 2.70 (1.35-5.40; *p*=5.1x10^-3^) | | | 2.69 (1.34-5.38; *p*=5.2x10^-3^) | 2.18 (0.79-5.95; *p*=0.128) | |  |
| 46 | mRNA splicing | 3.42 (1.65-7.09; *p*=9.2x10^-4^) | 4.77 (2.01-11.36;  *p*=4x10^-4^) | | 3.44 (1.68-7.06; *p*=7.3x10^-4^) | 3.53 (1.72-7.25; *p*=5.8x10^-4^) | | | 3.48 (1.70-7.14; *p*=6.6x10^-4^) | 2.88 (1.16-7.13; *p*=2.24x10^-2^) | |  |
| 181 | Krebs cycle and respiratory electron transport | 3.04 (1.75-5.28; *p*=7.0x10^-5^) | 3.47 (1.92-6.28;  *p*=4x10^-5^) | | 3.13 (1.83-5.37; *p*=3.2x10^-5^) | 3.13 (1.83-5.38; *p*=3.3x10^-5^) | | | 3.14 (1.83-5.38; *p*=3.2x10^-5^) | 2.63 (1.28-5.39; *p*=8.26x10^-3^) | |  |
|  |  |  |  | |  |  | | |  |  | |  |
| **Cluster ID** | **SOFA** | **APACHE II** | | **Days from diagnosis** | **ICU mortality** | | **Source of infection** | | **Pathogen identified** | | **Patients with comorbidities** |  |
| 12 | 6.81 (2.68-17.30;  *p*=5.5x10^-5^) | 6.21 (2.63-14.70; *p*=3.2x10^-5^) | | 6.52 (2.57-16.60;  *p*=8.1x10^-5^) | 5.94 (2.52-14.00;  *p*=4.7x10^-5^) | | 6.91 (2.72-17.50; *p*=4.7x10^-5^) | | 6.02 (2.58-14.10; *p*=3.3x10^-5^) | | 5.75 (2.46-13.50; *p*=5.6x10^-5^) |  |
| 170 | 4.40 (2.28-8.48; *p*=1.0x10^-5^) | 3.63 (2.02-6.52;  *p*=1.6x10^-5^) | | 3.80 (2.02-7.15; *p*=3.3x10^-5^) | 4.06 (2.21-7.45;  *p*=6.2x10^-6^) | | 4.25 (2.25-8.00;  *p*=7.7x10^-6^) | | 3.94 (2.2-7.04;  *p*=3.7x10^-6^) | | 4.02 (2.20-7.34;  *p*=5.7x10^-6^) |  |
| 169 | 2.90 (1.51-5.56; *p*=1.3x10^-3^) | 2.92 (1.55-5.53;  *p*=9.5x10^-4^) | | 3.61 (1.80-7.26; *p*=3.07x10^-4^) | 3.35 (1.76-6.38;  *p*=2.2x10^-4^) | | 3.48 (1.74-6.98; *p*=4.3x10^-4^) | | 2.95 (1.60-5.43;  *p*=5.2x10^-4^) | | 2.82 (1.53-5.21; *p*=9.3x10^-4^) |  |
| 186 | 10.50 (2.49-44.00; *p*=1.3x10^-3^) | 5.89 (2.09-16.60; *p*=7.9x10^-4^) | | 10.50 (2.52-44.10;  *p*=1.2x10^-3^) | 6.67 (2.35-18.90;  *p*=3.6x10^-4^) | | 7.58 (2.31-24.90; *p*=8.4x10^-4^) | | 5.14 (2.02-13.10; *p*=5.8x10^-4^) | | 6.15 (2.19-17.30; *p*=5.6x10^-4^) |  |
| 153 | 2.78 (1.55-4.97; *p*=5.7x10^-4^) | 2.65 (1.51-4.67;  *p*=7.0x10^-4^) | | 3.01 (1.68-5.38; *p*=2.1x10^-4^) | 2.82 (1.60-4.96;  *p*=3.3x10^-4^) | | 2.68 (1.50-4.81; *p*=9.3x10^-4^) | | 2.93 (1.68-5.10;  *p*=1.5x10^-4^) | | 2.84 (1.63-4.97; *p*=2.4x10^-4^) |  |
| 97 | 2.55 (1.31-4.99; *p*=6.1x10^-3^) | 2.72 (1.43-5.14; *p*=2.1x10^-3^) | | 2.97 (1.48-5.95; *p*=2.1x10^-3^) | 3.23 (1.66-6.30;  *p*=5.6x10^-4^) | | 3.14 (1.59-6.18; *p*=9.5x10^-4^) | | 3.00 (1.60-5.65;  *p*=6.5x10^-4^) | | 2.90 (1.53-5.48; *p*=1.0x10^-3^) |  |
| 19 | 2.84 (1.41-5.71; *p*=3.5x10^-3^) | 3.57 (1.73-7.37; *p*=5.6x10^-4^) | | 3.32 (1.47-7.47; *p*=3.8x10^-3^) | 3.01 (1.46-6.21;  *p*=2.8x10^-3^) | | 3.39 (1.57-7.32; *p*=1.9x10^-3^) | | 2.95 (1.52-5.70;  *p*=1.3x10^-3^) | | 3.18 (1.60-6.34; *p*=9.6x10^-4^) |  |
| 182 | 2.61 (1.19-5.69; *p*=0.016) | 2.74 (1.36-5.53; *p*=4.7x10^-3^) | | 2.65 (1.22-5.77; *p*=0.014) | 2.75 (1.36-5.56;  *p*=5x10^-3^) | | | 2.76 (1.31-5.84; *p*=7.8x10^-3^) | 2.79 (1.39-5.60; *p*=3.7x10^-3^) | 2.69 (1.34-5.39;  *p*=5.2x10^-3^) | |  |
| 46 | 3.26 (1.51-7.05; *p*=2.6x10^-3^) | 3.25 (1.58-6.71; *p*=1.4x10^-3^) | | 3.56 (1.58-8.05; *p*=2.2x10^-3^) | 3.21 (1.55-6.64;  *p*=1.6x10^-3^) | | | 3.54 (1.63-7.66; *p*=1.3x10^-3^) | 3.45 (1.68-7.08; *p*=7.2x10^-4^) | 3.36 (1.63-6.92; *p*=9.9x10^-4^) | |  |
| 181 | 3.18 (1.77-5.70; *p*=1.0x10^-4^) | 3.00 (1.73-5.18; *p*=8.4x10^-5^) | | 3.00 (1.68-5.35; *p*=2.1x10^-4^) | 3.14 (1.79-5.49;  *p*=6.2x10^-5^) | | | 2.78 (1.58-4.91; *p*=4.1x10^-4^) | 3.13 (1.82-5.36;  *p*=3.3x10^-5^) | 3.59 (2.04-6.33;  *p*=1x10^-5^) | |  |
|  | | | | | | | | | | | | |

| **Supplementary Table 7.** Results from sensibility analyses of QVs with AF≤ 0.01 and AF ≤ 0.001 in gene clusters with minimal similarity. The models were adjusted for sex, age and APACHE II scores. | | | |
| --- | --- | --- | --- |
|  | **QVs-CADD10 (AF≤0.01)** | **QVs-CADD15 (AF≤0.001)** |  |
| **Top enriched pathway** | **Odds Ratio (95% Confidence Interval); p-value** | |  |
| Amino acids regulate mTORC1 | NAN† | 1.63 (1.13-2.36); *p=*8.39x10^-3^ |  |
| Toll-like receptor cascades | 30.1 (4.1-222); *p*=8.17x10^-4^ | 1.29 (0.92-1.79); *p=*0.128 |  |
| Regulation of white adipocyte differentiation | 25.3 (3.46-185); *p*=1.45x10^-3^ | 1.54 (1.07-2.22); *p=*0.018 |  |
| Integrin cell surface interactions | NAN† | 1.39 (0.95-2.03); *p=*0.083 |  |
| Interferon signaling | 7.1 (2.53-19.90); *p*=1.96x10^-4^ | 1.45 (1.02-2.07); *p=*0.034 |  |
| DNA repair | 12.1 (2.89-50.40); *p*=6.37x10^-4^ | 1.51 (1.06-2.16); *p=*0.022 |  |
| Protein-protein interactions at synapses | 26.2 (3.54-194); *p*=1.38x10^-3^ | 1.27 (0.89-1.80); *p=*0.182 |  |
| mTOR signaling | 11.1 (2.63-46.60); *p*=1.05x10^-3^ | 1.53 (1.04-2.24); *p=*0.029 |  |
| mRNA splicing | 8.66 (2.65-28.30); *p*=3.50x10^-4^ | 0.95 (0.69-1.31); *p=*0.781 |  |
| Krebs cycle and respiratory electron transport | 3.91 (1.89-8.08); *p*=2.35x10^-4^ | 1.59 (1.15-2.21); *p=*4.98x10^-3^ |  |
| CI: confidence interval. ^†^ Models not converging, attributable to disproportionate distribution of carriers and non-carriers and leading to unreliable effect size estimates. | | | |

| **Supplementary Table 8** Results of the null model based on logistic regression analysis of rare (AF≤0.01) synonymous variants in gene clusters with minimal similarity. The models were adjusted for sex, age and APACHE II scores. | | |  |
| --- | --- | --- | --- |
| **Cluster ID** | **Top enriched pathway** | **Odds Ratio (95% Confidence Interval); p-value** | |
| 12 | Amino acids regulate mTORC1 | 1.27 (0.79-2.04); p=0.320 | |
| 170 | Toll-like receptor cascades | 1.56 (0.83-2.95); p=0.170 | |
| 169 | Regulation of white adipocyte differentiation | 1.66 (0.87-3.17); p=0.120 | |
| 186 | Integrin cell surface interactions | 1.03 (0.57-1.85); p=0.920 | |
| 153 | Interferon signaling | 0.94 (0.55-1.60); p=0.810 | |
| 97 | DNA repair | 0.84 (0.48-1.49); p=0.550 | |
| 19 | Protein-protein interactions at synapses | 0.90 (0.61-1.33); p=0.590 | |
| 182 | mTOR signaling | 2.16 (0.71-6.55); p=0.170 | |
| 46 | mRNA splicing | 0.66 (0.435-0.99); p=0.048 | |
| 181 | Krebs cycle and respiratory electron transport | 0.80 (0.55-1.16); p=0.230 | |
|  | | |  |

| Supplementary Figures**Supplementary Fig. 1** Plot of the first two principal components (PCs) of genetic variation of GEN-SEP patients and of data from the 1000 Genomes Project from African (AFR), American (AMR), East Asian (EAS), European (EUR), and South Asian (SAS) populations. GEN-SEP cases and controls are indicated with differentiated symbols. The percentages represent the explained variability of each axis.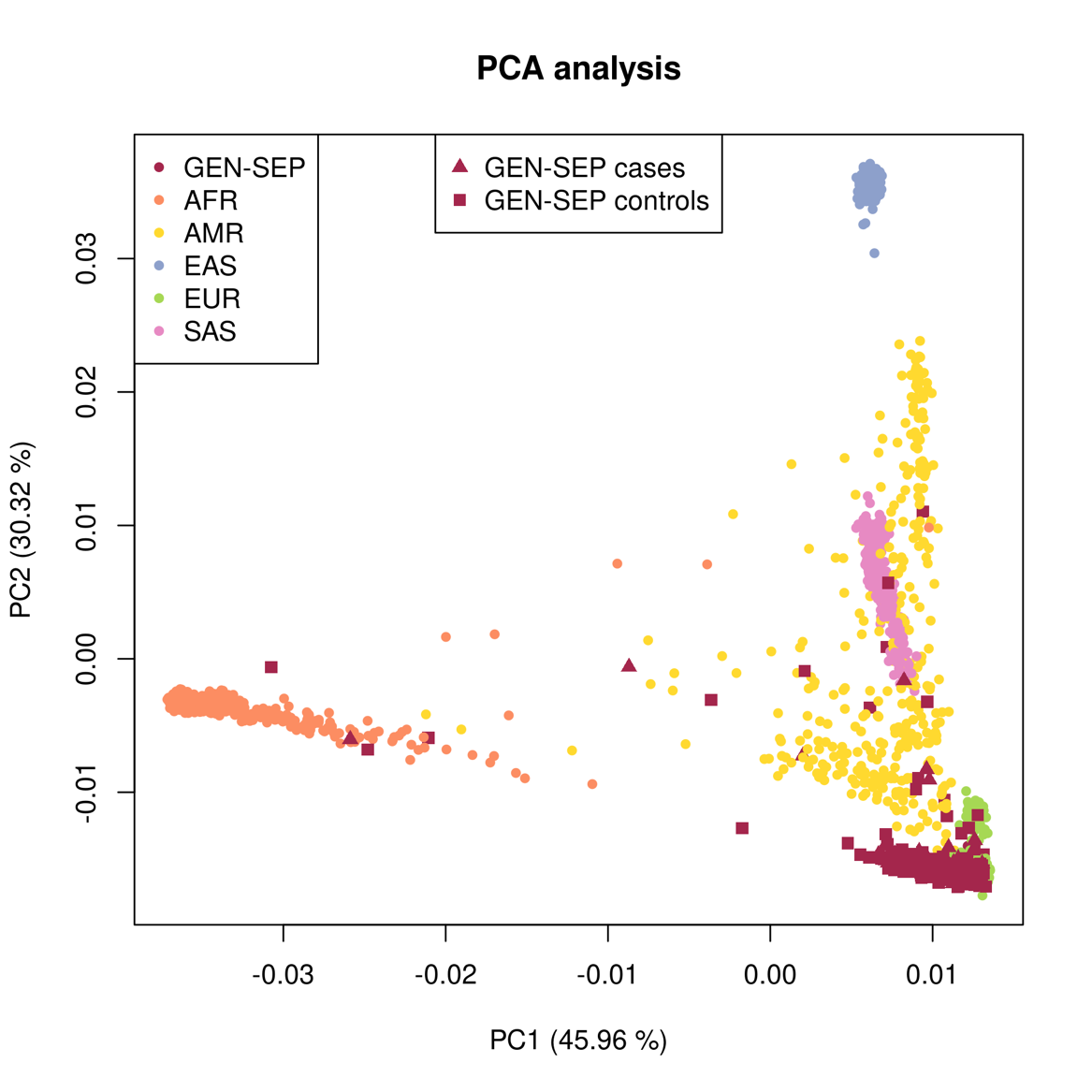 |
| --- |

##

##
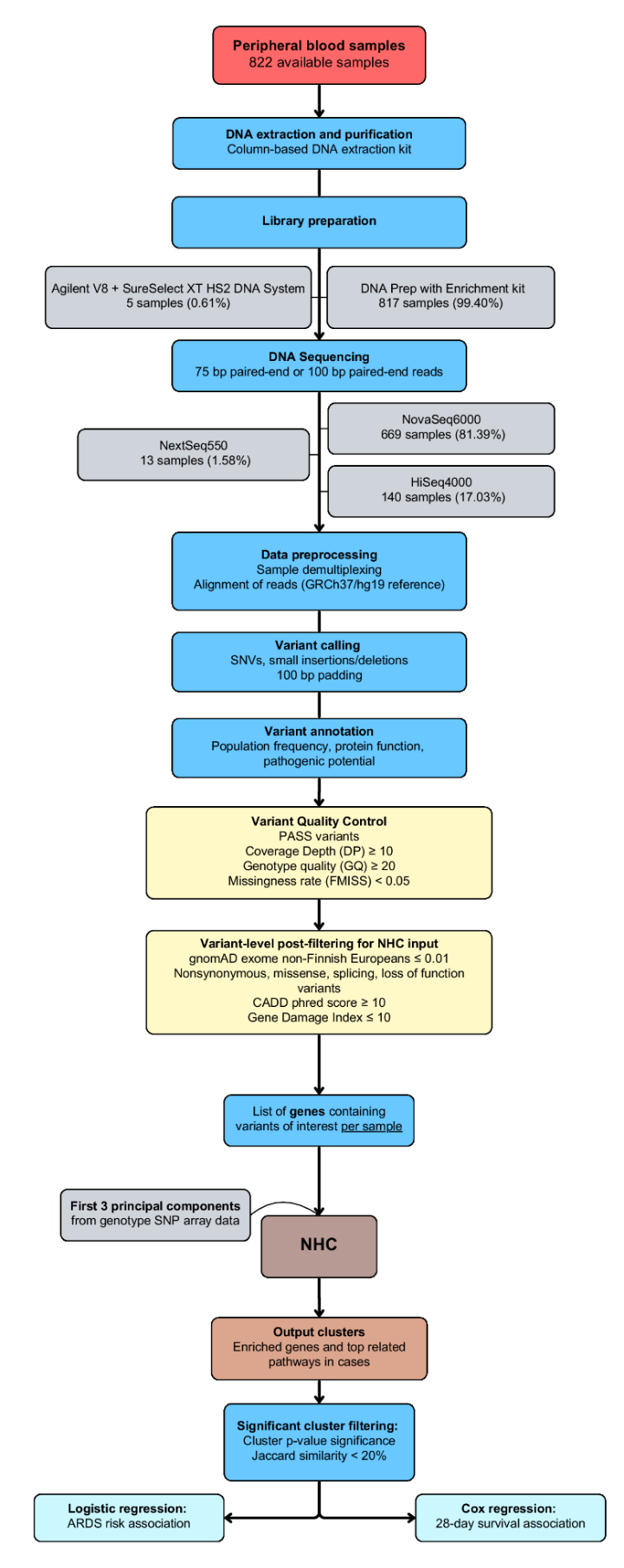
**Supplementary Fig. 2** Schematic overview of the sequencing workflow, filtering for qualifying variant identification, and the network-based heterogeneity clustering (NHC) analysis.

## **Supplementary Fig. 3** Similarity matrix, as the inverse of the Jaccard distance, reflecting the percentage of gene-level overlap between significant gene clusters. Similarity between clusters is expressed in percentage (%) and by the color intensity of each box. Clusters with similarity >20% are highlighted in bold.

**
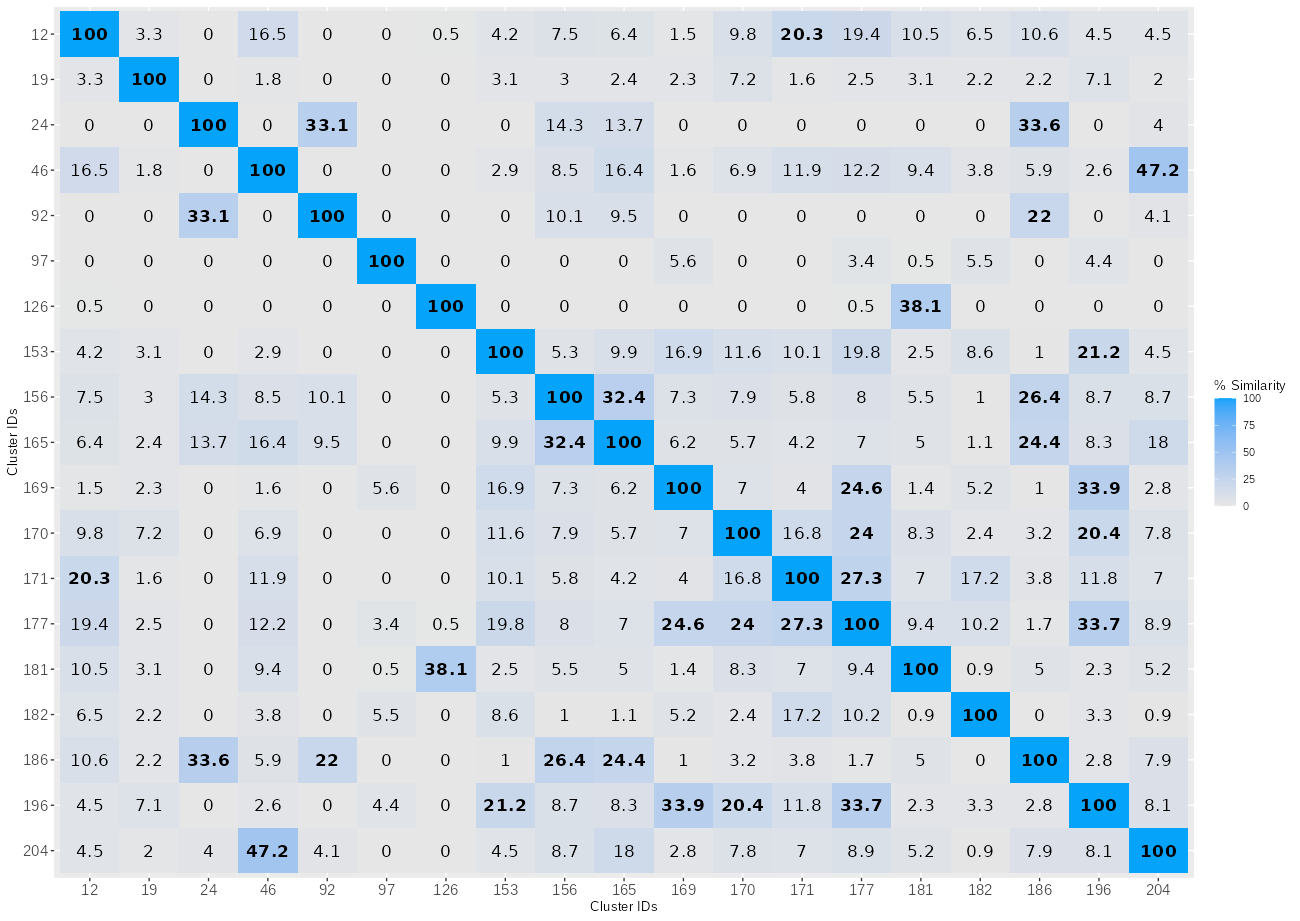
**

##
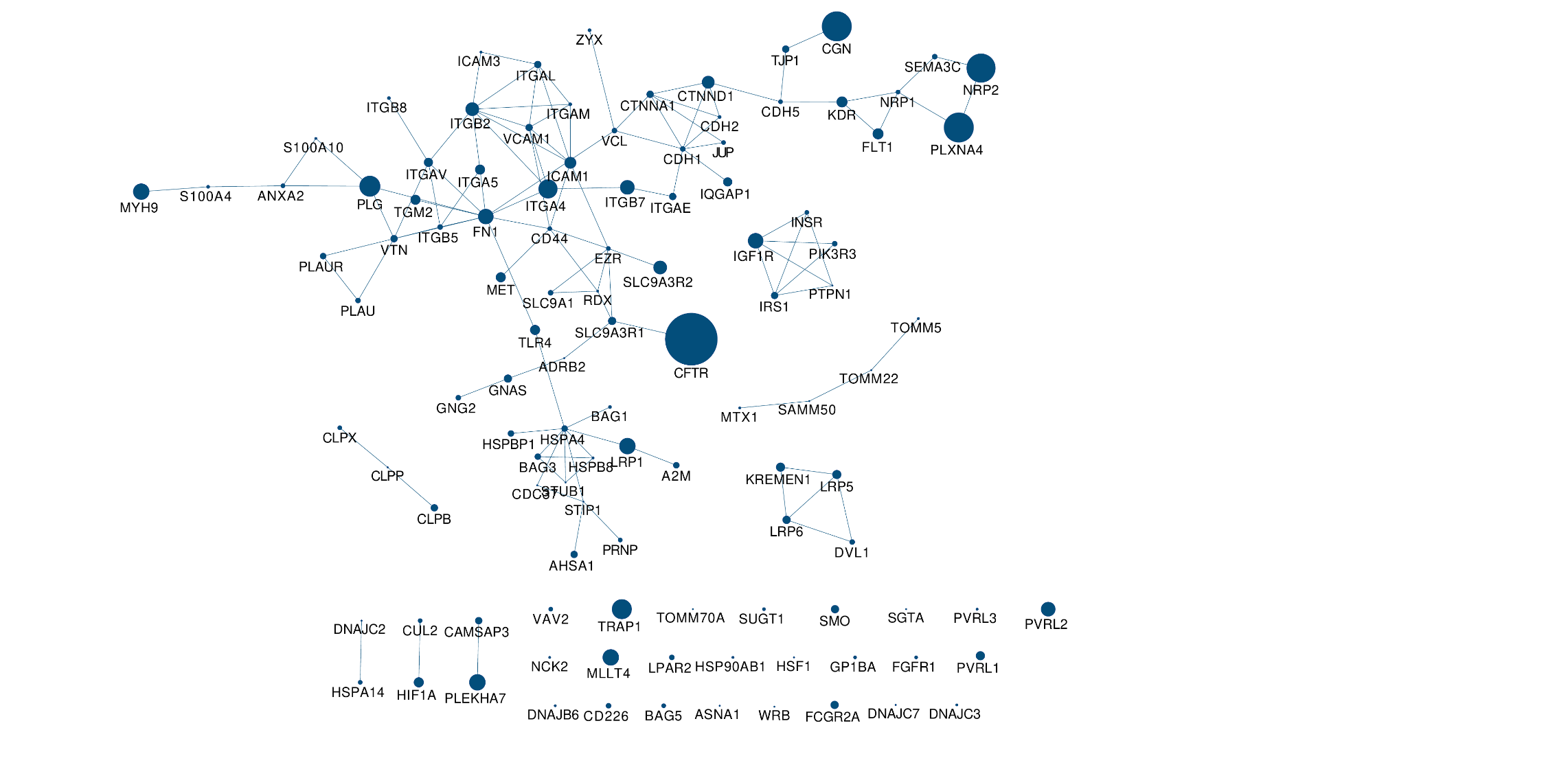
**Supplementary Fig. 4** Network representation of Cluster 186, the most significant among the significant clusters with the *Integrin cell surface interactions* as the top-most significant associated pathway. Each node represents a gene in the cluster and the node size indicates the number of ARDS cases harboring qualifying variants in that gene. The lines connecting the nodes represent robust protein-protein interactions according to NHC edge-weighted background network.

## **Supplementary Fig. 5** Network representation of Cluster 170, the most significant among the significant clusters with the *Toll-like receptor cascades* as the top-most significant associated pathway. Each node represents a gene in the cluster and the node size indicates the number of ARDS patients harboring qualifying variants in that gene. The lines connecting the nodes represent robust protein-protein interactions according to NHC edge-weighted background network.


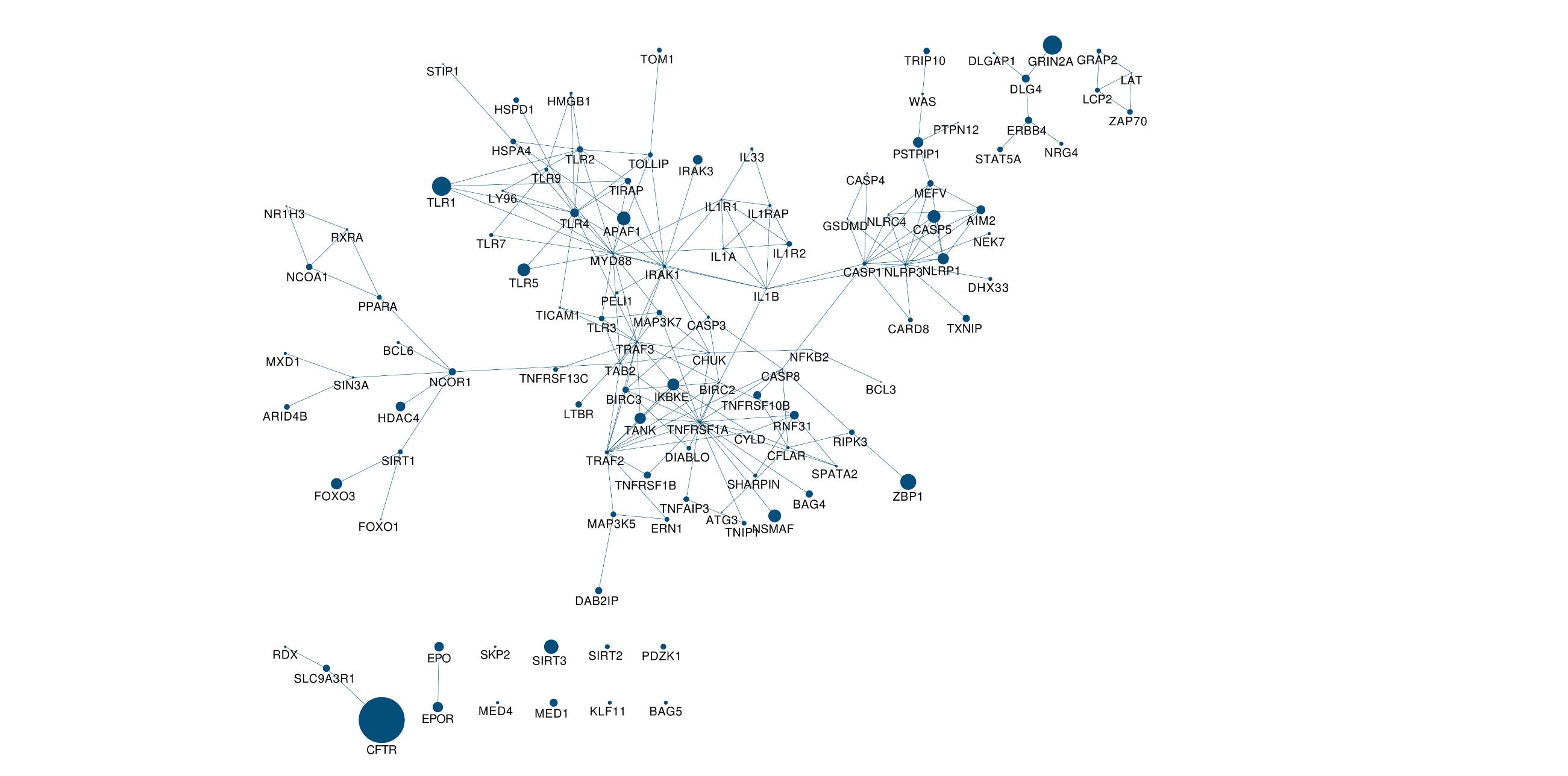


## **
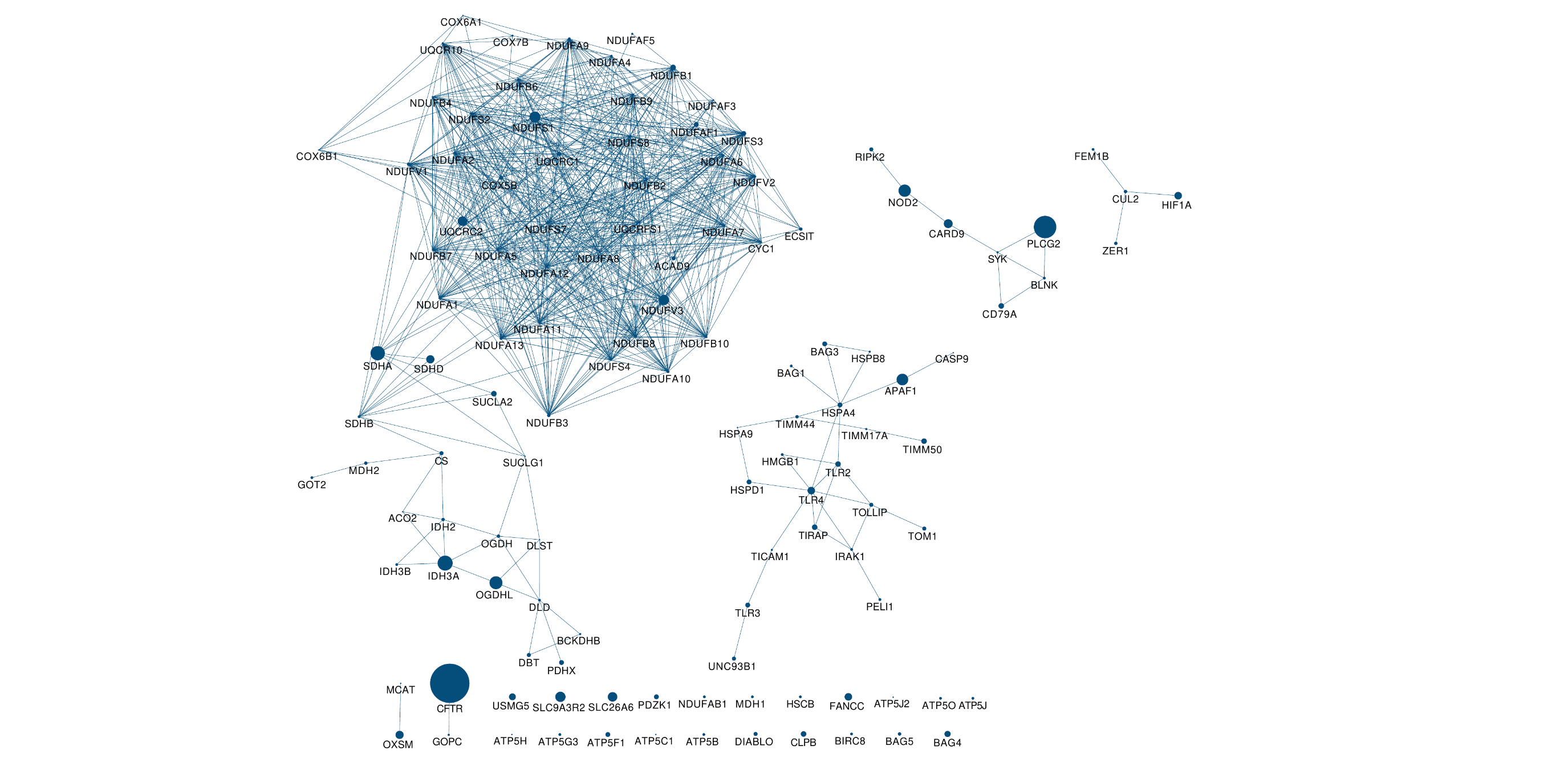
Supplementary Fig. 6** Network representation of Cluster 181, the most significant among the significant clusters with the *Krebs cycle and respiratory electron transport* as the top-most significant associated pathway. Each node represents a gene in the cluster and the node size indicates the number of ARDS patients harboring qualifying variants in that gene. The lines connecting the nodes represent robust protein-protein interactions according to NHC edge-weighted background network.

## **Supplementary Fig. 7** Network representation of Cluster 169, the only significant cluster with *Transcriptional regulation of white adipocyte differentiation* as the top-most significant associated pathway. Each node represents a gene in the cluster and the node size indicates the number of ARDS patients harboring qualifying variants in that gene. The lines connecting the nodes represent robust protein-protein interactions according to NHC edge-weighted background network.


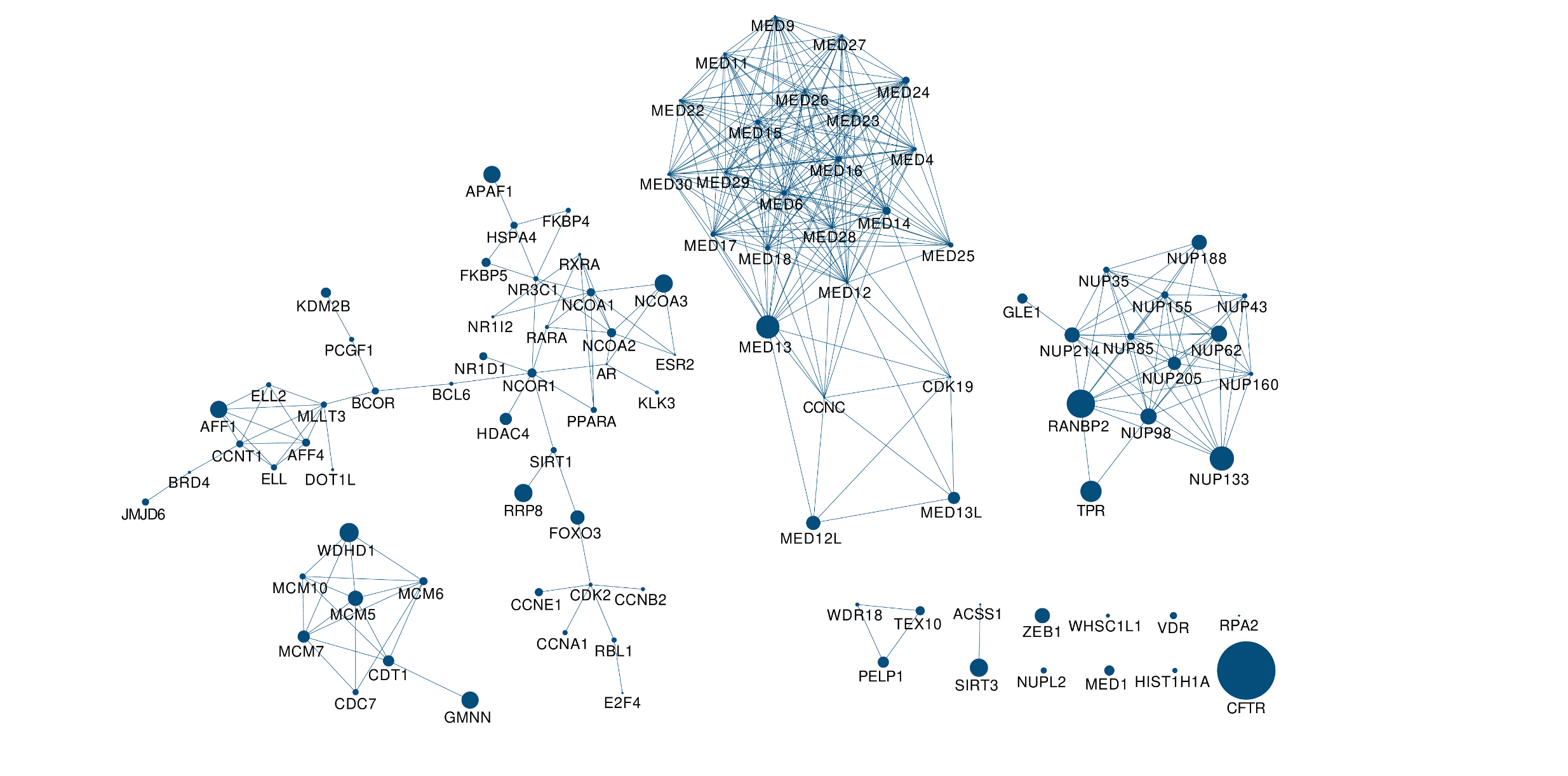


## **Supplementary Fig. 8** Network representation of Cluster 153, the most significant among the significant clusters with the *Interferon signaling* as the top-most significant associated biological pathway. Each node represents a gene in the cluster and the node size indicates the number of ARDS patients harboring qualifying variants in that gene. The lines connecting the nodes represent robust protein-protein interactions according to NHC edge-weighted background network.

**
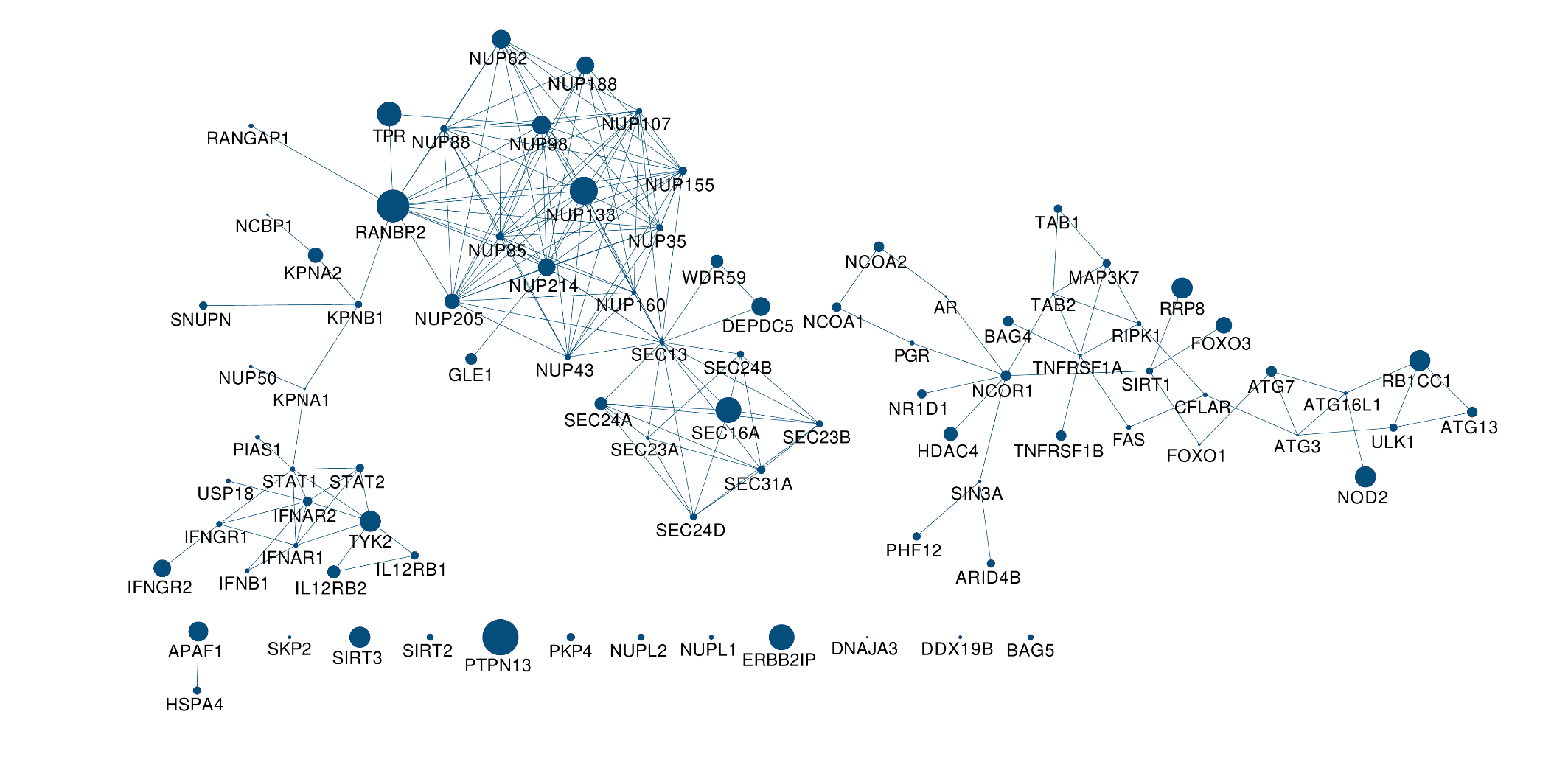
**

## **
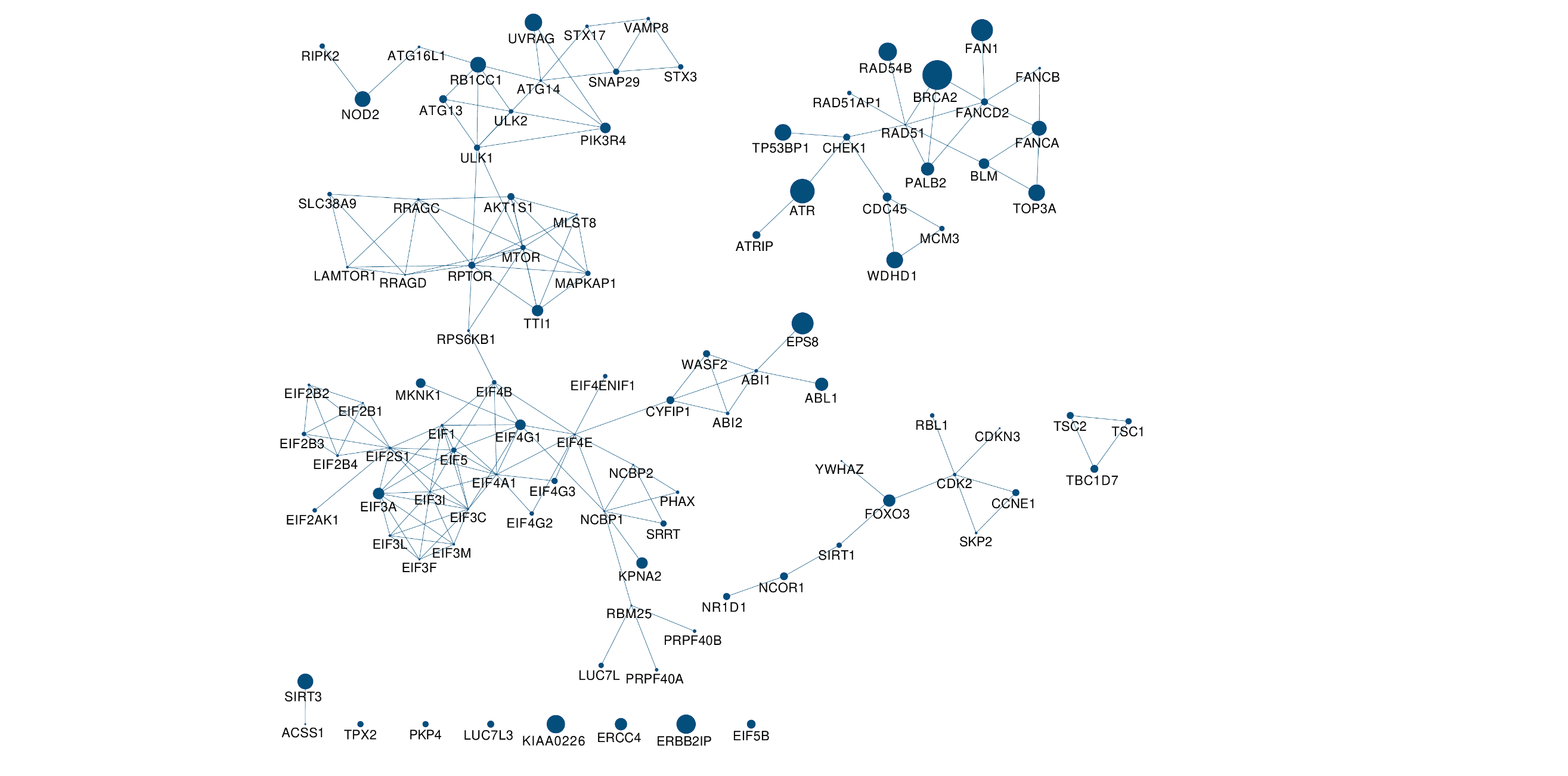
Supplementary Fig. 9** Network representation of Cluster 182, the only significant cluster with *mTOR signaling* as the top-most significant associated pathway. Each node represents a gene in the cluster and the node size indicates the number of ARDS patients harboring qualifying variants in that gene. The lines connecting the nodes represent robust protein-protein interactions according to NHC edge-weighted background network.

## **Supplementary Fig. 10** Network representation of Cluster 12, the only significant cluster with *Amino acids regulate mTORC1* as the top-most significant associated pathway. Each node represents a gene in the cluster and the node size indicates the number of ARDS patients harboring qualifying variants in that gene. The lines connecting the nodes represent robust protein-protein interactions according to NHC edge-weighted background network.
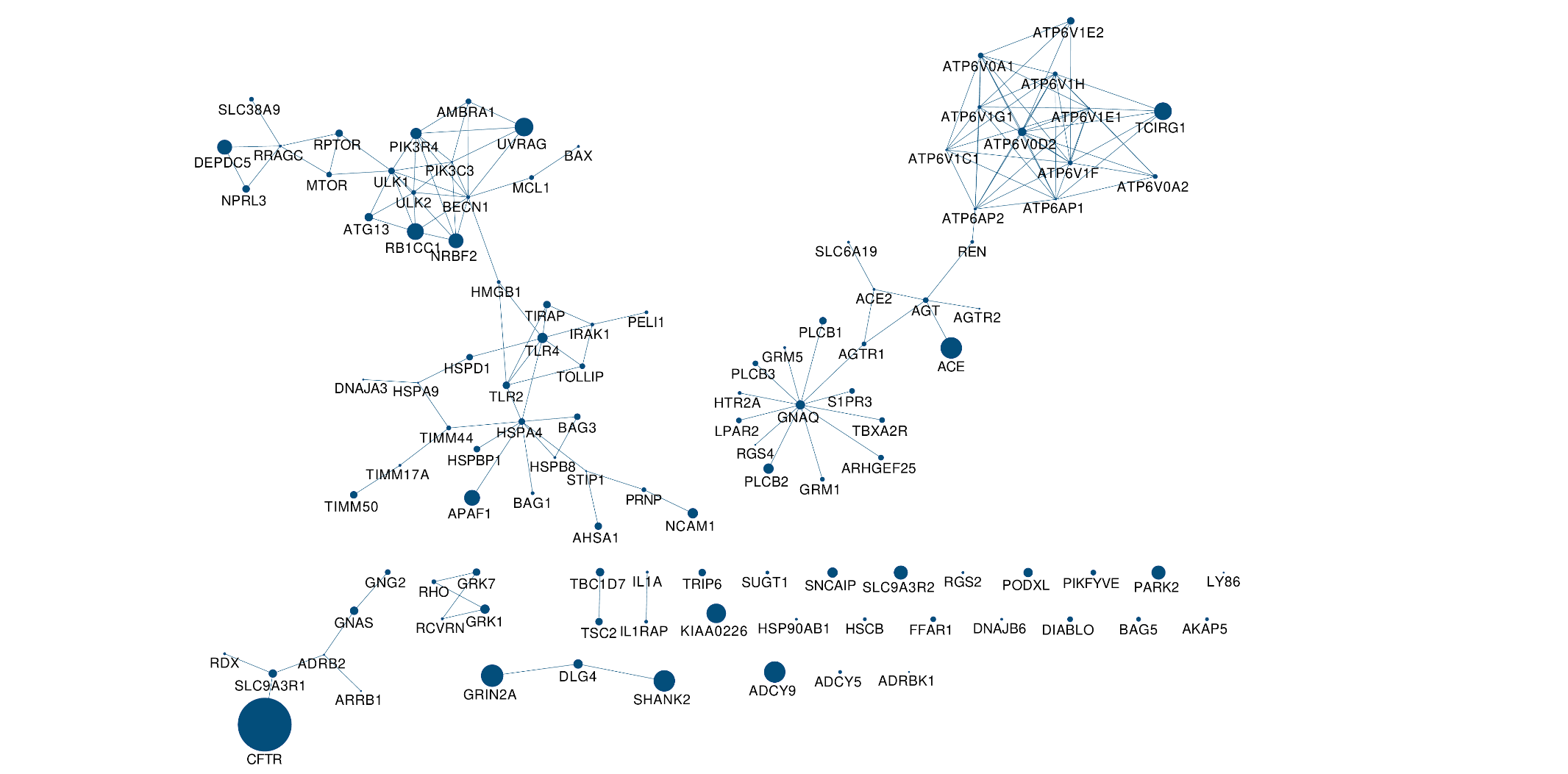


## **Supplementary Fig. 11** Network representation of Cluster 19, the only significant cluster with *Protein-protein interactions at synapses* as the top-most significant associated pathway. Each node represents a gene in the cluster and the node size indicates the number of ARDS patients harboring qualifying variants in that gene. The lines connecting the nodes represent robust protein-protein interactions according to NHC edge-weighted background network.

**
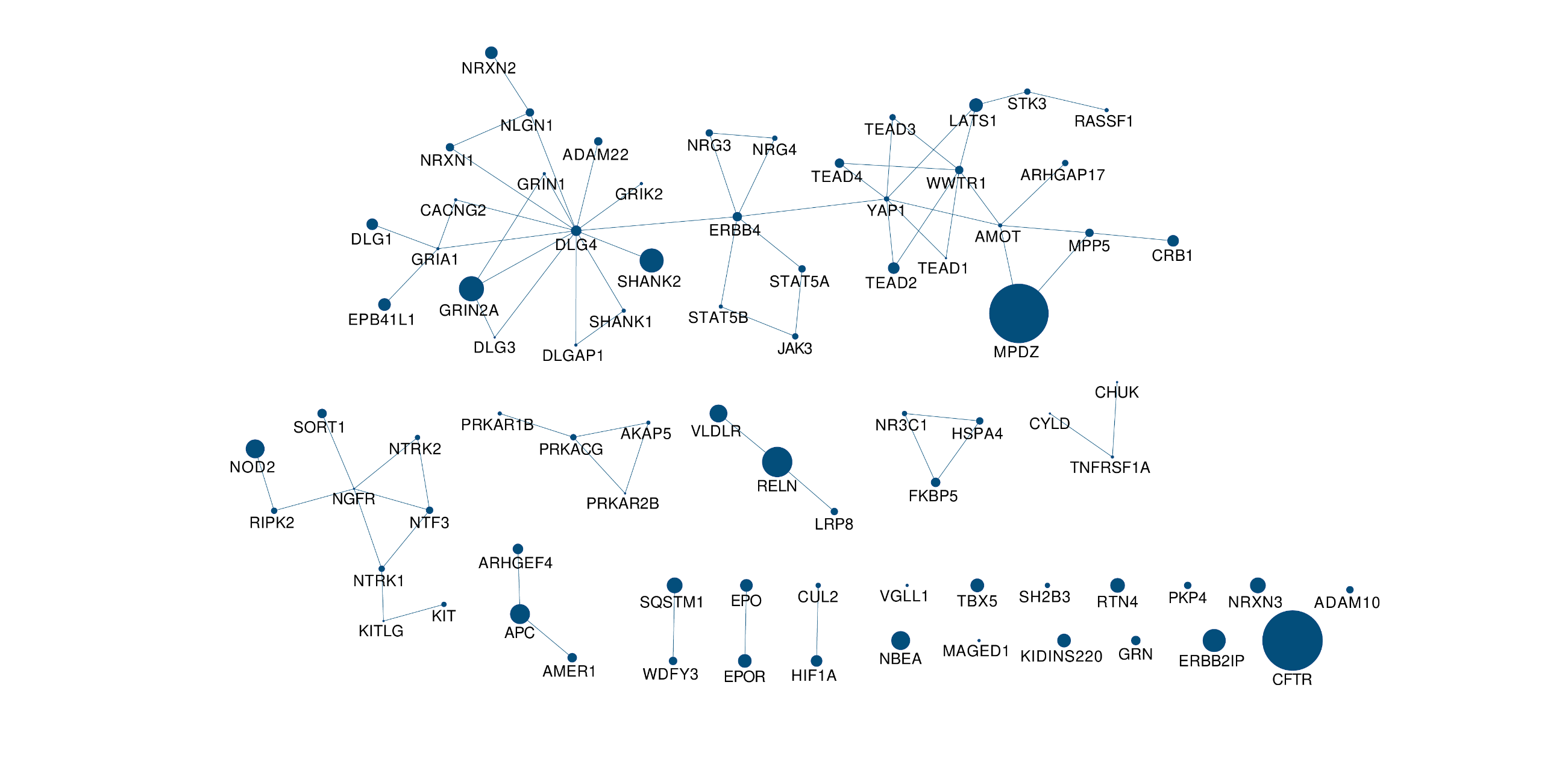
**

## **Supplementary Fig. 12** Network representation of Cluster 46, the most significant among the significant clusters with the *mRNA splicing* as the top significant associated pathway. Each node represents a gene in the cluster and the node size indicates the number of ARDS patients harboring qualifying variants in that gene. The lines connecting the nodes represent robust protein-protein interactions according to NHC edge-weighted background network.


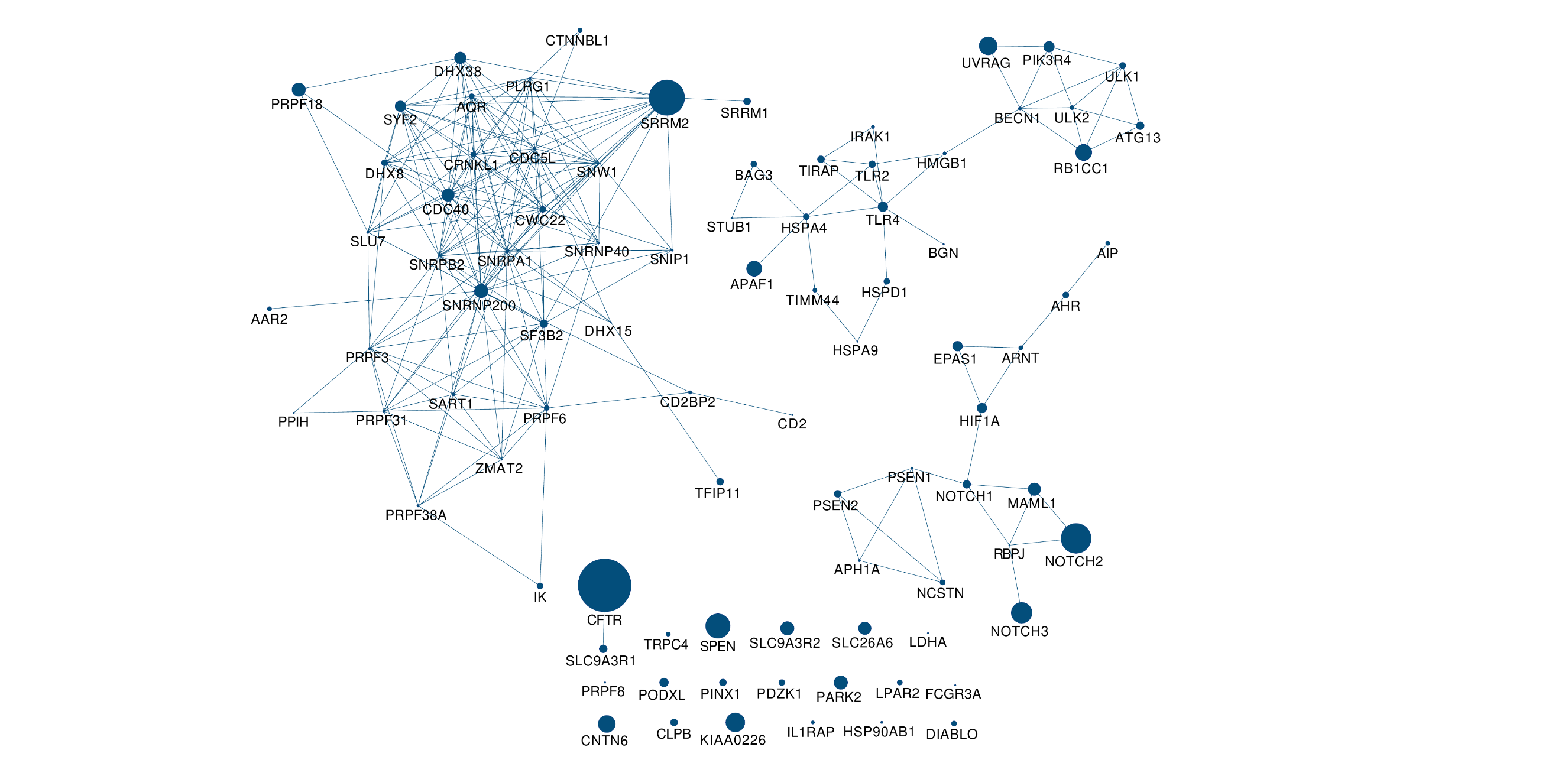


## **Supplementary Fig. 13** Network representation of Cluster 97, the most significant among the significant clusters with the *DNA repair* as the top-most significant associated pathway. Each node represents a gene in the cluster and the node size indicates the number of ARDS patients harboring qualifying variants in that gene. The lines connecting the nodes represent robust protein-protein interactions according to NHC edge-weighted background network.


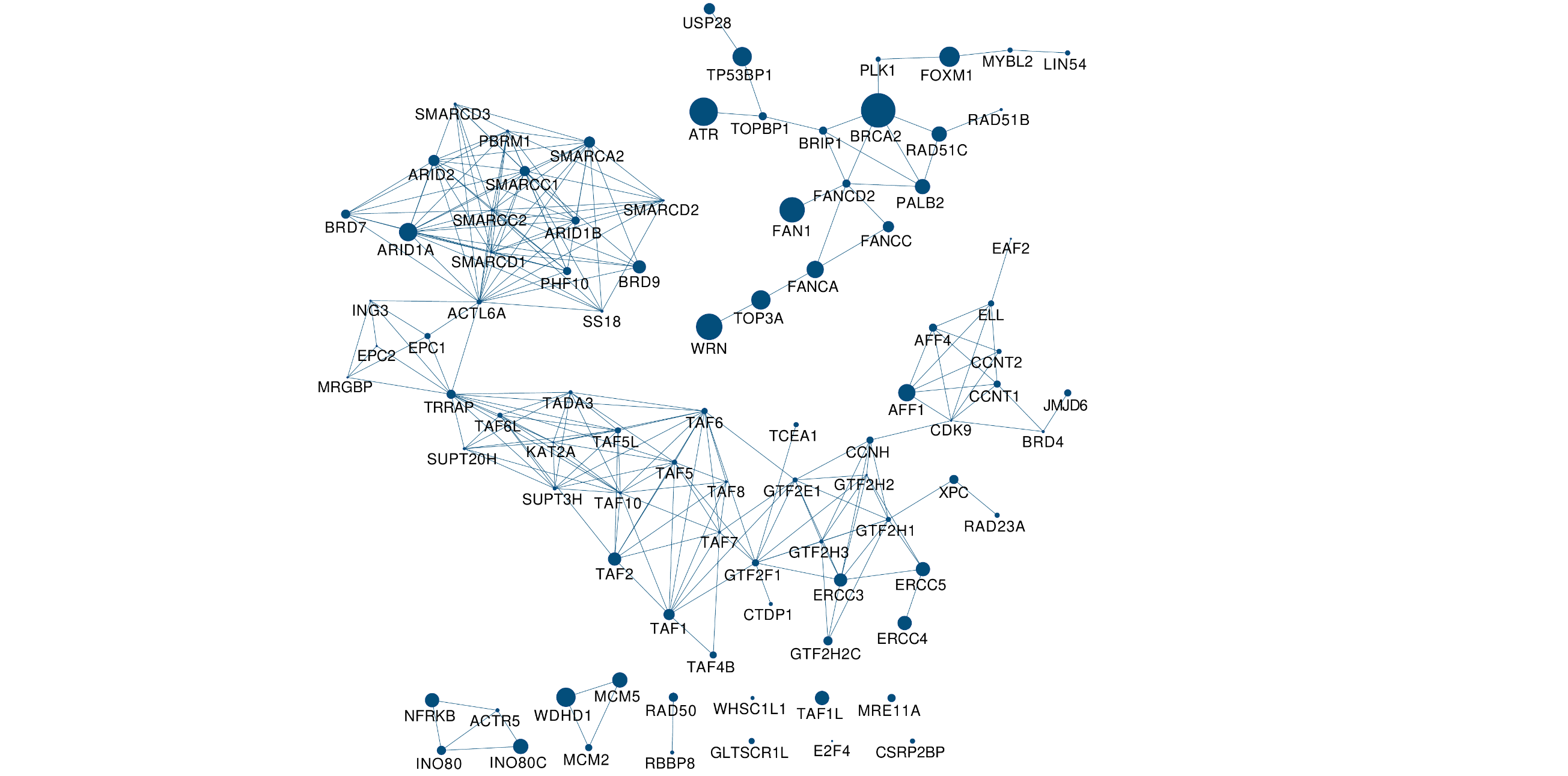

Supplement: Supplementary file 1 — Additional file 1: Includes supplementary methods and references describing workflows for WES and SNP array data, supplementary tables 1-6 and figures 1-13. Supplementary Table 1 Demographics and clinical characteristics of the patients from the GEN-SEP cohort included in this study; Supplementary Table 2 Enriched significant pathways in the significant gene clusters; Supplementary Table 3 Genes exhibiting qualifying variants in at least 10 ARDS cases in the study; Supplementary Table 4 Qualifying variants found in FLT1 in the GEN-SEP cohort patients; Supplementary Table 5 Qualifying variants in the ten most frequent genes observed in the ARDS cases. The number of carriers in ARDS cases and in sepsis controls, along with standardized identifiers, are indicated; Supplementary Table 6 Odds ratios resulting from sensitivity analysis using logistic regressions of patient-recorded variables for the ARDS risk-associated gene clusters with minimal similarity. Results from the stricter model, adjusted for sex, age, and APACHE II scores, excluding and including first 2 principal components, and from models that did not adjust for any covariate are included for reference; Supplementary Table 7 Results from sensibility analyses of QVs with AF≤ 0.01 and AF ≤ 0.001 in gene clusters with minimal similarity. The models were adjusted for sex, age and APACHE II scores; Supplementary Table 8 Results of the null model based on logistic regression analysis of rare synonymous variants in gene clusters with minimal similarity. The models were adjusted for sex, age and APACHE II scores. Supplementary Fig. 1 Plot of the first two principal components of genetic variation of GEN-SEP patients and of data from the 1000 Genomes Project from African, American, East Asian, European, and South Asian. GEN-SEP cases and controls are indicated with differentiated symbols. The percentages represent the explained variability of each axis; Supplementary Fig. 2 Schematic overview of the sequencing workf [file 12931_2026_3588_MOESM1_ESM.docx]
